# MDMX reprograms glycolysis of hepatocellular carcinoma via 14-3-3γ/FOXO1

**DOI:** 10.1038/s41420-025-02804-2

**Published:** 2025-11-07

**Authors:** Han Chen, Qilong Pan, Meiqi Mao, Wu Lin, Sisi Yan, Jie Liu, Shuoqi Lin, Qin Li, Sihui Xue, Yixuan Xie, Lincan Ding, Dali Zheng, Jie You, Qingling Huang

**Affiliations:** 1 Department of Biochemistry and Molecular Biology, School of Basic Medical Sciences, Fujian Medical University, Fuzhou, China; 2 Department of Endoscopic Center, Fuzhou University Affiliated Provincial Hospital, Fuzhou, China; 3 Fujian Key Laboratory of Oral Diseases, School and Hospital of Stomatology, Fujian Medical University, Fuzhou, China; 4 Department of Endocrinology and Metabolism, Fujian Medical University Union Hospital, Fuzhou, China

**Keywords:** Cancer metabolism, Mechanisms of disease

## Abstract

MDMX serves as a significant regulator of p53, which is a crucial tumor suppressor protein. However, the biological functions and underlying mechanism of MDMX in hepatocellular carcinoma (HCC) remain inadequately understood. In this study, we demonstrate that MDMX is overexpressed in HCC, and elevated expression of MDMX is significantly correlated with poor prognosis in HCC harboring mutant p53. MDMX inhibits the degradation of 14-3-3γ and facilitates its localization within cytoplasm, thereby enhances the interaction between FOXO1 and 14-3-3γ, which promotes the degradation of FOXO1. Consequently, the overexpression of MDMX results in downregulation of FOXO1 followed by increase of RPIA and decrease of PCK1, leading to increased glucose uptake, lactate secretion, and ATP production. These findings elucidate the role of MDMX in promoting glycolysis through the regulation of the 14-3-3γ/FOXO1 axis in p53-mutated HCC, thereby offering a potential therapeutic target for the treatment of HCC.

## Introduction

Hepatocellular carcinoma (HCC) is the most frequent type of primary liver cancer; the incidence remains the sixth most common in malignant tumors and the mortality rate ranks third leading cause of cancer-related deaths in the world [1]. Surgical resection, liver transplantation, and chemotherapy are the dominant strategies for the treatment of HCC. But the prognosis of HCC is still very poor because of difficult detection of HCC at early stage. As one of tyrosine kinase inhibitors, sorafenib is used as first-line treatment of advanced HCC [2]. However, sorafenib therapeutic effect can be seriously dampened by the fast occurrence of sorafenib resistance in HCC [3]. Therefore, it is urgent to identify some new effective molecular pathway target for more treatment options of HCC.

The p53 is one of the most vital tumor suppressor proteins, plays a critical role in regulating cell proliferation, differentiation, and apoptosis, etc. p53 is mutated in more than 50% of human tumors, mutant p53 loses its canonical transcriptional activity and gains new functions that can drive tumor progression [4]. Mouse double minute 2 (MDM2/HDM2) and its homolog MDMX (also known as HDMX/MDM4/HDM4) are two important regulatory proteins that control p53 cellular protein levels by targeting it for proteasomal degradation and inhibit p53 transcriptional activity by preventing it from activating transcription [5]. It was reported that MDM2 was involved in cell cycle, cell death, chromatin modification, gene expression, DNA damage, tumorigenesis, invasion, and metastasis in p53-negative or mutant p53-expressing cell lines [5]. Similar to MDM2, increasing evidence showed that MDMX had p53-independent functions related to tumor malignant transformation. Overexpression of MDMX in a mutant p53R172H heterozygous background enhanced tumor development and decreased survival, p53-null mice with high level MDMX tended to have multiple tumors [6]. MDMX overexpression in triple-negative breast cancer with mutant p53 enhanced circulating tumor cells and promoted tumor metastasis [7]. Furthermore, MDMX was found to bind CK1α, inhibit its activity of β-catenin S45 phosphorylation and stimulate the Wnt signaling pathway in a p53-independent manner [8, 9].

The exact molecular mechanisms of p53-independent MDMX oncogenic functions remain ambiguous. Transcriptome data by RNAseq upon MDMX depletion showed that the transcriptional regulation of multiple genes did not only rely on p53, but also FOXO transcription factors, the function of MDMX appeared to be partly dependent on FOXO proteins [10]. FOXO1 is demonstrated to be one of important transcriptional factors involved in a wide range of biological processes, such as apoptosis and autophagy, cell cycle arrest, and metabolism [11]. FOXO1 regulates hepatic glucose metabolism by stimulating expression of gluconeogenic genes (G6Pase, PEPCK) and suppressing expression of genes involved in glycolysis (glucokinase), the pentose phosphate shunt pathway (ribose-5-phosphate isomerase, transketolase), and lipogenesis (SREBP-1) [12]. After phosphorylated by Akt, p-FOXO1 is exported from the nucleus, trapped in cytoplasm by binding with 14-3-3 protein, and degraded to inhibit its transcriptional activity [13]. In response to stress signals, such as metabolic stress and DNA damage signals, MDMX is phosphorylated at S342, S367, and S402 by ATR-Chk1 or ATM-Chk2 kinase cascade, which triggers binding between MDMX and 14-3-3, leading to MDMX inactivation and p53 activation [14, 15]. Whether the binding between MDMX and 14-3-3 affects interaction and function of 14-3-3 and FOXO1 has not been explored.

To investigate the potential effect and the physiological relevance of MDMX, 14-3-3, and FOXO1 in tumorigenesis and progression of HCC, we assess the function and interaction of MDMX, 14-3-3, and FOXO1 in p53 null or p53 mutant HCC cell lines. The expression level of MDMX and 14-3-3γ is higher in HCC than its corresponding adjacent tissues, but the expression of FOXO1 is opposite. MDMX promotes binding of FOXO1 and 14-3-3γ, leading to FOXO1 degradation and inactivation. Transgenic MDMX overexpression mouse is used to confirm the previous results again. The results identify a new activity of MDMX of p53-independent functions and a potential therapeutic target for HCC.

## Results

### MDMX is highly expressed in HCC and promotes growth and proliferation of HCC cells harboring mutant p53

**Fig. 1:**
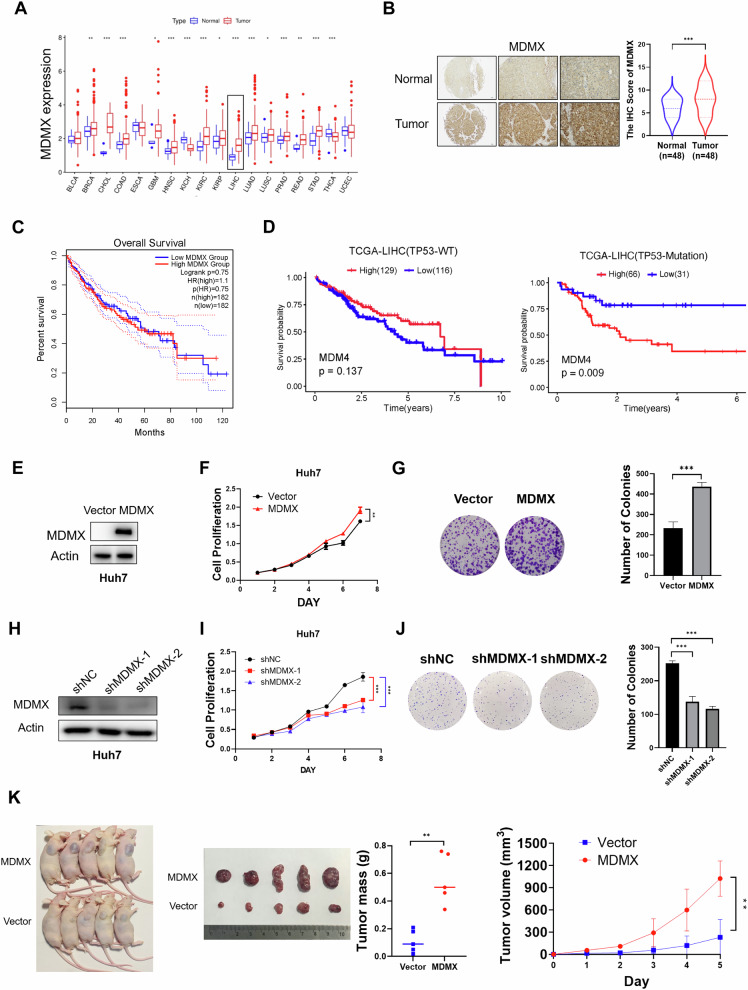
MDMX is overexpressed in LIHC and promotes proliferation of HCC cells harboring mutant p53. **A** Different expressions of MDMX in pan-cancer and normal tissues based on TCGA databases. **B** Immunohistochemical analysis of MDMX expression conducted on tissue microarrays containing tumor samples from 48 patients with hepatocellular carcinoma alongside corresponding normal liver tissues (*n* = 48). **C** Kaplan–Meier survival analysis in HCC patients, *n*(high) = 182; *n*(low) = 182. **D** Kaplan–Meier survival analysis in HCC patients with wild-type, *n*(high) = 129; *n*(low) = 116, or mutant p53, *n*(high) = 66; *n*(low) = 31. **E** Western blot was conducted to confirm MDMX overexpression in stable overexpression Huh7 cell lines. **F** Cell viability assessed by CCK8 assay demonstrated the effect of MDMX overexpression on cell growth (*n* = 3). **G** Colony formation assay illustrated the effect of MDMX overexpression on cell proliferation (*n* = 3). **H** Western blot was conducted to confirm MDMX downregulation in Huh7 cell lines. **I** Cell viability assessed by CCK8 assay demonstrated the effect of MDMX knockdown on cell growth (*n* = 3). **J** Colony formation assay illustrated the effect of MDMX knockdown on cell proliferation (*n* = 3). **K** Huh7 cells overexpressing MDMX were injected subcutaneously into nude mice. Graph showed subcutaneous tumors collected from nude mice, weights and volumes of subcutaneous tumors (*n* = 5). **P < 0.01, ***P < 0.001. **B**, **F**, **G**, **K** *T*-test and **I**, **J** One-way ANOVA were used for statistical analysis.

The different expression levels of MDMX were examined in both cancer and normal tissues obtained from the Cancer Genome Atlas (TCGA) database. The analysis indicated that most cancer types, including cholangiocarcinoma, glioblastoma, and liver hepatocellular carcinoma (LIHC), demonstrated a significant overexpression of MDMX in malignant tissues compared to adjacent normal tissues (Fig. 1A). This observation was corroborated by immunohistochemical analysis conducted on tissue microarrays containing tumor samples from 48 patients with HCC alongside corresponding normal liver tissues (Fig. 1B). Elevated MDMX expression was significantly associated with increased Alpha-FetoProtein levels of HCC patients (Fig. S1A). Kaplan–Meier survival analysis was undertaken to compare the overall survival of different groups via the R package survival and survminer (determined the optimal cutpoint). MDMX expression did not significantly affect the overall survival of LIHC patients (*P* = 0.75) (Fig. 1C). Further analysis revealed a strong association between elevated MDMX levels and poor prognostic outcomes in patients harboring mutant p53 (*P* = 0.009) (Fig. 1D). Collectively, these results suggest that MDMX may serve as a critical factor in liver cancer characterized by p53 mutations.

To further investigate the role of MDMX in HCC, we established stable cell lines with either overexpression or knockdown of MDMX, Western blot was used to check the expression level of MDMX (Fig. 1E, H). Results from CCK-8 and colony formation assays indicated that ectopic expression of MDMX significantly promoted cell proliferation in Huh7 and Hep3B cell lines (Figs. 1F, G and S1B). Conversely, downregulation of MDMX inhibited the growth of cancer cells in both Hep3B and Huh7 lines (Figs. 1I, J and S1C). Additionally, in vivo xenograft models were developed through subcutaneous injection in nude mice. Tumors overexpressing MDMX exhibited a significant accelerated growth rate compared to control group (Fig. 1K), no significant body weight differences between these groups were observed (Fig. S1D).

### MDMX promotes glycolysis in p53 mutant liver cancer cells

**Fig. 2:**
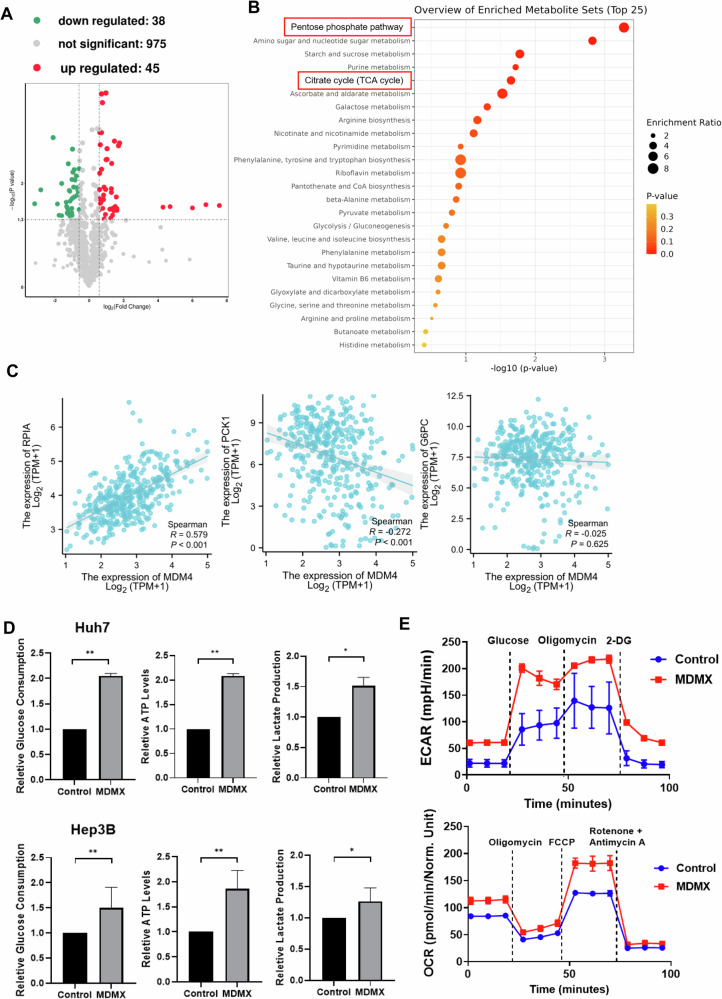
MDMX promotes HCC progression through the activation of glycolysis. **A** Volcano plot for untargeted metabolomic analysis of Huh7 cell lines transfected with either MDMX or vector. **B** Metabolite sets enrichment analysis of the MDMX-transfected Huh7 cells. **C** The correlations between MDMX and genes associated with glucose metabolism. **D** In Huh7 and Hep3B cells overexpressing MDMX, the levels of glucose uptake, ATP, and lactate production in the cells were measured. **E** In Huh7 cells overexpressing MDMX, the oxygen consumption rate and extracellular acidification rate of the cells were measured using Seahorse XFp. *P < 0.05, **P < 0.01. **D** *T*-test was used for statistical analysis.

To elucidate the mechanisms by which MDMX facilitates HCC progression, Huh7 cells were transfected with either MDMX or vector, followed by an untargeted metabolomic analysis. The resulting metabolomic profiles indicated a distinct separation in metabolites between the MDMX-transfected group and the control group (Fig. S2A). Specifically, MDMX transfection resulted in the downregulation of 38 metabolites and the upregulation of 45 metabolites (Fig. 2A). Metabolite sets enrichment analysis of the MDMX-transfected Huh7 cells identified the pentose phosphate pathway as the most significantly activated pathway, and citrate cycle ranking fifth (Fig. 2B). TCGA-LIHC database analysis revealed that several genes expression associated with glucose metabolism exhibited correlations with MDMX. Notably, MDMX was positively correlated with RPIA, PKM, SLC2A1 (also known as GLUT1), and HK2 (*P* < 0.001), and negatively correlated with PCK1 and AQP9 (*P* < 0.001) (Figs. 2C and S2B). Among these, RPIA serves as a critical enzyme in the regulation of the pentose phosphate pathway, whereas PCK1, G6Pase, and AQP9 are involved in gluconeogenesis regulation. Conversely, PKM, GLUT1, and HK2 are implicated in glycolysis regulation. These findings suggested that MDMX may be involved in aerobic glycolysis and gluconeogenesis in HCC cells.

To further assess the impact of MDMX on glycolysis, we evaluated glucose consumption in the presence or absence of MDMX. The overexpression of MDMX resulted in a significant increase in glucose consumption, ATP levels, and lactate production (Fig. 2D), while downregulation of MDMX led to a reduction of these items (Fig. S2C). Measurements of the extracellular acidification rate (ECAR) and oxygen consumption rate (OCR) corroborated that MDMX overexpression enhanced both the basal ECAR and the overall maximal cellular respiration (Fig. 2E), while the knockdown of MDMX led to the opposite result (Fig. S2D). In summary, our data indicated that MDMX may activate glycolysis, thereby contributing to the progression of HCC.

### MDMX regulates the expression of PCK1 and RPIA by promoting degradation of FOXO1

**Fig. 3:**
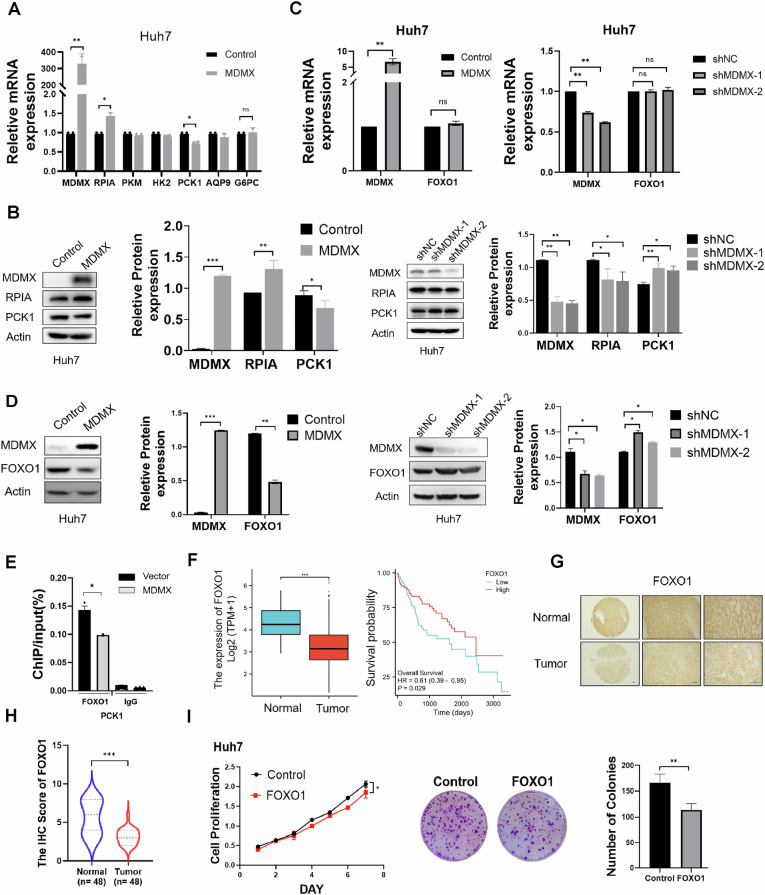
MDMX regulates the expression of PCK1 and RPIA in FOXO1 pathway. **A** In Huh7 cells with overexpression of MDMX, mRNA level of RPIA, PKM, HK2, PCK1, AQP9, and G6PC were detected by q-PCR. **B** In Huh7 cells with overexpression or knockdown of MDMX, the protein level of PCK1 and RPIA were measured by Western blot. **C** In Huh7 cells with overexpression of MDMX, mRNA level of FOXO1 was detected by q-PCR. **D** In Huh7 cells with overexpression or knockdown of MDMX, the protein level of FOXO1 were measured by Western blot. **E** ChIP-qPCR was conducted to analyze the combination of FOXO1 and the promoter of PCK1 in Huh7 cells with MDMX overexpression. **F** TCGA analysis of FOXO1 expression level in HCC tissues (*n* = 50) compared with adjacent tissues (*n* = 374) and the relationship to patient’s overall survival. **G**, **H** Immunohistochemical analysis of FOXO1 expression conducted on tumor samples from 48 patients with HCC alongside corresponding normal liver tissues. **I** CCK-8 and colony formation assay were conducted to study the effect of FOXO1 on cell growth. *P < 0.05, **P < 0.01, ***P < 0.001. **A**–**D**, **F**, and **H**, **I** *T*-test was used for statistical analysis.

To explore the mechanism by which MDMX influences glucose metabolism, the impact of MDMX on the mRNA expression levels of RPIA, PKM, HK2, PCK1, AQP9, and G6PC were tested. Consistent with our correlation analysis, the overexpression of MDMX in Huh7 and Hep3B cells resulted in a significant increase in RPIA expression and decrease in PCK1 expression at both mRNA and protein levels, while no significant changes were observed in the expression of PKM, HK2, and AQP9 (Figs. 3A, B and S3A, B). Given that PCK1 and RPIA are well-established downstream transcriptional targets of FOXO1 [12], we further examined the effect of MDMX on FOXO1 expression. MDMX had minimal effects on the mRNA levels of FOXO1 in Huh7 cells (Fig. 3C), indicating that MDMX modulates FOXO1 primarily at the post-transcriptional level. As illustrated in Figs. 3D and S3C, the overexpression of MDMX led to a marked downregulation of FOXO1 protein levels in Huh7 and Hep3B cells, while knockdown of MDMX significantly upregulated FOXO1 in Huh7 cells. Moreover, as demonstrated in Fig. 3E, MDMX overexpression significantly decreased the binding affinity between FOXO1 and the promoter region of its target gene PCK1. These findings are consistent with the q-PCR results presented in Fig. 3A. Consequently, we hypothesized that the regulation of RPIA and PCK1 by MDMX is contingent upon FOXO1.

Analysis of data from TCGA revealed that FOXO1 is significantly downregulated in HCC, low expression level is correlated with poor prognosis (Fig. 3F). Low expression of FOXO1 in HCC was corroborated by immunohistochemical analysis conducted on tissue microarrays containing 48 paired tumors and normal samples from HCC patients (Fig. 3G, H). As a tumor suppressor gene, FOXO1 was shown to inhibit the proliferation and clonogenic potential of tumor cells (Fig. 3I).

**Fig. 4:**
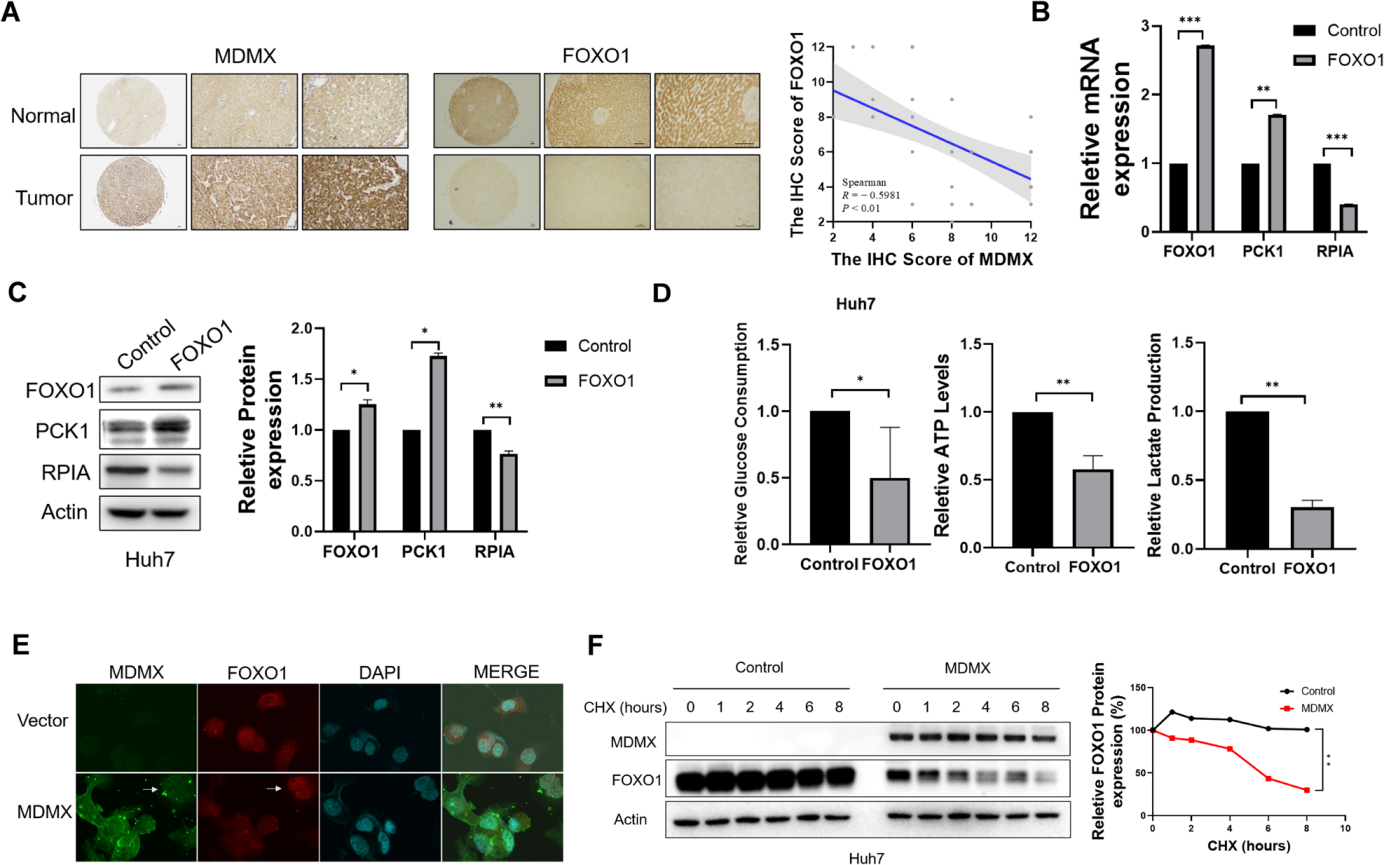
MDMX enhances glycolysis by promoting the degradation of FOXO1. **A** Correlation analysis of MDMX and FOXO1 expression using immunohistochemical scoring (*n* = 48). **B**, **C** mRNA and protein expression levels of PCK1 and RPIA after FOXO1 overexpression in Huh7 cells. **D** Glucose uptake, ATP levels, and lactate production were examined after FOXO1 overexpression in Huh7 cells. **E** Immunofluorescence images showed FOXO1 downregulation after MDMX overexpression. **F** Protein level of FOXO1 was measured by Western blot in Huh7 cells with overexpression of MDMX treated with 10 µM CHX. *P < 0.05, **P < 0.01, ***P < 0.001. **B–D**, **F** *T*-test was used for statistical analysis.

Furthermore, a combined analysis of immunohistochemical scores for MDMX and FOXO1 demonstrated a significant negative correlation between two proteins (*P* < 0.01, *R* = −0.5981) (Fig. 4A). In agreement with previous studies [12], we reaffirmed that FOXO1 can downregulate RPIA while upregulate PCK1 at both mRNA and protein levels (Fig. 4B, C). Additionally, we assessed intracellular glucose consumption, ATP levels, and lactate production following FOXO1 overexpression (Fig. 4D), further supporting the conclusion that FOXO1 inhibited glycolysis. Immunofluorescence and a cycloheximide (CHX) chase assay were used to examine the effect of MDMX on FOXO1 protein level, the data showed that MDMX may facilitate the degradation of FOXO1 (Fig. 4E, F). In summary, these findings collectively indicated that MDMX promoted the degradation of FOXO1, thereby affected expression of PCK1 and RPIA and enhanced glycolysis.

### MDMX localizes the 14-3-3γ in cytoplasm and enhances the interaction between FOXO1 and 14-3-3γ to promote degradation of FOXO1

**Fig. 5:**
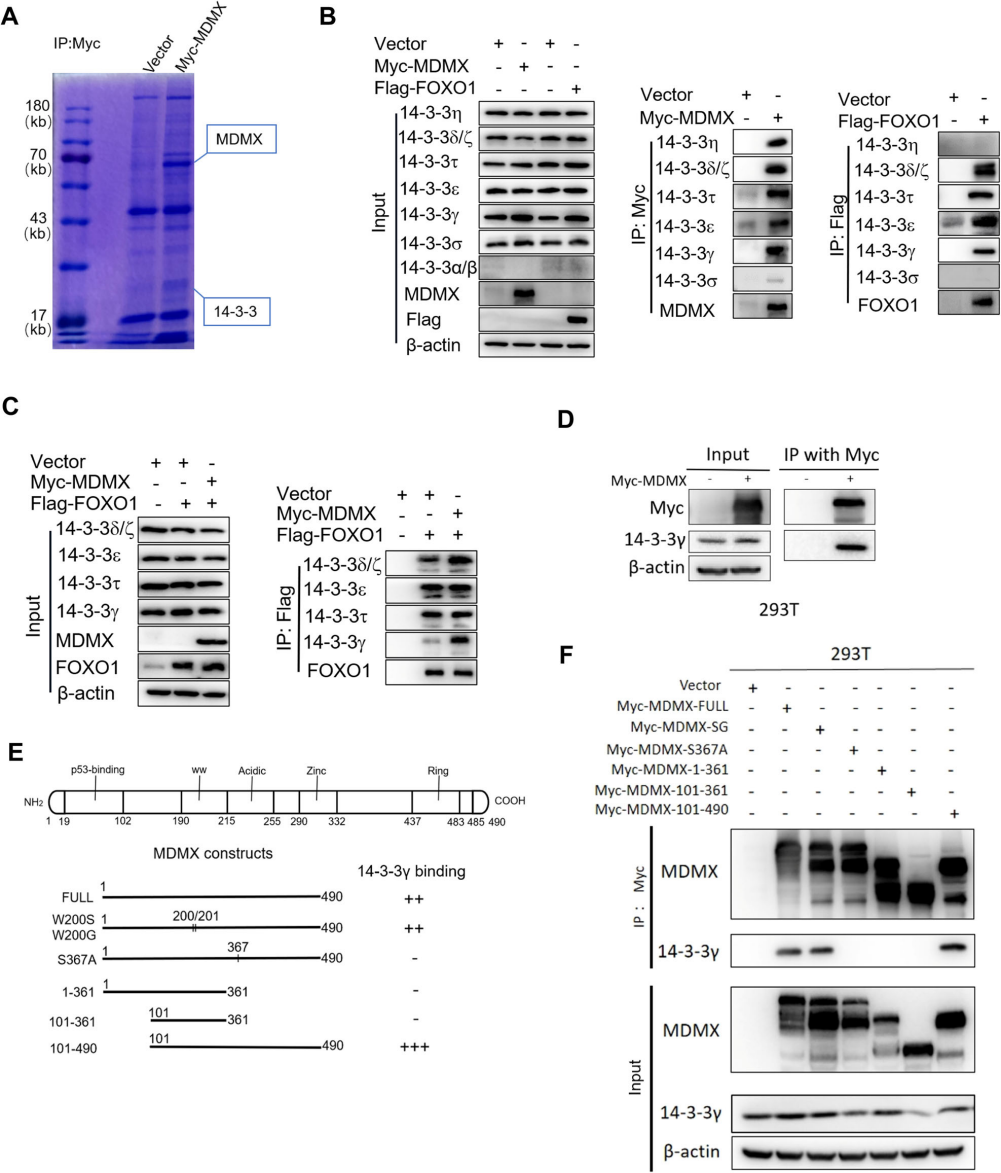
MDMX enhances the interaction between FOXO1 and 14-3-3γ. **A** Coomassie blue staining of proteins immunoprecipitated by Myc antibody in 293 T. **B** Co-Immunoprecipitation exploring the interaction between isoforms of the 14-3-3 and MDMX or FOXO1 in Huh7 cells. **C** Co-Immunoprecipitation exploring the effect of MDMX overexpression on the interaction between isoforms of the 14-3-3 and FOXO1 in Huh7 cells. **D** Co-Immunoprecipitation was used to verify the interaction between MDMX and 14-3-3γ. **E** Schematic diagram of MDMX full length, truncations, and mutants. **F** Full-length, truncated, mutant plasmids of MDMX were transfected into 293 T cells, and their interactions with 14-3-3γ were detected by Co-Immunoprecipitation.

The co-immunoprecipitation data revealed that MDMX does not directly interact with FOXO1 (Fig. S3D). To further explore, we immunoprecipitated MDMX using a Myc tag antibody in 293 T cells with overexpression of Myc-MDMX and employed mass spectrometry to analyze potential interacting proteins (Fig. 5A). Through literature review and data analysis, we identified MDMX could bind to different isoforms of 14-3-3 (Fig. S4A). Due to the intrinsic 14-3-3α/β protein in Huh7 cells was barely detected, the interaction between 14-3-3α/β and MDMX or FOXO1 was not checked. The co-immunoprecipitation results revealed that several isoforms of the 14-3-3 protein are capable of binding to both MDMX and FOXO1 except for 14-3-3η and 14-3-3σ (Fig. 5B). We co-transfected Huh7 cells with MDMX and FOXO1 to explore the effect of MDMX on the interaction between FOXO1 and several isoforms of 14-3-3. Surprisingly, MDMX significantly enhances the interaction between FOXO1 and 14-3-3γ (Fig. 5C). Previous research has established that AKT phosphorylates a highly conserved residue of FOXO1, which serves as a chaperone binding site for 14-3-3 proteins [16]. Additionally, the serine 367 residue of MDMX is phosphorylated by wild-type Chk1, which enhances the binding of 14-3-3γ to MDMX, resulting in its cytoplasmic localization [14, 15]. These findings prompted us to investigate whether the effect of MDMX on FOXO1 protein level is dependent on 14-3-3γ.

Co-immunoprecipitation was used to confirm that MDMX interacts with 14-3-3γ, irrespective of whether it is endogenous or exogenous in 293 T cells (Figs. 5D and S4B). To pinpoint the specific interaction site between 14-3-3γ and MDMX, we constructed three MDMX truncation mutations (1–361, 101–361, and 101–490) and two site-specific mutants, substituting residue 200/201 from tryptophan to serine/glycine (MDMX-SG) and residue 367 from serine to alanine (MDMX-S367A) (Fig. 5E). Co-immunoprecipitation and Western blot analysis demonstrated that MDMX-101–490 deleting N terminus exhibited the strongest binding affinity for 14-3-3γ (Fig. 5F). Both MDMX-SG and full-length MDMX displayed comparable binding affinities for 14-3-3γ. Conversely, no binding was observed between MDMX-101-361, MDMX-1-361, or MDMX-S367A and 14-3-3γ, thereby reaffirming the critical role of serine at position 367 of MDMX in its interaction with 14-3-3γ.

**Fig. 6:**
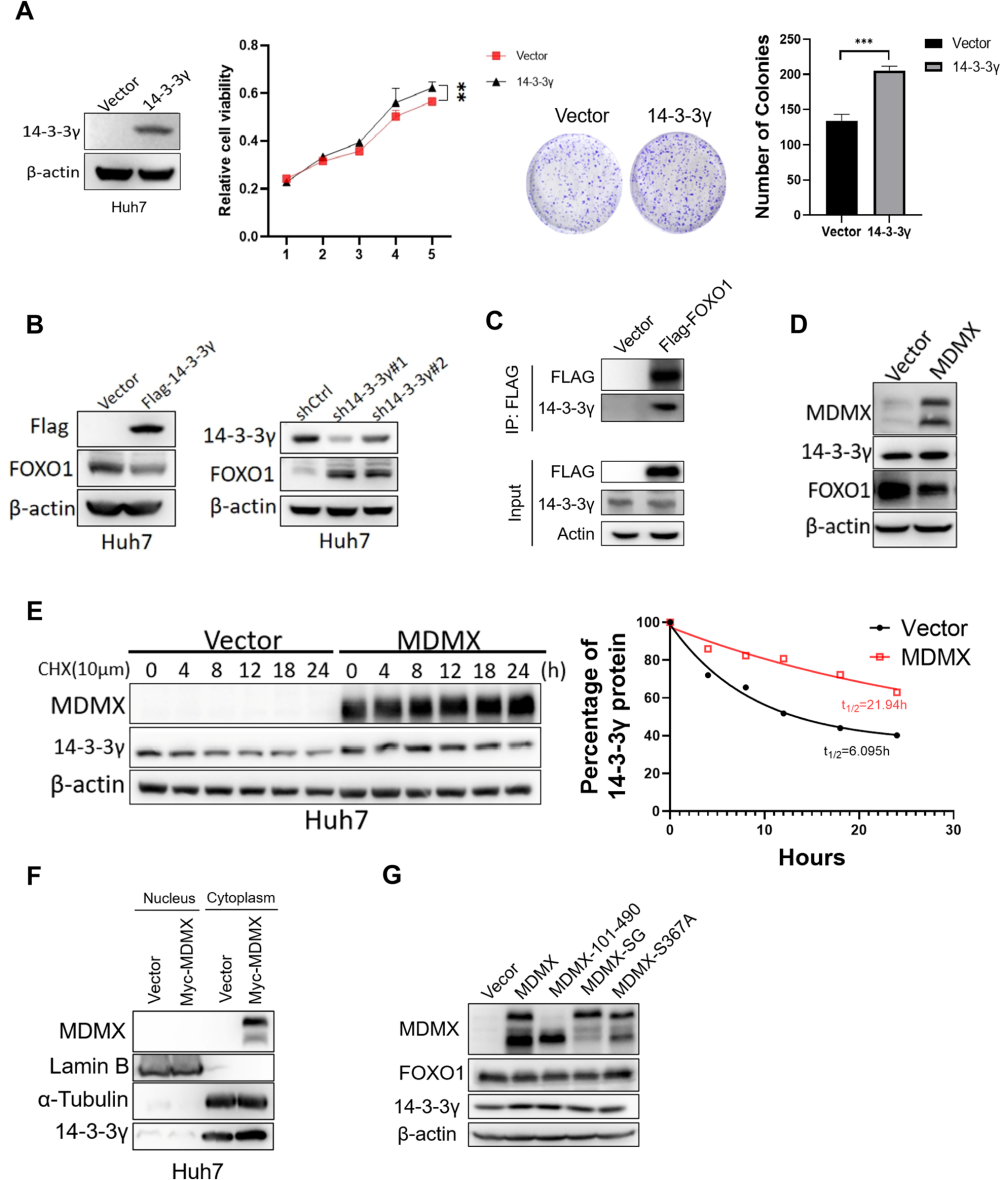
MDMX localizes 14-3-3γ in cytoplasm to accelerate the degradation of FOXO1. **A** Western blot, colony formation, and cell proliferation assays were conducted in Huh7 cells overexpressing 14-3-3γ. **B** Western blot was used to detect FOXO1 protein level after overexpression or knockdown of 14-3-3γ in Huh7 cells. **C** Co-Immunoprecipitation showed the interaction between 14-3-3γ and FOXO1. **D** Western blot showed the effect of MDMX overexpression on expression level of FOXO1 and 14-3-3γ in Huh7 cells. **E** CHX chase assay showed 14-3-3γ degradation kinetics following MDMX overexpression in Huh7 cells. **F** Western blot showed the subcellular localization of 14-3-3γ in Huh7 cells overexpressed MDMX. **G** MDMX, MDMX-101-490, MDMX-SG, and MDMXS367A were overexpressed in Huh7 cells and their effects on FOXO1 protein levels were observed by Western blot. **P < 0.01, ***P < 0.001. **A** *T*-test was used for statistical analysis.

Analysis of data from TCGA revealed that 14-3-3γ was significantly upregulated in HCC (Fig. S4C). CCK-8 and colony formation assays showed that 14-3-3γ promoted cell growth and proliferation (Figs. 6A and S4D), while knockdown of 14-3-3γ inhibited cell growth and proliferation in Huh7 and Hep3B cells (Fig. S4E, F). FOXO1 was shown to be phosphorylated by activated Akt, which inhibited its nuclear translocation by binding to 14-3-3γ, thereby facilitating its ubiquitin-mediated degradation and inhibiting transcriptional activity [16]. Consistent with this, overexpression of 14-3-3γ resulted in decreased expression levels of FOXO1, whereas knockdown of 14-3-3γ with shRNA led to an increase of FOXO1 levels (Figs. 6B and S4G). Immunoprecipitation was conducted in 293 T cells with the ectopic expression of FOXO1, and the results confirmed the interaction between these two proteins (Fig. 6C). Overexpression of MDMX enhanced 14-3-3γ protein level and downregulated the protein level of FOXO1, but did not affect the 14-3-3γ mRNA level (Figs. 6D and S4H). To further investigate the protein stability of 14-3-3γ in MDMX-transfected cells, CHX chase assay was conducted. Our results indicated that MDMX extended the half-life of 14-3-3γ in Huh7 cells (Fig. 6E). The spatial distribution of proteins within cells significantly impacted their function and biological activities. To explore the influence of MDMX on the localization of 14-3-3γ, the subcellular localization of these proteins in Huh7 cells were examined. Our analysis revealed that the majority of 14-3-3γ was present in cytoplasm, and MDMX overexpression may promote its cytoplasmic localization (Fig. 6F). To investigate whether the MDMX-mediated enhancement of the 14-3-3γ and FOXO1 interaction further suppresses FOXO1 levels, full-length MDMX, MDMX-101-490, MDMX-SG, and MDMX-S367A were transfected into Huh7 cells. Western blot analysis demonstrated that both truncations and mutations could reduce FOXO1 levels and elevate 14-3-3γ levels except for MDMX-S367A, which could not bind to 14-3-3γ (Fig. 6G). These data suggests that the effect of MDMX on protein level of FOXO1 is present only if the existence of interaction between MDMX and 14-3-3γ.

### The effect of MDMX overexpression on HCC cells are mitigated by AS1842856 and cells with MDMX overexpression are resistant to 2-DG

**Fig. 7:**
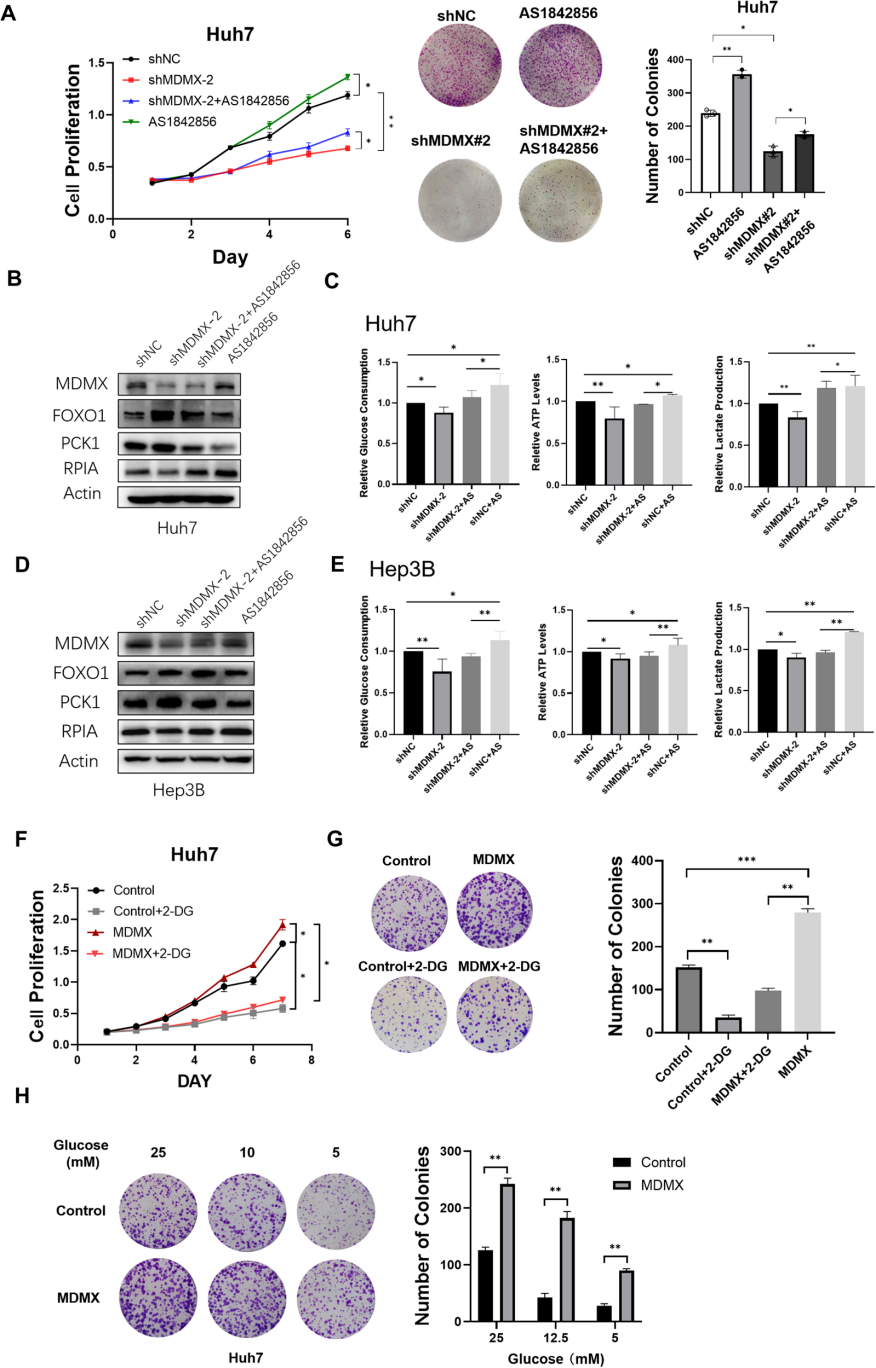
The effect of MDMX overexpression on HCC cells are mitigated by AS1842856 and cells with MDMX overexpression are resistant to 2-DG and low glucose. **A** Colony formation and cell proliferation assays were conducted in Huh7 cells with MDMX knockdown and AS1842856 treatment. **B**, **D** Western blot showed the protein level of MDMX, FOXO1, PCK1, and RPIA after MDMX knockdown and treated with AS1842856 in Huh7 and Hep3B cells. **C**, **E** The level of glucose consumption, ATP level, and lactate production after MDMX knockdown and treated with AS1842856 in Huh7 and Hep3B cells. **F**, **G** Colony formation and cell proliferation assays were conducted in Huh7 cells with MDMX overexpression and 2-DG treatment. **H** Huh7 cells overexpressing MDMX were cultured with different glucose concentration medium (25, 12.5, 5 mM), colony formation ability was measured after 10 days cultural. *P < 0.05, **P < 0.01, ***P < 0.001. **A**–**G** One-way ANOVA and **H** *T*-test were used for statistical analysis.

AS1842856 is a small molecule inhibitor known to interfere with the transcriptional activity of FOXO1 by directly binding to it, thereby inhibiting its function. Based on the results from Western blot analysis and cell viability assays in Huh7 cells treated with different concentration of AS1842856 (Fig. S5A) and literature review [17], a concentration of 0.625 μM was selected for subsequent experiments. HCC cells exhibited a significant reduction in viability following MDMX knockdown when compared to control group. The decrease in cell viability resulting from shRNA was mitigated by treatment with AS1842856 (Figs. 7A and S5B). The protein level of PCK1 decreased after AS1842856 treatment, while the protein level of RPIA increased. Following the knockdown of MDMX, the expression level of FOXO1 increased, leading to upregulation of PCK1 and downregulation of RPIA, FOXO1 inhibitors restored the protein expression levels of both PCK1 and RPIA (Fig. 7B, D). Glucose consumption, ATP levels, and lactate production were significantly diminished after MDMX knockdown, treatment with AS1842856 resulted in an increase of these glucose metabolic indicators (Fig. 7C, E). These data suggested that MDMX partially mediated glycolysis through FOXO1 signaling.

To further investigate the relationship between MDMX’s influence on cellular aerobic glycolysis and its role in tumorigenesis, the glucose analog and small molecule glycolysis inhibitor 2-Deoxy-D-glucose (2-DG) was used. Based on literature review, 6 mM 2-DG was administered to Huh7 cells overexpressing MDMX for 24 h [18]. The results, as illustrated in Fig. 7F, G, demonstrated that the inhibition of glycolysis by 2-DG significantly impaired the growth, proliferation, and clonogenic capabilities of the cells. However, the sensitivity of MDMX-overexpressing cells to 2-DG was notably reduced compared to the control group.

Subsequently, we cultured MDMX-overexpressing Huh7 cells in media with varying glucose concentrations for 24 h to assess their tolerance to low glucose levels. Colony formation assays revealed that MDMX-overexpressing cells exhibited enhanced adaptability to a low-glucose environment (Fig. 7H). These findings confirm that MDMX promotes cell growth and proliferation, at least in part, through its dependence on aerobic glycolysis.

### Overexpression of MDMX enhances the glycolysis via 14-3-3γ/FOXO1 in transgenic mice

**Fig. 8:**
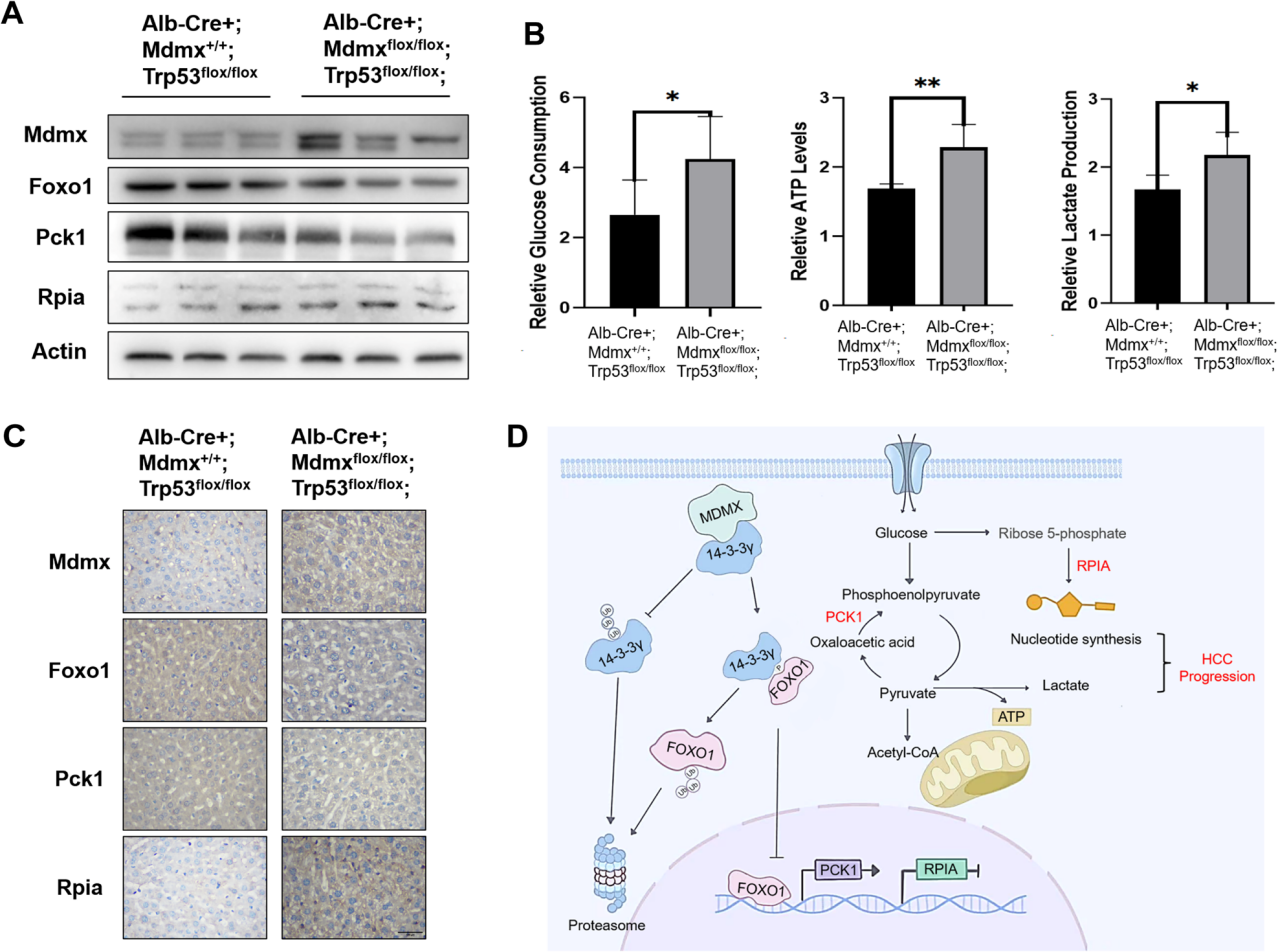
Mouse experiments demonstrate that overexpression of MDMX enhances glycolysis via 14-3-3γ/FOXO1. **A** Western blot showed the expression levels of FOXO1, PCK1, and RPIA in liver tissues from tissue specific expressing MDMX mice. **B** The levels of glucose uptake, ATP, and lactate production in the liver tissues were measured. **C** Immunohistochemistry showed the expression levels of FOXO1, PCK1, and RPIA in liver tissues from tissue specific expressing MDMX mice. **D** Mechanism diagram of how MDMX promotes the occurrence and development of liver cancer by promoting glycolysis via 14-3-3γ/FOXO1. *P < 0.05, **P < 0.01. **B** *T*-test was used for statistical analysis.

In this study, conditional transgenic mouse model which overexpressed Mdmx with Trp53-R172H mutant in liver was employed to investigate the influence of MDMX on glucose metabolism. Genotyping was conducted to identify mouse genotype (Fig. S6A). Liver tissues were harvested from 6 to 8-week-old Trp53-R172H and Mdmx double-positive mice to assess the effects of Mdmx overexpression on Foxo1. Western blot analysis confirmed that Mdmx expression was indeed elevated in the Trp53-R172H and Mdmx double-positive mice. Notably, the expression levels of Foxo1 and Pck1 in the liver tissues of these Mdmx-overexpressing mice were significantly diminished relative to the control group, while the protein expression level of Rpia was found to be increased in the results of Western blot and Immunohistochemistry (Figs. 8A, C and S6B). The findings presented in Figs. 8B and S6C reveal that the liver tissues of Mdmx-overexpressing mice exhibited significant increase in glucose uptake, ATP levels, and lactate production. These results suggest that Mdmx enhances aerobic glycolysis in conditional transgenic mice, thereby providing further evidence that MDMX may influence the glucose metabolic reprogramming of liver cancer cells via the FOXO1 pathway.

## Discussion

MDMX is a prominent oncoprotein that, as a homolog of MDM2, plays a critical role in forming dimers with MDM2, which facilitates the degradation of wild-type p53 and inhibits its transcriptional activities. However, the functions of MDMX that are independent of wild-type p53 remain underexplored. MDM2 was known to induce the ubiquitination and degradation of the E3 ligase SPSB2, which in turn stabilized inducible nitric oxide (NO) synthase and enhances NO production. This process led to the s-nitrosylation of NO and the activation of hypoxia-inducible factor 1-alpha, thereby initiating glycolytic and pro-inflammatory pathways in M1 macrophages [19]. Furthermore, MDM2-mediated activation of Notch1 has been identified as a mechanism contributing to hyperglycemia-induced proliferation of glomerular mesangial cells and the accumulation of extracellular matrix (ECM) [20]. The interaction between Notch1 and TAZ has been shown to promote aerobic glycolysis and facilitate immune evasion in lung cancer [21]. Additionally, the phosphorylation of MDM2 by AKT, which also phosphorylated FOXO1 and FOXO3, was associated with their subsequent ubiquitination and degradation [22]. MDMX has been reported to interact with UXT, inhibiting wild-type p53 and leading to the activation of nuclear factor kappa-light-chain-enhancer of activated B cells (NF-κB), thereby promoting glycolysis [23]. Both MDMX and its homolog MDM2 have been implicated in the promotion of aerobic glycolysis. MDMX was shown to downregulate the transcriptional activity of FOXO, consequently inhibiting the transcription of its downstream target genes; however, the underlying mechanisms remain to be elucidated [10]. Fu et al. proposed that the p53-binding domain of MDM2 plays a pivotal role in mediating the interaction between MDM2 and FOXO [22]. Given the structural similarity between MDMX and MDM2, particularly their shared p53-binding domain, but the interaction between MDMX and FOXO1 was not detected. While MDM2 facilitated the degradation of FOXO1 through its E3 ubiquitin ligase activity, MDMX has been observed to downregulate FOXO1 protein levels independently of E3 ligase activity. This suggests that MDMX likely employs a distinct mechanism to suppress FOXO1 protein expression.

As a transcription factor, the FOXO protein family plays a critical role in regulating diverse biological processes, including development, metabolism, stem cell maintenance, and lifespan. Due to its ability to modulate cell proliferation, angiogenesis, and cell migration, FOXO proteins are considered promising therapeutic targets for cancer treatment [10]. Further investigations into the FOXO protein family have revealed that the subcellular localization of FOXO proteins is tightly regulated by the phosphorylation status of specific serine/threonine residues. For instance, activation of the Akt pathway through the insulin/PI3K signaling cascade, as well as phosphorylation by glucocorticoid-induced kinases (SGKs), promotes the binding of FOXO proteins to 14-3-3 proteins, leading to their nuclear export and subsequent degradation via the ubiquitin/proteasome-mediated pathway [24]. FOXO1 serves as a substrate for multiple E3 ubiquitin ligases, including SKP2, CHIP, and COP1. Following AKT-mediated phosphorylation, FOXO1 undergoes distinct ubiquitination cascades: (1) COP1 and CHIP mediate its ubiquitination and subsequent proteasomal degradation [25, 26], while (2) 14-3-3γ binding facilitates nuclear export, exposing FOXO1 to cytoplasmic E3 ligases SKP2 and MDM2 for additional ubiquitination and degradation [22, 27]. The literature indicates a triple mutant of FOXO1 (T24A/S256A/S319A), which cannot be phosphorylated by AKT, is predominantly nuclear and constitutively active because interactions with 14-3-3 proteins are abolished [28]. Additionally, 14-3-3η has been implicated in the ECM degradation of fibroblast-like synovial cells in rheumatoid arthritis by facilitating the nuclear export of FOXO3 [29]. In this study, we demonstrate that MDMX interacts with 14-3-3γ, sequestering 14-3-3γ in the cytoplasm. This interaction disrupts the nuclear localization of FOXO1, resulting in its cytosolic retention and subsequent ubiquitination-mediated degradation.

In tumor cells, the phenomenon characterized by a preference for glycolysis as the primary metabolic pathway, even in the presence of sufficient oxygen and functional mitochondria, is termed the Warburg effect [30]. Aerobic glycolysis was initially identified in HCC and has since been recognized as a critical regulator of HCC growth, proliferation, invasion, metastasis, and drug resistance [17]. HCC ranks as the fifth most common cancer globally and is the third leading cause of cancer-related mortality [1]. Given that the liver is a central metabolic organ, impaired liver function is frequently associated with dysregulation of glucose and lipid metabolism [31]. The alterations in glucose metabolism observed in liver cancer include the following key features: enhanced aerobic glycolysis, leading to the conversion of glucose into lactate and nucleotides; activation of the pentose phosphate pathway, which protects cells from oxidative stress; and increased glucose uptake [32]. Recent studies have revealed that glucose metabolites can regulate the expression of key enzymes involved in glycolysis and lipid synthesis through carbohydrate-responsive element-binding protein under non-insulin signaling pathways, thereby influencing intrahepatic lipid deposition [33]. Furthermore, elevated glycolysis levels have been strongly correlated with poor prognosis in cancer patients [34].

In wild-type p53 mice, pancreatic MDMX deficiency did not affect survival to adulthood; however, it significantly inhibited the proliferation of pancreatic endocrine cells and failed to prevent hyperglycemia, ultimately leading to severe diabetic nephropathy and premature death. Notably, these effects could be rescued by the simultaneous conditional knockdown of p53 [35], highlighting the critical role of MDMX in glucose metabolism regulation in wild-type p53 mice. In this study, metabolomic analysis of liver cancer patients with mutant p53 revealed that MDMX overexpression markedly upregulated the pentose phosphate pathway and aerobic glycolysis. Specifically, MDMX enhanced glucose uptake, lactate secretion, and ATP production, while increasing the expression of RPIA and decreasing PCK1 expression, thereby suppressing gluconeogenesis. These findings demonstrate that MDMX plays a pivotal role in reprogramming glucose metabolism in HCC cells, contributing to glucose metabolic dysregulation in mutant p53 mice.

The specific mechanism by which MDMX regulates the growth and proliferation of HCC cells through FOXO1 modulation and metabolic reprogramming was comprehensively elucidated in Fig. 8D. Bioinformatics analysis revealed that elevated MDMX expression is significantly correlated with poor patient survival in HCC tissues. Functionally, MDMX was demonstrated to enhance tumor cell growth, proliferation, and clonogenic potential in liver cancer cells. Mechanistically, MDMX anchors 14-3-3γ in the cytoplasm and inhibits degradation, thereby downregulating FOXO1. This leads to the upregulation of RPIA and downregulation of PCK1, resulting in increased glucose uptake, lactate secretion, and ATP production. These metabolic alterations drive aerobic glycolysis and glucose metabolic reprogramming in HCC cells. The findings were further validated using a conditional MDMX overexpression transgenic mouse model, which confirmed that MDMX modulates FOXO1 expression and regulates glucose metabolism reprogramming in vivo. These results significantly advance our understanding of the oncogenic role of MDMX in tumorigenesis, provide novel targets for the development of anti-tumor therapeutics, and offer new insights for the diagnosis and treatment of liver cancer.

## Materials and Methods

### Cell lines and culture

Huh7 (p53Y220C), Hep3B (p53 null), HEK293T, PLC/PRF/5 (p53R249S), and H1299 (p53 null) cell lines were purchased from National Collection of Authenticated Cell Cultures (Shanghai, China) and were authenticated by STR profiling. These cell lines were cultivated in high-glucose DMEM medium supplemented with 10% Fetal Bovine Serum. All cells were fostered at 37 °C with 5% CO_2_. No mycoplasma contamination was detected.

### Cell proliferation and colony-formation assay

Cell Counting Kit-8 (CCK-8) assay was performed for testing the speed of cell growth. 8000 cells per well were seeded at 96-well plate in complete DMEM and treated with various concentrations of AS1842856 (HY-100596, MedChemExpress, China) for 24 h. Cells were incubated for 1 h in CO_2_ incubator after adding 10% CCK-8 reagent per well. Then, the optical density (OD) value at 450 nm was measured to assess cell viability.

Colony-formation assay was performed to detect the ability of a single cell to form colonies with different treatments. 3000 cells per well were seeded at 6-well plate in 2 ml complete DMEM or low-glucose DMEM medium with 0.625 μM AS1842856 or 6 mM 2-Deoxy-D-glucose (HY-13966, MedChemExpress, China). After 14 days culture, cells were fixed with methanol, stained with 0.5% crystal violet for 5 min and washed with tap water. Colony numbers were counted with ImageJ.

### Protein extraction and Western blot assay

At approximately 90–95% confluency, cells were washed with cold PBS for 3 times and lysed with RIPA lysis buffer with protease inhibitor (P10045, Beyotime, China) and protein concentrations were measured with the BCA Protein Assay Kit (P0012, Beyotime, China). Equal amounts of protein were separated by SDS-PAGE and transferred onto 0.2 µm PVDF membranes (P2938, Sigma-Aldrich, Germany), then blocked with 5% non-fat milk and incubated with the following primary antibodies overnight at 4 °C: PCK1 (1:1000, 16754-1-AP, Proteintech, China), RPIA (1:1000, 13010-1-AP, Proteintech, China), FOXO1 (1:3000, 18592-1-AP, Proteintech, China), MDMX (1:5000 or 1:1000, A300-287A, Bethyl Laboratories, USA), 14-3-3 family sampler kit (1:1000, #9769, Cell signaling technology, USA), 14-3-3 Sigma (1:1000, 10622-1-AP, Proteintech, China) and β-actin (1:5000, 23660-1-AP, Proteintech, China).

### RNA extraction and RT-qPCR

RNA was extracted from cells with TRIzol reagent (Invitrogen). 1000 ng RNA was reversely transcribed into cDNA with Prime Script RT Reagent Kit (TaKaRa). RT-qPCR was amplified with the SYBR green Master Mix (UElandy). The raw data were normalized to the house-keeping gene GAPDH. Specific primers for real-time PCR are shown in Supplementary Table 1.

### Lentivirus production

The pLKO.1 vector was digested with the restriction enzymes AgeI-HF and EcoRI-HF to generate compatible ends for shRNA insertion. The corresponding shRNA oligonucleotides were annealed and ligated into the digested pLKO.1 vector. Relevant shRNA sequences are shown in Supplementary Table 2. After transformation, plasmids were isolated and validated by sequencing. 293 T cells were co-transfected with 2 μg target gene plasmid construct and helper plasmids (0.5 μg pMD2.G and 1.5 μg psPAX2). The viral supernatant was collected 48 h post-transfection and filtered through a 0.45 μm filter to remove cellular debris. Target cells were infected with the viral supernatant for 48 h and selected using 2 μg/mL puromycin.

### Immunohistochemistry

Tissue samples were initially fixed utilizing formalin, followed by embedding in paraffin. Subsequently, tissue block was cut and affixed to slides. Antigen retrieval was conducted to expose epitopes that may have been masked during the fixation process. To prevent non-specific binding, a blocking serum was employed. Sections were then incubated with following primary antibody at 4 °C overnight: PCK1 (1:300, 16754-1-AP, Proteintech, China), RPIA (1:100, 13010-1-AP, Proteintech, China), FOXO1 (1:300, 18592-1-AP, Proteintech, China), and MDMX (1:500, A300-287A, Bethyl Laboratories, USA). HRP secondary antibody was applied to interact with the primary antibody for 30 min. Ultimately, DAB staining was conducted until the desired color intensity is reached.

The tissue microarray utilized in this study comprises 48 pairs of cancerous and adjacent normal tissues, which were purchased from Shanghai Weiao Biotech Company, China.

### Co-immunoprecipitation

Cells were lysed in lysis buffer containing protease inhibitors, followed by incubation with a specific antibody and protein A/G beads at 4 °C overnight. Beads were washed for three times with lysis buffer and eluted with 1 × SDS loading buffer. The eluted proteins were detected via Western blot to identify interacting partners.

### LC–MS/MS

In HEK 293 T cells, Myc-tagged MDMX was overexpressed, followed by the extraction of total protein. The protein extract was then incubated overnight at 4 °C with magnetic beads that are conjugated to Myc antibody. After washed three times with lysis buffer, the precipitated protein mixtures are separated using SDS-PAGE and further analyzed through mass spectrometry.

### Cycloheximide chase assay

Cells were seeded into 12-well plates and treated with 10 µM CHX (C7698, Sigma-Aldrich, Germany) to inhibit protein synthesis. After incubation for the indicated time, cells were lysed for Western blot analysis. Band intensities were quantified using GraphPad to assess the rate of protein degradation in the presence of CHX.

### Untargeted metabolomics

After transfection with MDMX or Vector for 48 h, the Huh7 cells were washed with ice-cold PBS. Cellular metabolism was immediately quenched by pre-chilled methanol (−80 °C) for 30 s to prevent metabolic alterations. Methanol/water/chloroform ternary solvent system was conducted to extract metabolite. Ice-cold 80% methanol was added to cell pellets at 1:5 ratio. After vortex mixing for 30 s, samples were treated with pulsed ultrasonication on ice (30% amplitude, 3 cycles of 10 s pulses with 20 s intervals). Subsequent centrifugation at 15,000 × *g* for 15 min at 4 °C yielded the polar metabolite-containing supernatant. Further analysis was conducted through mass spectrometry.

### OCR and ECAR assay

1 × 10^4^ cells were seeded into a Seahorse XFp cell culture microplate. For OCR measurement, oligomycin, FCCP, and rotenone were sequentially injected at designated time points. For ECAR detection, glucose, oligomycin, and 2-DG were added sequentially at specified time intervals. Finally, data were analyzed using the Seahorse XFp Wave software, and the results were normalized based on the same protein concentration.

### Measurement of Glucose consumption, ATP, and lactate levels

Cell or tissue samples were collected for glucose consumptions, ATP, and lactate levels determination according to the manufacturer’s protocol. The Glucose, ATP and lactate levels in each group were measured with the Glucose Assay Kit (F006-1-1, Nanjing Jiancheng Bioengineering Institute, China), ATP Assay Kit (A095-1-1, Nanjing Jiancheng Bioengineering Institute, China), and Lactic acid (LA) content detection kit (BC2235, Solarbio, China).

### Chromatin immunoprecipitation (ChIP)

1 × 10^7^ cells were treated with 1% formaldehyde for 10 min at room temperature to crosslink protein to DNA, followed by glycine quenching. Cells were lysed in RIPA buffer with protease inhibitors. Lysates were sonicated to shear chromatin to an average length of about 500–1000 bp, then spun down at 12,000 × *g* for 10 min to remove debris. Supernatant was incubated with 3 μg FOXO1 antibody at 4 °C overnight, then captured with protein A/G beads for 4 h at 4 °C. After washed with low-salt, high-salt, LiCl, and TE buffers, beads were eluted in elution buffer. The samples were de-crosslinked at 65 °C for 18 h, then treated with 10 mg/ml RNaseA at 37 °C for 1 h and proteinase K at 45 °C for 2 h. DNA was extracted using phenol-chloroform, then analyzed by real-time PCR. The primers are shown in Supplementary Table 3.

### In vivo assay

Male BALB/c nude mice, aged 4 to 5 weeks, were purchased from GemPharmatech Co., Ltd. Conditional Mdmx overexpression (Rosa26-CAG-LSL-mMdmx-P2A-zsGreen-polyA) mice were purchased from Shanghai Model Organisms Center Inc. and has been described previously [36]. B6-p53 R172H mice (Strain NO. T007671) were purchased from GemPharmatech Co., Ltd. These mice were maintained in the Animal Experiment Center of Fujian Medical University, operating under specific pathogen-free conditions and provided with a standard diet. All the animal procedures were approved by the Institutional Animal Care and Use Committee of Fujian Medical University (IACUC FJMU 2022-0649, IACUC FJMU 2022-0464).

To create a tumor xenograft model, the nude mice were randomly assigned to two groups: Ctrl, MDMX, with each group comprising five mice. A total of 5 million Huh7 cells were injected subcutaneously into the right axillary region of the mice. Tumor dimensions were assessed every other day following inoculation, alongside monitoring of body weight. The individuals responsible for measuring tumor size and weight were blinded to the group allocations. Upon reaching a tumor volume of 1.5 cm³, the mice were euthanized for subsequent tumor analysis.

Conditional Mdmx overexpression mice were bred with C57BL/6JGpt mice to establish a stable heritable F1 generation positive mouse model. The Mdmx-P2A-zsGreen gene can be specifically expressed in liver tissue after mating with Alb-Cre tool mice. By interbreeding B6-p53 LSL-R172H mice with Alb-Cre mice, mRNA expression of the R172H mutation was detected within liver tissue of the offspring. Conditional Mdmx overexpression mice and B6-p53 LSL-R172H mice were crossed to generate Mdmx^flox/+^; Trp53^R172H/+^ mice.

### Statistical analysis

For comparisons involving two groups with normally distributed data that satisfied the chi-square test, Student’s two-tailed *t*-test were used. In cases of multiple-group comparisons with normally distributed data, one-way ANOVA was applied. All data were expressed as mean ± standard error of the mean (SEM). GraphPad Prism 8.0 was used for statistical analysis and graphical data presentation. *P*-value of less than 0.05 was considered statistically significant.

## Supplementary information

Supplementary Material

Uncropped blot

## Data Availability

The data generated in this study are available upon request from the corresponding author.
