# Supplementary material for: MDMX reprograms glycolysis of hepatocellular carcinoma via 14-3-3γ/FOXO1
Source: Cell Death Discov. 2025 Nov 7;11:509. doi: 10.1038/s41420-025-02804-2 (PMC12595022; doi:10.1038/s41420-025-02804-2)
Supplement: Supplementary file 1 — Supplementary Material [file 41420_2025_2804_MOESM1_ESM.pdf]

Supplementary Material

Supplementary Table 1. Primer sequences for qRT-PCR

MDMX	Human	Forward	5’-GAGCTGAACTCCCTGCTGAT-3’
		Reverse	5’-TTTTCTCCCTGGCGAAGTC-3’
FOXO1	Human	Forward	5’-CAAGGCAGGTTTCTGAGGAG-3’
		Reverse	5’-GACCTGGTCACCATCAGCAT-3’
RPIA	Human	Forward	5’-ACCTGGATCAACACCCAGAG-3’
		Reverse	5’-TAGGCCATTGGGATGACTTC-3’
PCK1	Human	Forward	5’-CTCCAAGCTGGTCATTATCACG-3’
		Reverse	5’-AGTTCGGGCTGTATTTTACAACA-3’
HK2	Human	Forward	5’-AAGGTGATCGAGGAGTTCTACA-3’
		Reverse	5’-ATGCCCCCAACAGAAAAGATG-3’
PKM2	Human	Forward	5’-CTGAAGGCAGTGATGTGGCC-3’
		Reverse	5’-ACCCGGAGGTCCACGTCCTC-3’
AQP9	Human	Forward	5’-CTTAACAATTCACAAGGCACTT-3’
		Reverse	5’-TCTCAGCCAGCTACTGATCTTC-3’

Supplementary Table 2. Lists of shRNA Target Sequence for MDMX and 14-3-3γ.

sh-MDM4-1	AAGAACTACAGAAGACGATAT
sh-MDM4-2	AAAGATTCAGCTGGTTATTAA
sh-14-3-3γ-1	GAGGAACGAAACCTTCTGTTT
sh-14-3-3γ-2	CAGACGGCAATGAGAAGAATT

Supplementary Table 3. Primer sequences for ChIP-qPCR.

PCK1 promoter	Human	Forward	5’-GGGAGTGACACCTCA-3’
		Reverse	5’-GTGTGCCAGTGGCTGC-3’

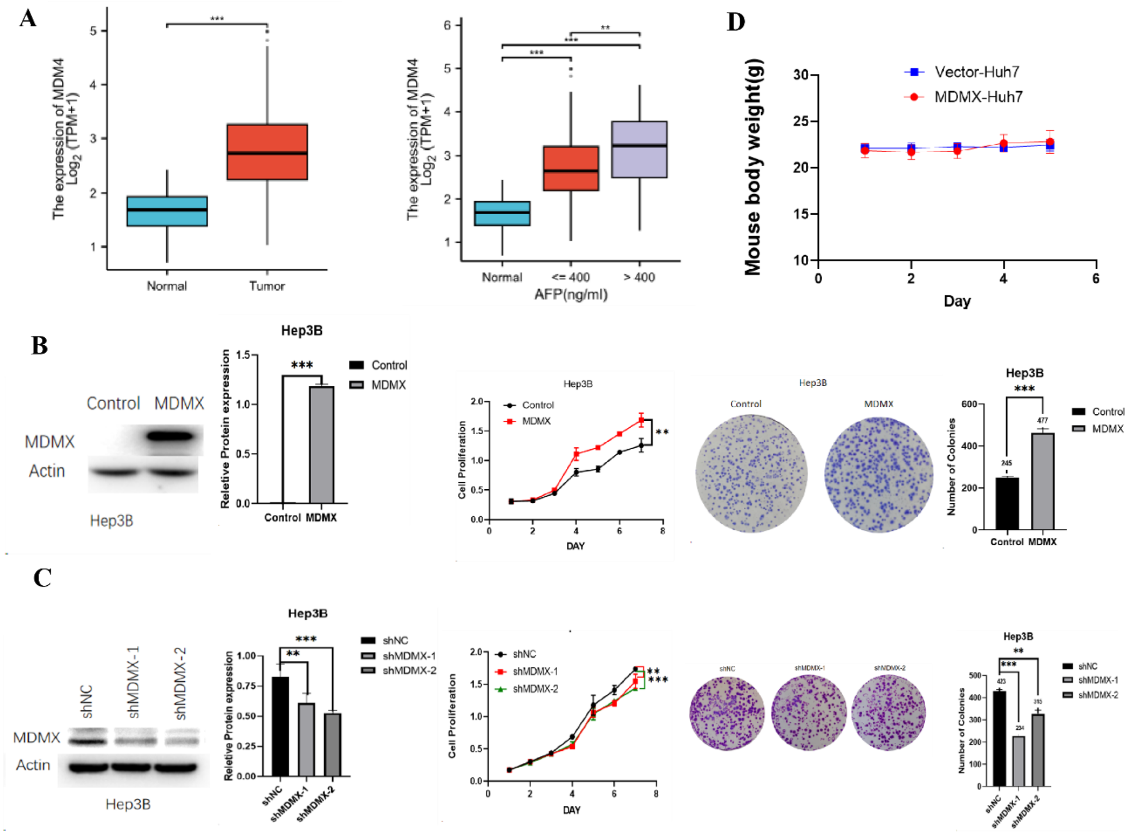

Supplementary Figure S1. MDMX promotes growth of HCC cells harboring mutant p53. (A) The co-relation analysis of MDMX expression and AFP level in HCC. **(B, C)** Western blot confirming MDMX expression in stable overexpression or knockdown cell lines. CCK-8 and colony formation assays indicated the effect of MDMX on cell growth in Hep3B (n=3). **(D)** Huh7 overexpressing MDMX injected subcutaneously in nude mice. Graph showed weights of mice. $^{**}P < 0.01$, $^{***}P < 0.001$.**(B)**T-test and **(A, C)** One-way ANOVA were used for statistical analysis.

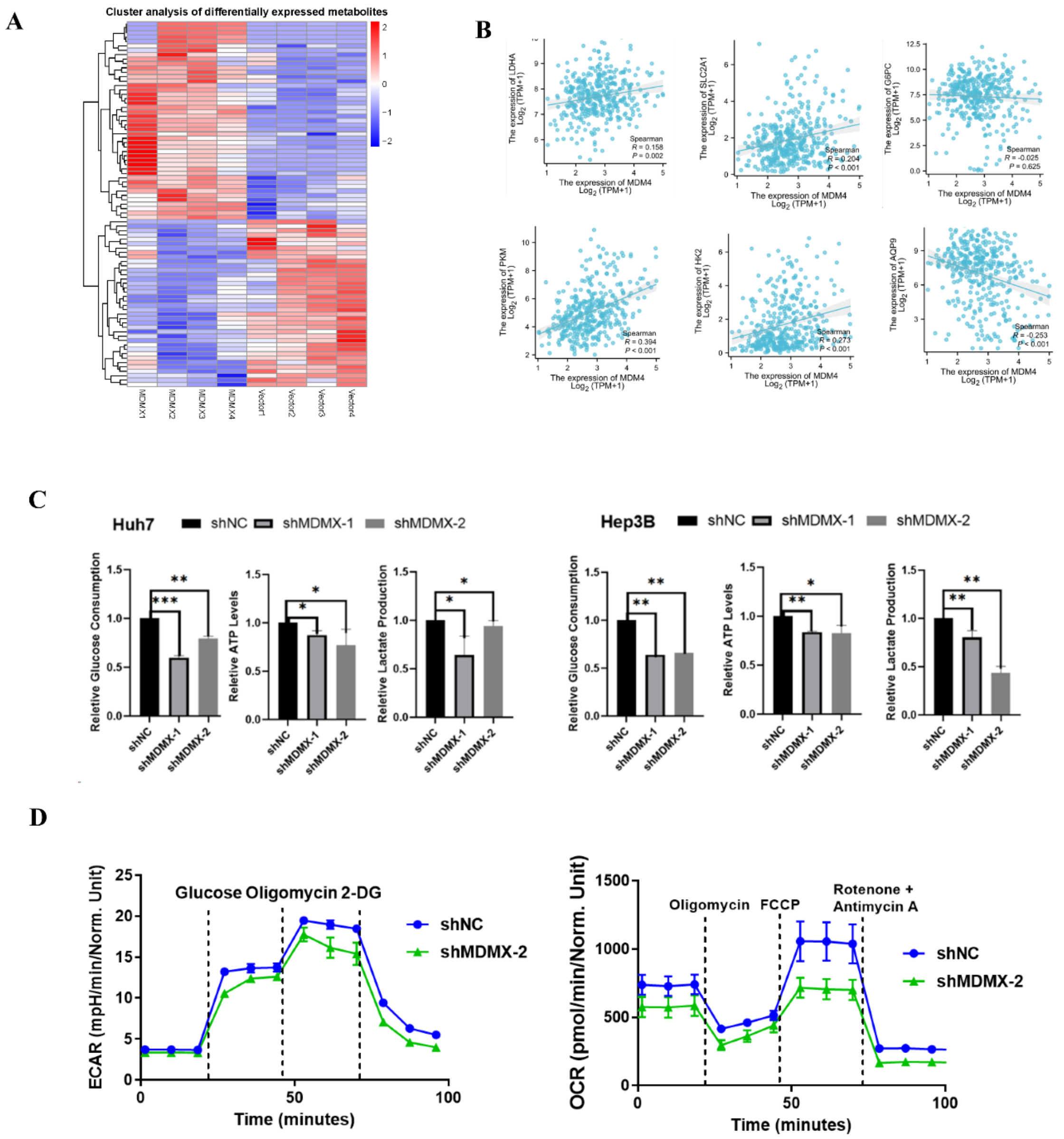

Supplement Figure S2. MDMX promotes glycolysis of HCC cells. (A) Hierarchical clustering analysis diagram of differential metabolites: the horizontal axis represents different experimental groups, and the vertical axis represents the differential metabolites compared in that group. (**B**) The correlation between MDMX and genes in the glucose metabolism pathway: a positive slope indicates a positive correlation, while a negative slope indicates a negative correlation. (**C**) In Huh7 and Hep3B cells knocking down MDMX, the levels of glucose uptake, ATP, and lactate production in the cells were measured. (**D**) In Huh7 and Hep3B cells knocking down MDMX, the oxygen consumption rate and extracellular acidification rate of the cells were measured using Seahorse XFp. *: $P<0.05$; **: $P<0.01$; ***: $P<0.001$. **(C)** One-way ANOVA were used for statistical analysis.

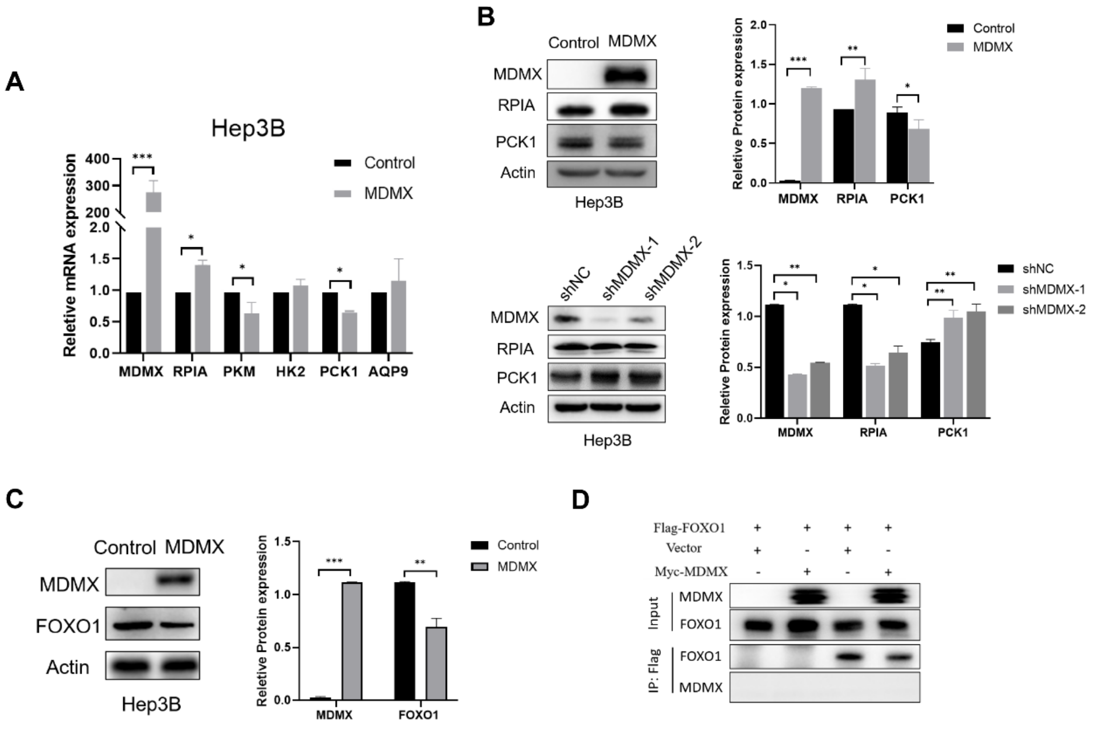

Supplementary Figure S3. MDMX downregulates the protein levels of FOXO1 via interaction with 14-3-3γ. (A) In Hep3B with overexpression of MDMX, mRNA level of RPIA, PKM, HK2, PCK1, AQP9 and G6PC was detected by q-PCR. (**B)** In Hep3B with overexpression or knockdown of MDMX, the protein level of PCK1 and RPIA were measured by Western blot. (**C)** In Huh7 with overexpression of MDMX, the protein level of FOXO1 were measured by Western blot. **(D)** Co-Immunoprecipitation was conducted to explore the interaction between MDMX and FOXO1.

A

1	Accessi	Description	Coverag	# Peptic	# PSMs	# Uniqu	# AAs	MW [kD	calc. pI	Abundan	Abundanc	M-G	M/G
133	P61981	14-3-3 protein gamma OS=Homo sapiens OX=9606 GN=YWHAG PE=1 SV=2	33	8	14	4	247	28.3	4.89	718219.28	8577227.88	7859008.6	11.9423526
141	Q04917	14-3-3 protein eta OS=Homo sapiens OX=9606 GN=YWHAH PE=1 SV=4	28	7	12	3	246	28.2	4.84	1510575.7	14411840.5	12901264.8	9.54062752
152	P31946	14-3-3 protein beta/alpha OS=Homo sapiens OX=9606 GN=YWHAB PE=1 SV=3	48	11	20	5	246	28.1	4.83	4428425.8	27295619.8	22867194	6.16372973
159	P27348	14-3-3 protein theta OS=Homo sapiens OX=9606 GN=YWHAQ PE=1 SV=1	53	15	29	9	245	27.7	4.78	81080383	432174607	351094223	5.33019935
166	P63104	14-3-3 protein zeta/delta OS=Homo sapiens OX=9606 GN=YWHAZ PE=1 SV=1	53	14	24	9	245	27.7	4.79	21697707	97972012.5	76274305.1	4.51531633
174	P62258	14-3-3 protein epsilon OS=Homo sapiens OX=9606 GN=YWHAE PE=1 SV=1	45	11	19	8	255	29.2	4.74	20589754	83279769.8	62690015.8	4.04471903

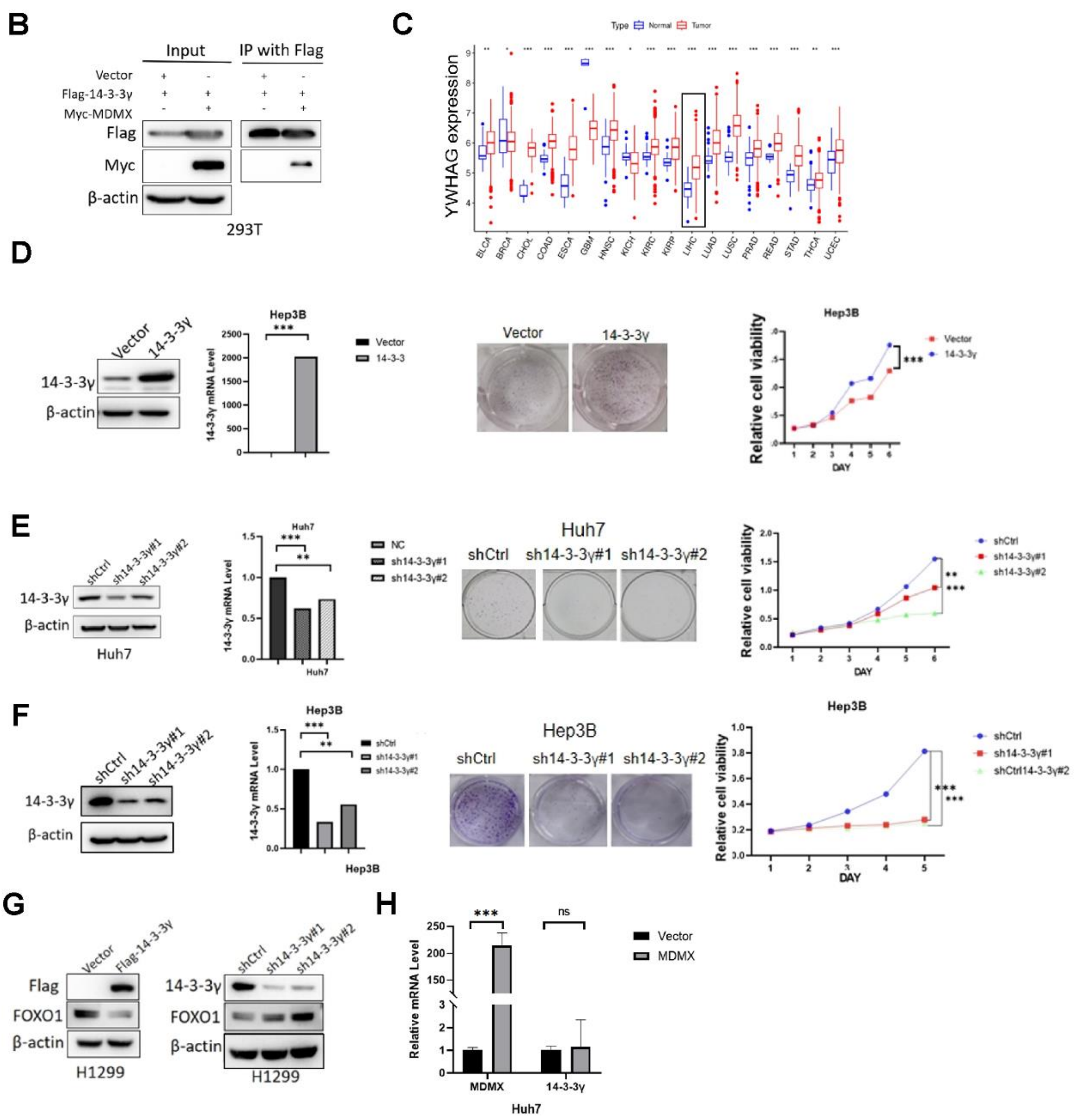

Supplementary Figure S4. 14-3-3γ promotes the progression of HCC through interaction with FOXO1. **(A)** The result of mass spectrometry showed that 14-3-3 protein could bind MDMX. (**B)** Co-Immunoprecipitation verified the interaction between MDMX and 14-3-3γ via Flag antibody in 293T cells. **(C)** TCGA database analysis showing 14-3-3γ expression levels in various cancer. **(D, E, F)** Western blot and RT-qPCR confirming 14-3-3γ expression in stable overexpression or knockdown cell lines. CCK-8 and colony formation assays indicated the effect of 14-3-3γ on cell growth in Huh7 or Hep3B (n=3). **(G)** In H1299 cells with overexpression or knockdown of 14-3-3γ, the protein level of FOXO1 were measured by Western blot. **(H)** q-PCR was used to detect 14-3-3γ level after overexpression of MDMX in Huh7 cells *P < 0.05, **P < 0.01, ***P < 0.001. **(D, H)** T test and **(E, F)** One-way ANOVA was used for statistical analysis.

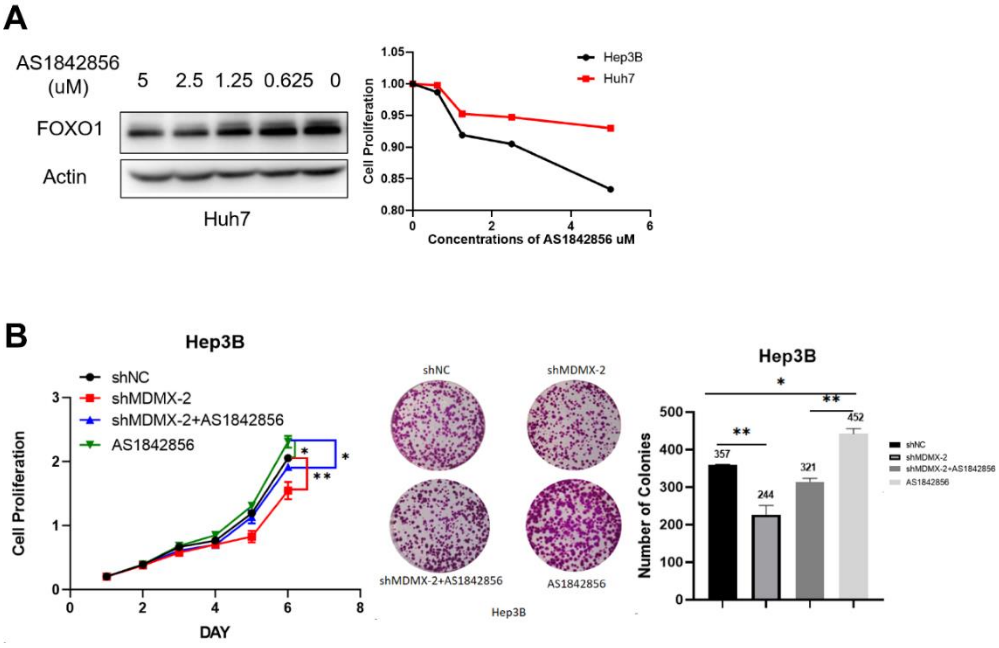

Supplementary Figure S5. FOXO1 inhibitor AS1842856 promotes cell proliferation. (A) The Huh7 cells were treated with different concentrations of AS1842856 for 24 hours, and the protein expression levels of FOXO1 and cell growth were measured by Western blot and cell viability tests. (**B**) Colony formation and cell proliferation assays were conducted in Hep3B cells with MDMX knockdown and AS1842856 treatment. **P*<0.05, ***P*<0.01. **(B)** One-way ANOVA was used for statistical analysis.

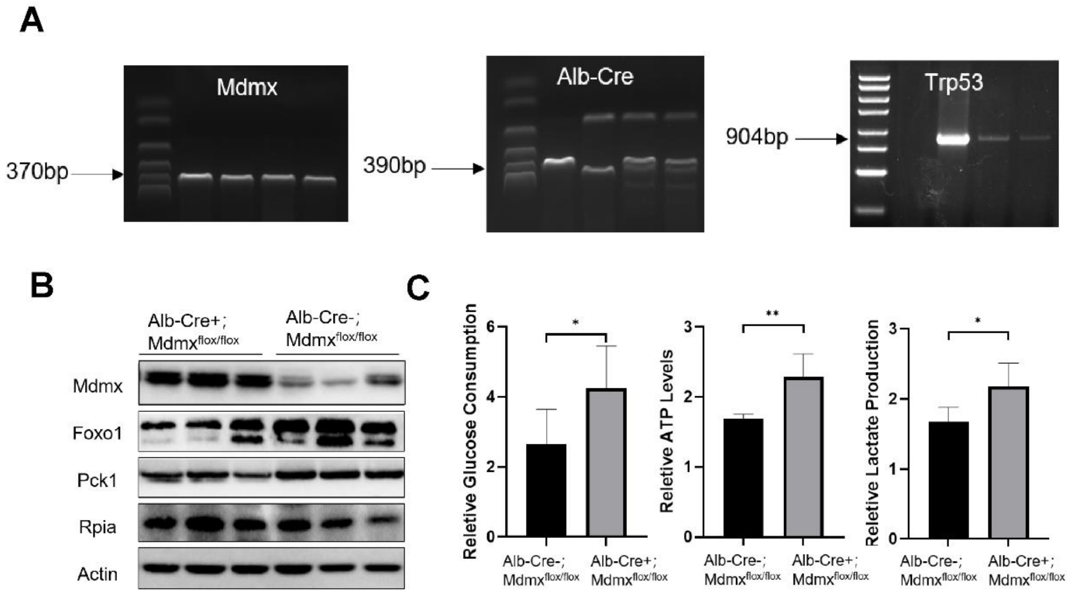

Supplementary Figure S6. MDMX promotes glycolysis through inhibiting FOXO1 in transgenic mice. (A) Genotyping of tissue-specific expression mice. **(B)** Expression levels of Foxo1, Pck1 and Rpia were detected in liver tissues from mice. **(C)** The levels of glucose uptake, ATP, and lactate in the liver tissues were measured. **P*<0.05, ***P*<0.01. **(C) T-test** were used for statistical analysis.
