# Supplementary material for: MDMX reprograms glycolysis of hepatocellular carcinoma via 14-3-3γ/FOXO1
Source: Cell Death Discov. 2025 Nov 7;11:509. doi: 10.1038/s41420-025-02804-2 (PMC12595022; doi:10.1038/s41420-025-02804-2)

Fig. 1E

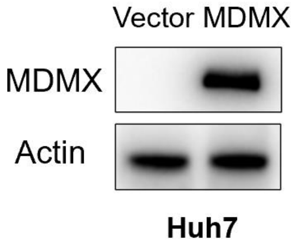

Fig. 1E MDMX

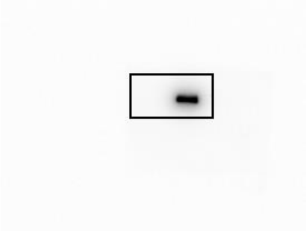

Fig. 1E Actin

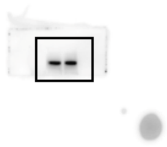

Fig. 1H

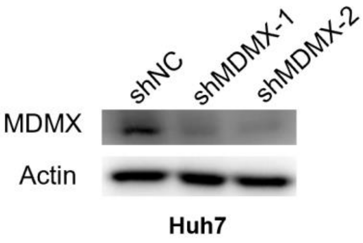

Fig. 1H MDMX

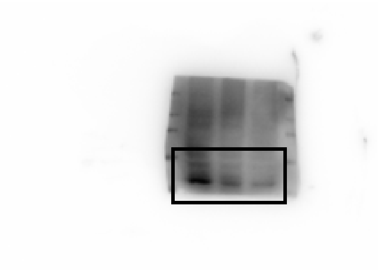

Fig. 1H Actin

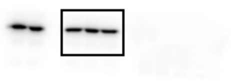

Fig. 3B MDMX OE

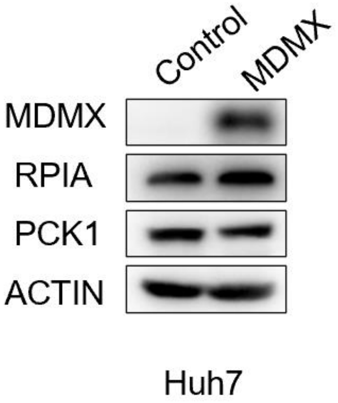

Fig. 3B MDMX OE-MDMX

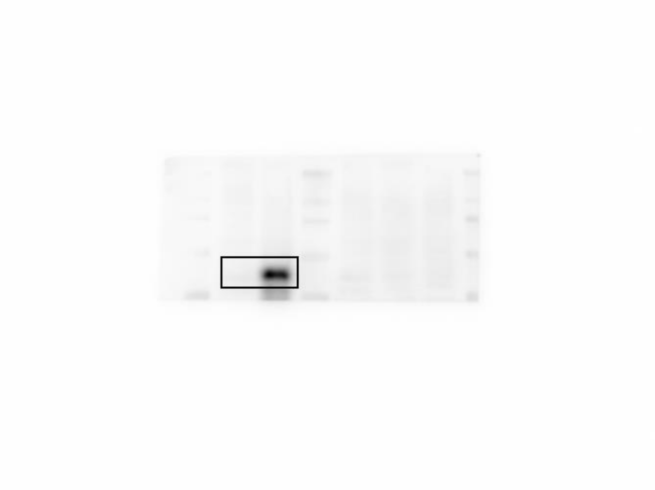

Fig. 3B MDMX OE-RPIA

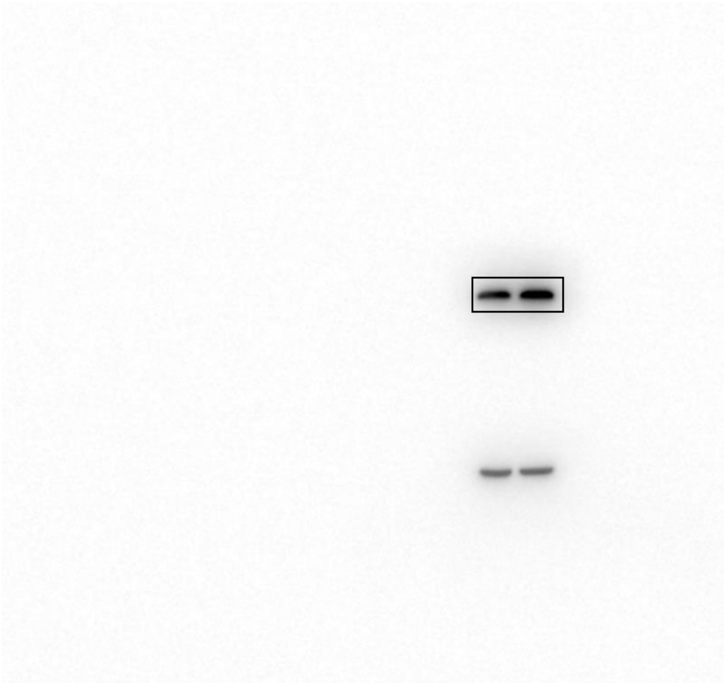

Fig. 3B MDMX OE-PCK1

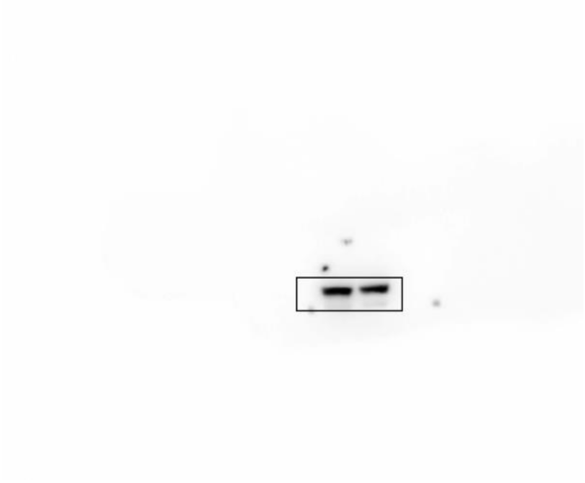

Fig. 3B MDMX OE-Actin

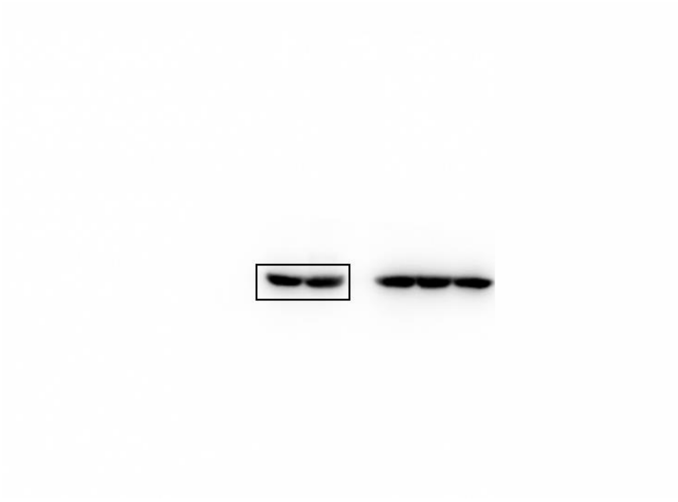

Fig. 3B shMDMX

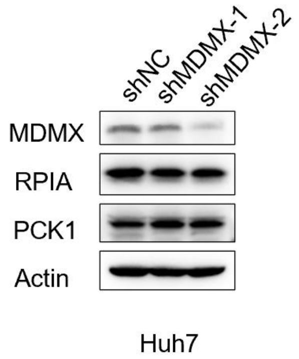

Fig. 3B shMDMX-MDMX

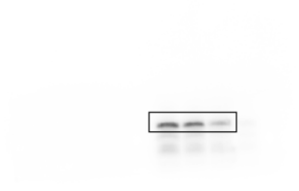

Fig. 3B shMDMX-RPIA

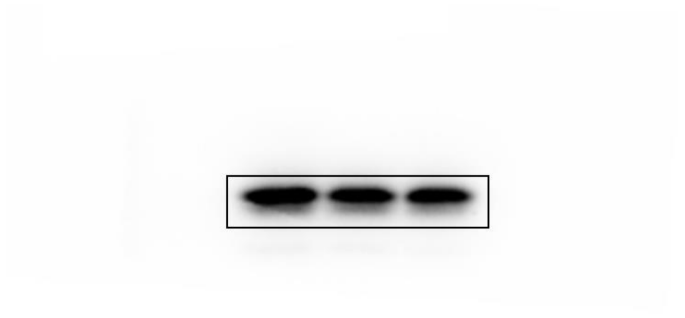

Fig. 3B shMDMX-PCK1

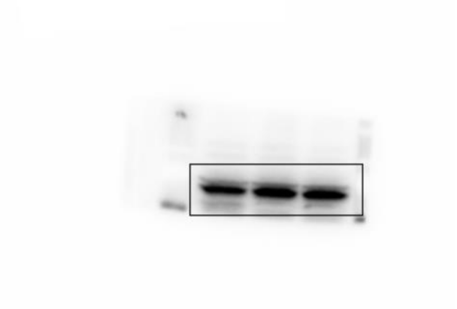

Fig. 3B shMDMX-Actin

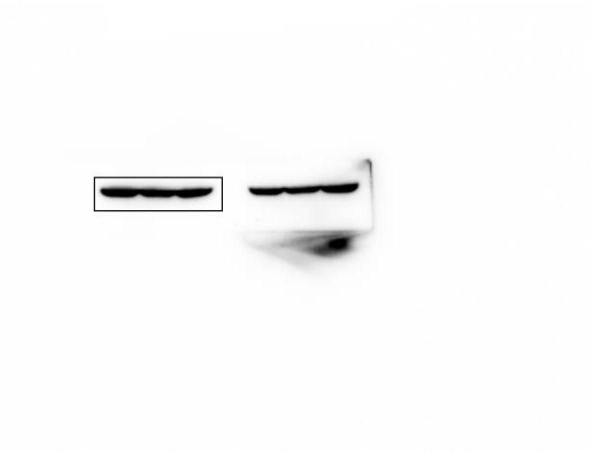

Fig. 3D MDMX OE

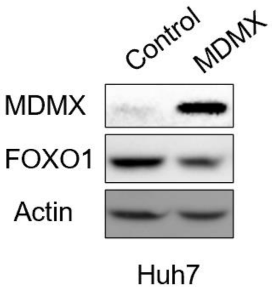

Fig. 3D MDMX OE-MDMX

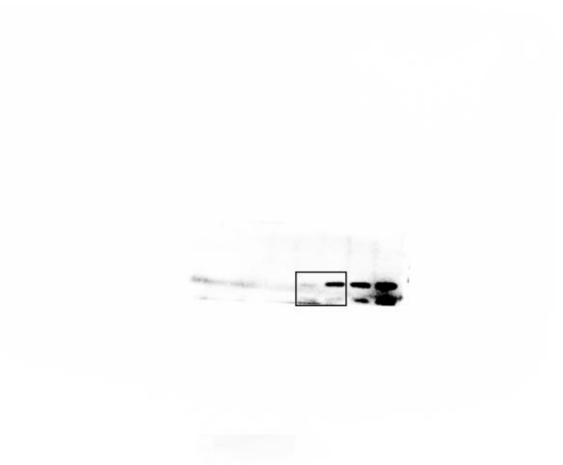

Fig. 3D MDMX OE-FOXO1

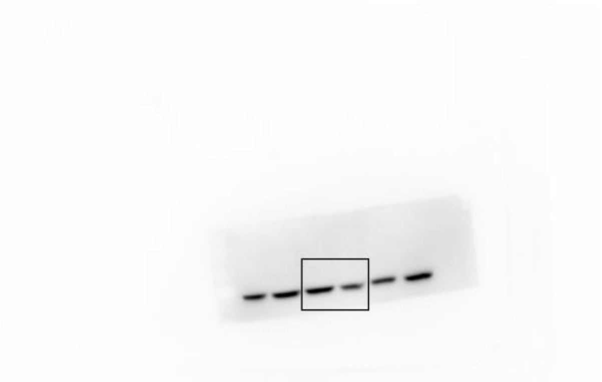

Fig. 3D MDMX OE-Actin

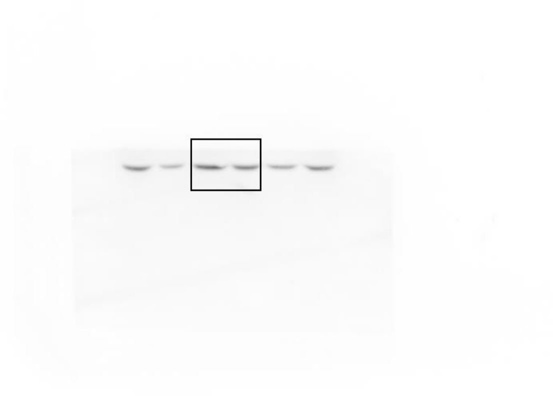

Fig. 3D shMDMX

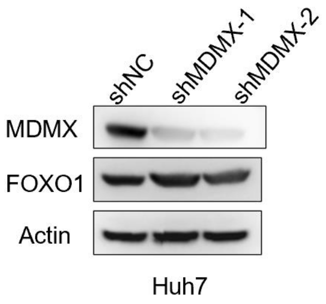

Fig. 3D shMDMX-MDMX

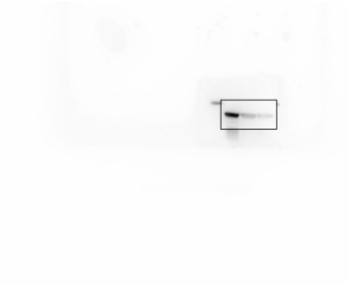

Fig. 3D shMDMX-FOXO1

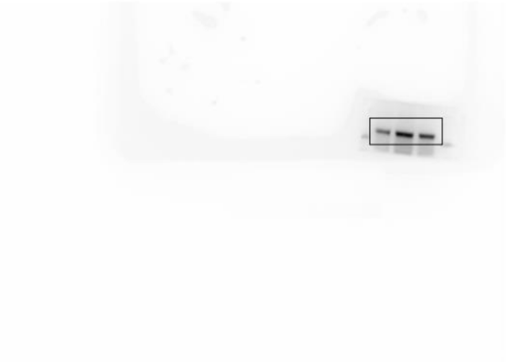

Fig. 3D shMDMX-Actin

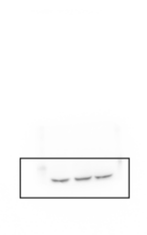

Fig. 4C

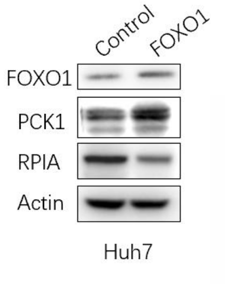

Fig. 4C-FOXO1

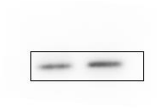

Fig. 4C-PCK1

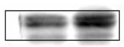

Fig. 4C-RPIA

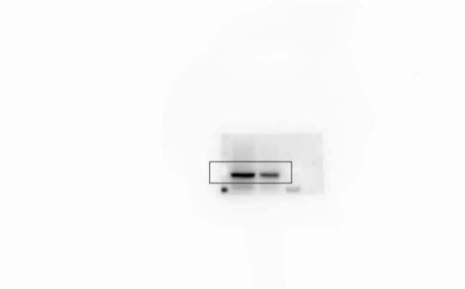

Fig. 4C-Actin

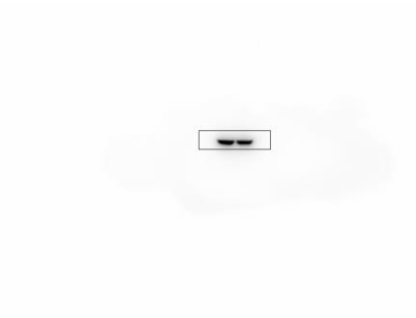

Fig. 4F

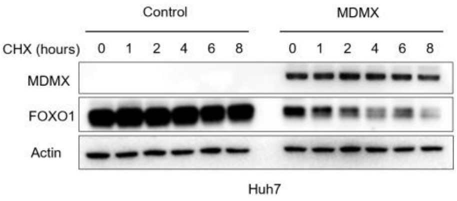

Fig. 4F MDMX

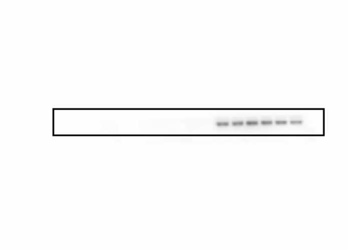

Fig. 4F FOXO1

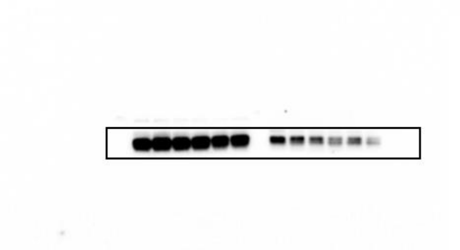

Fig. 4F Actin

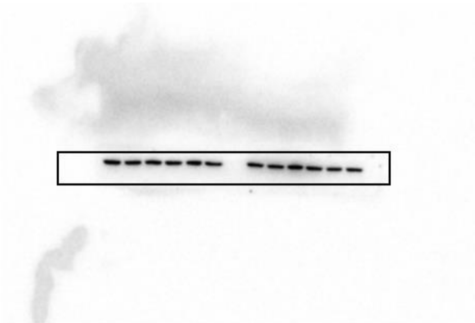

Fig. 5B input

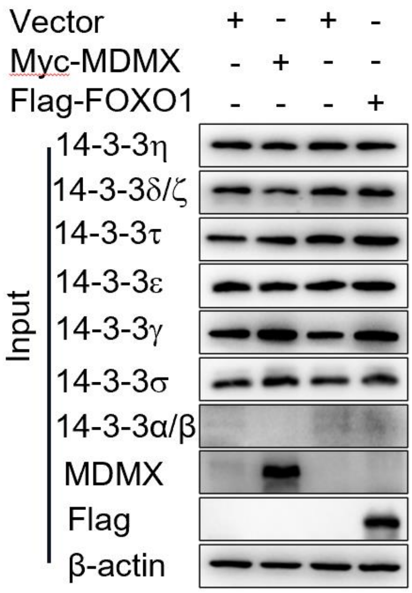

Fig. 5B input 14-3-3η

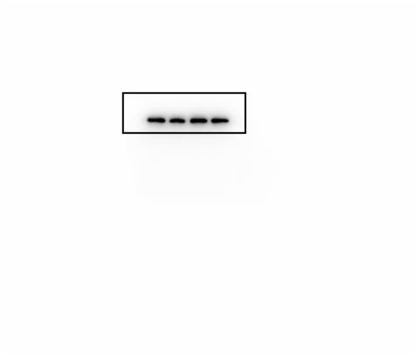

Fig. 5B input 14-3-3δ/ζ

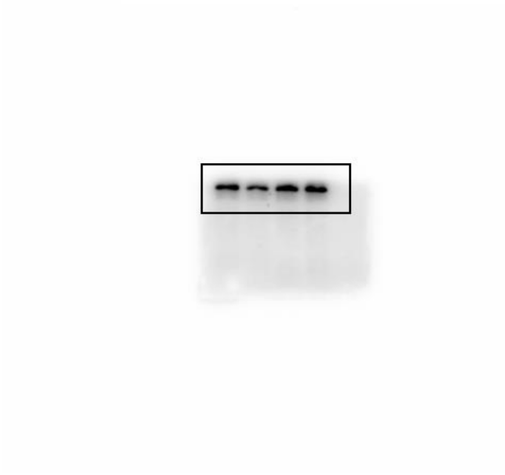

Fig. 5B input 14-3-3τ

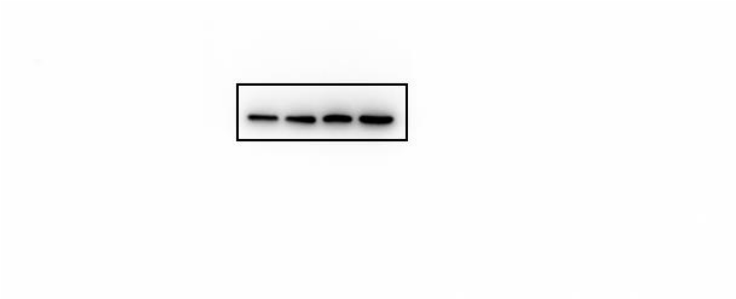

Fig. 5B input 14-3-3ε

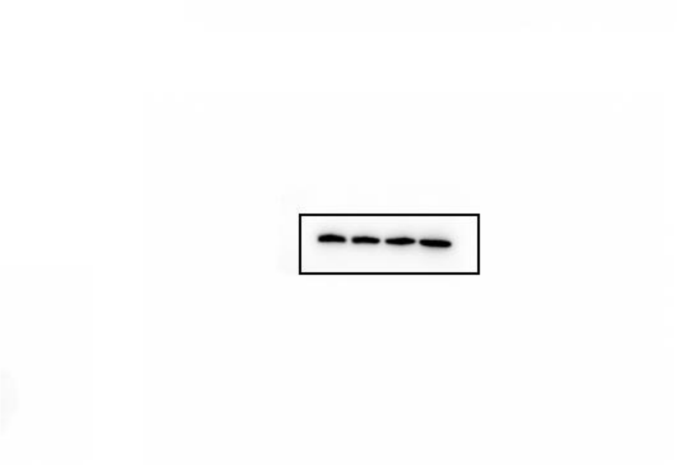

Fig. 5B input 14-3-3γ

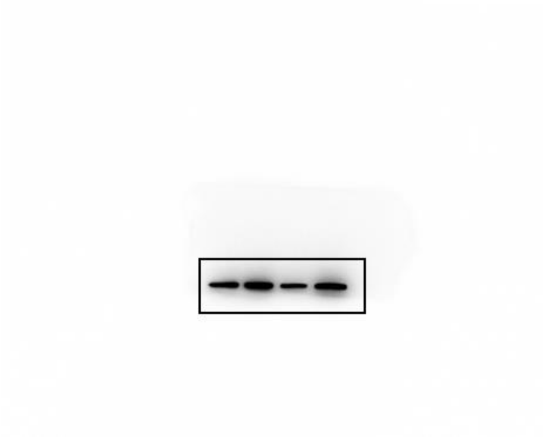

Fig. 5B input 14-3-3σ

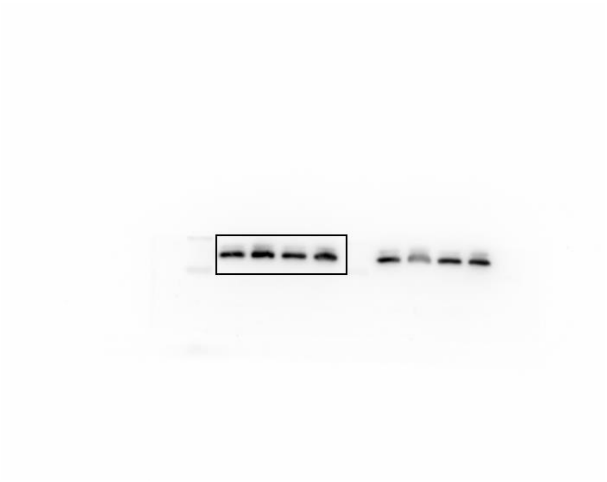

Fig. 5B input 14-3-3α/β

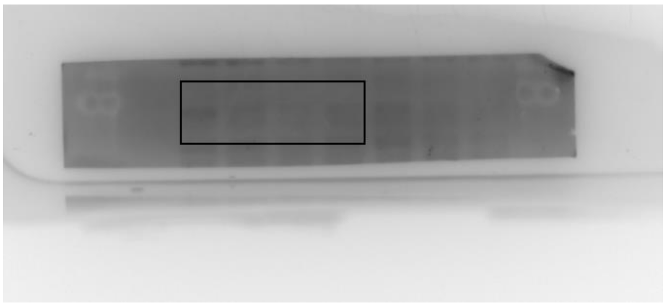

Fig. 5B input MDMX

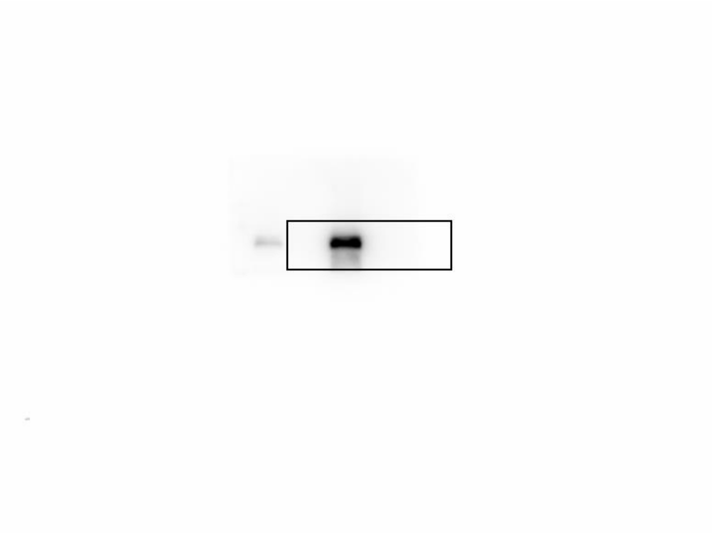

Fig. 5B input Flag

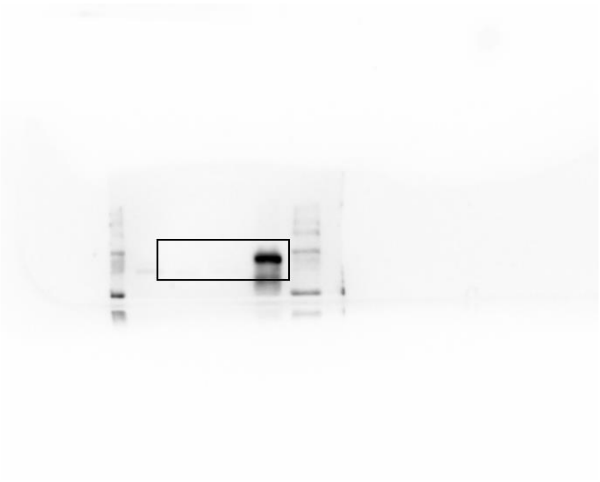

Fig. 5B input β-actin

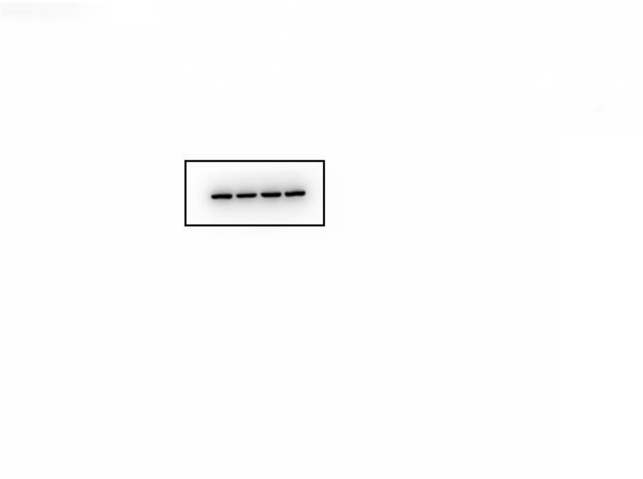

Fig. 5B IP: Myc

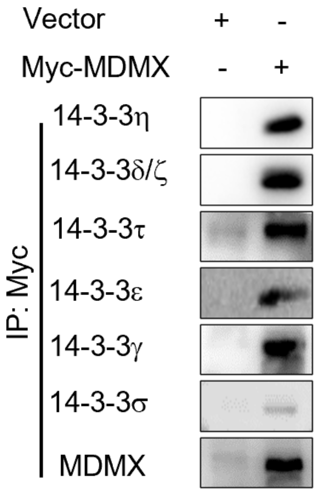

Fig. 5B IP: Myc 14-3-3η

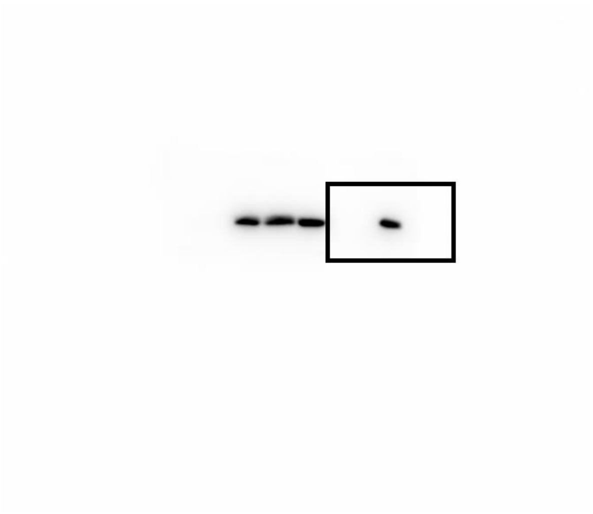

Fig. 5B IP: Myc 14-3-3δ/ζ

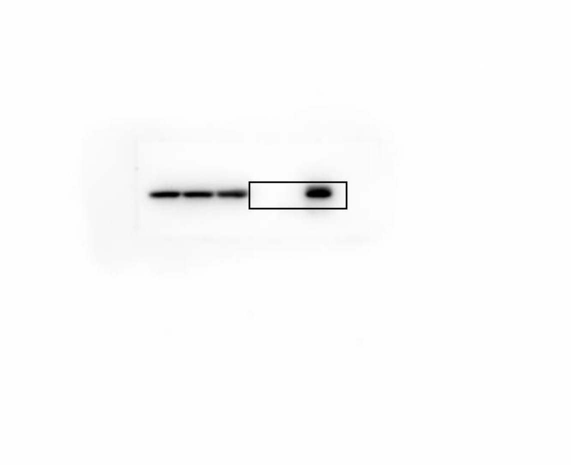

Fig. 5B IP: Myc 14-3-3τ

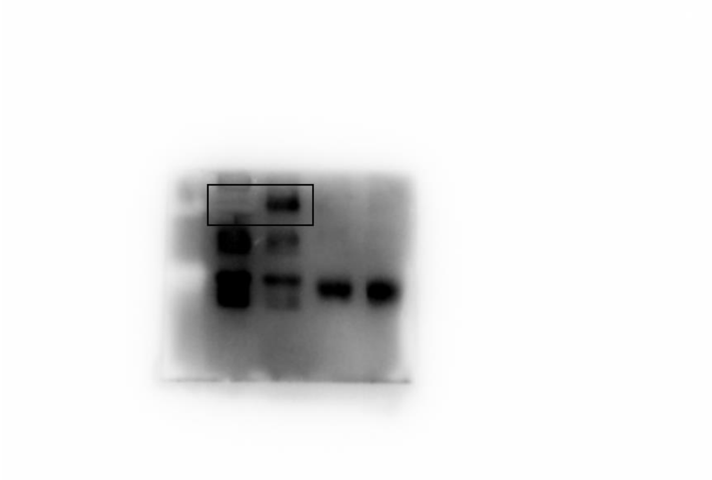

Fig. 5B IP: Myc 14-3-3ε

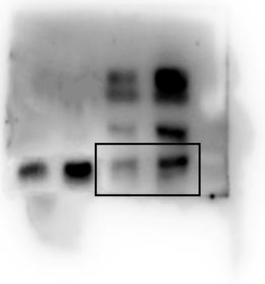

Fig. 5B IP: Myc 14-3-3γ

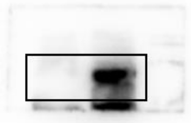

Fig. 5B IP: Myc 14-3-3σ

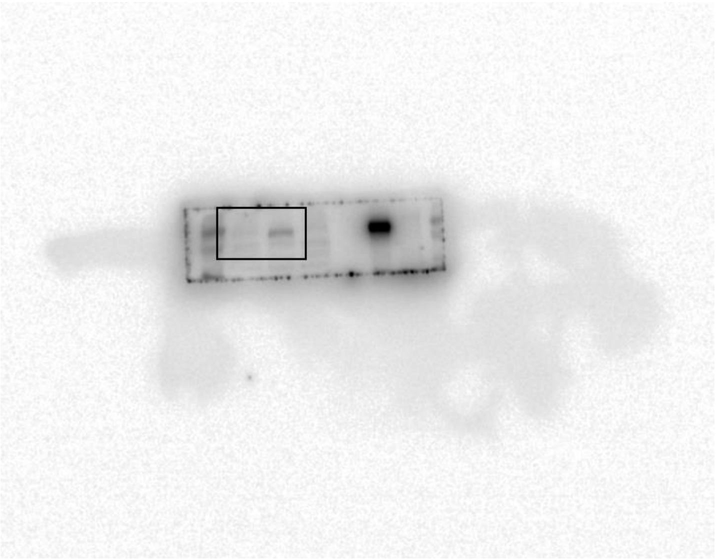

Fig. 5B IP: Myc MDMX

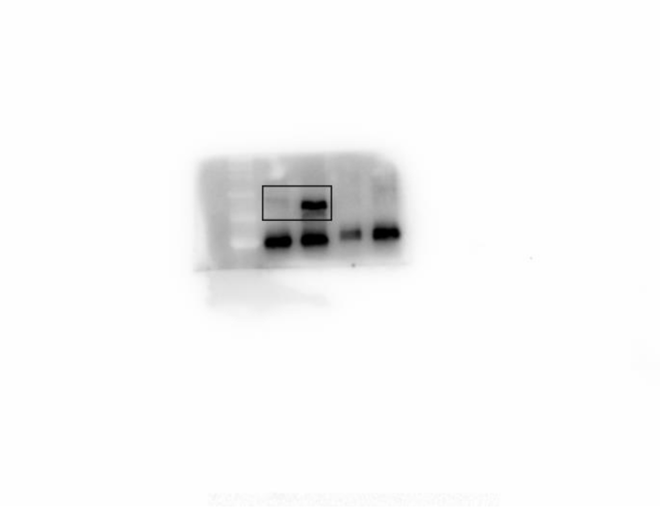

Fig. 5B IP: Flag

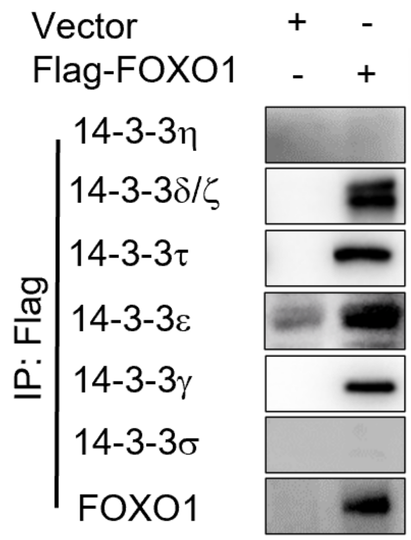

Fig. 5B IP: Flag 14-3-3η

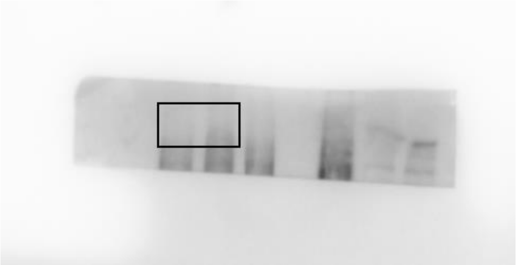

Fig. 5B IP: Flag 14-3-3δ/ζ

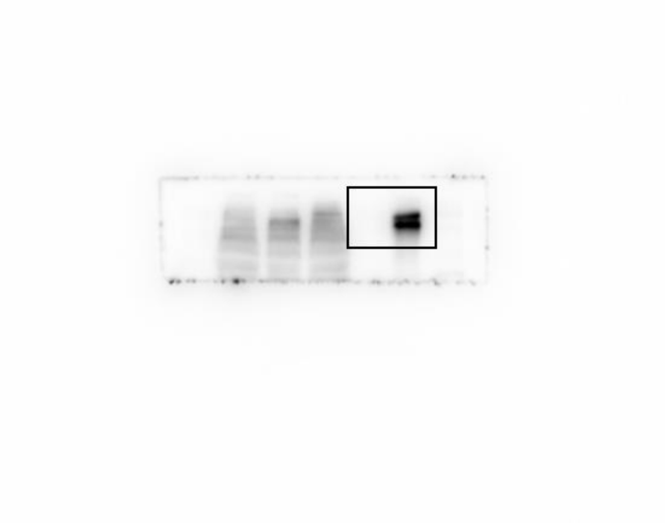

Fig. 5B IP: Flag 14-3-3τ

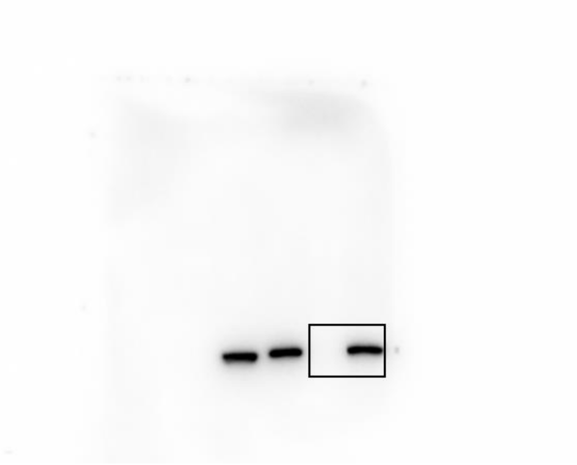

Fig. 5B IP: Flag 14-3-3ε

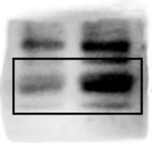

Fig. 5B IP: Flag 14-3-3γ

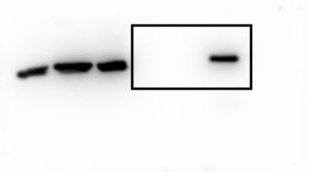

Fig. 5B IP: Flag 14-3-3σ

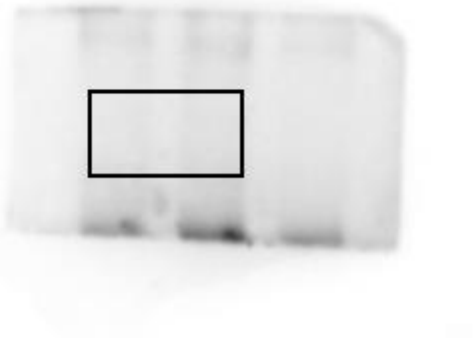

Fig. 5B IP: Flag FOXO1

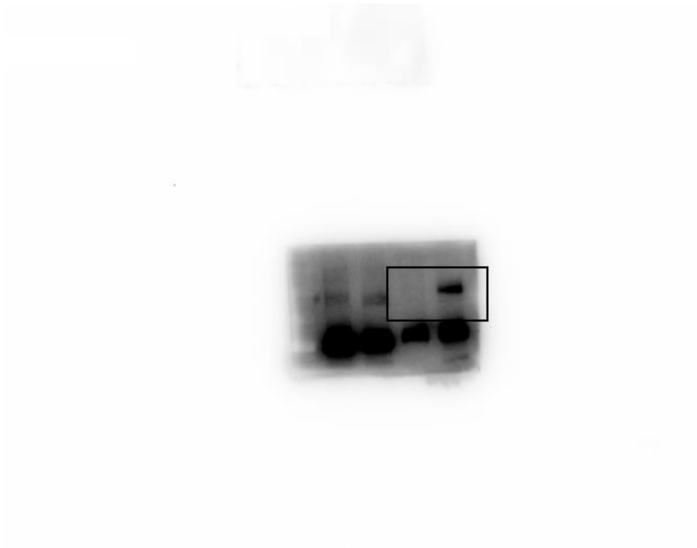

Fig. 5C Input

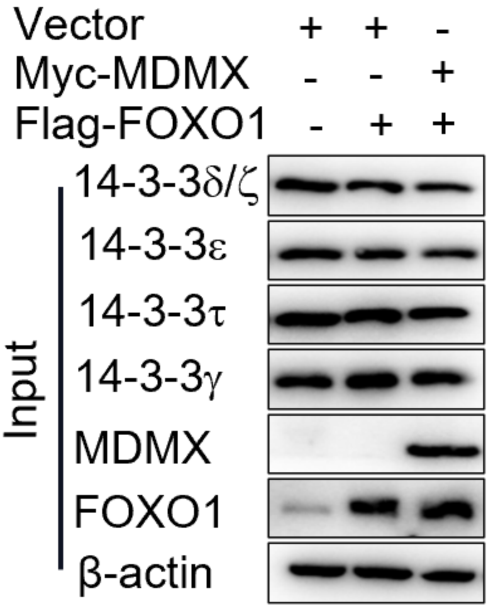

Fig. 5C Input 14-3-3δ/ζ

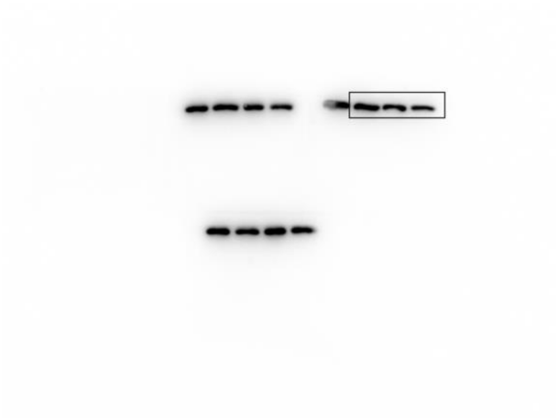

Fig. 5C Input 14-3-3ε

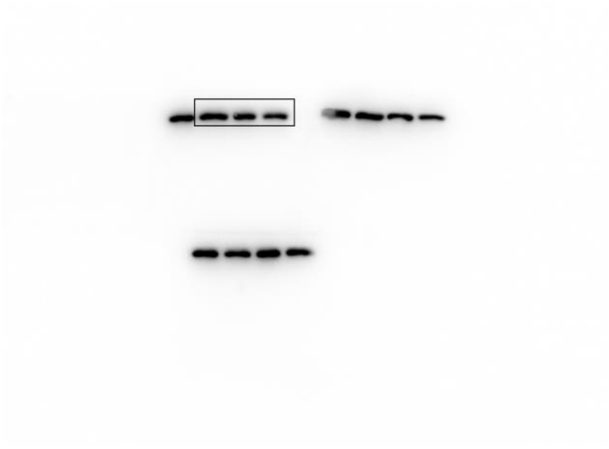

Fig. 5C Input 14-3-3τ

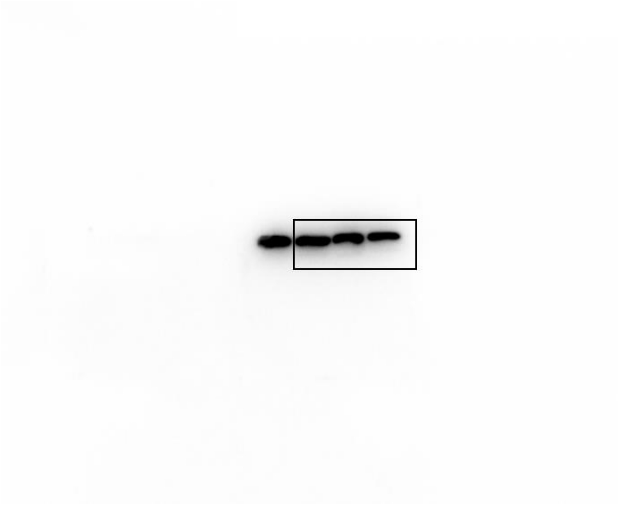

Fig. 5C Input 14-3-3γ

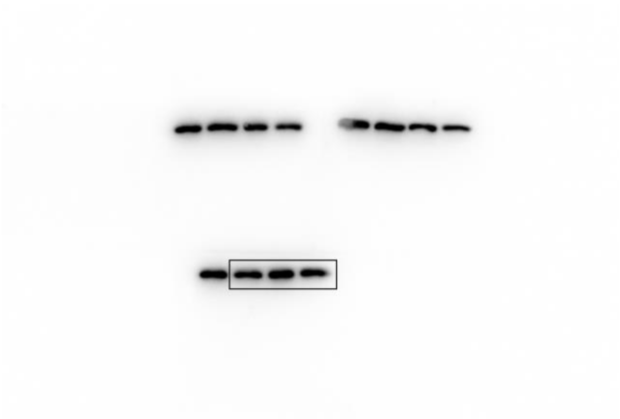

Fig. 5C Input MDMX

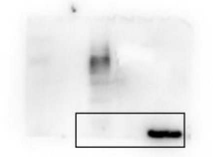

Fig. 5C Input FOXO1

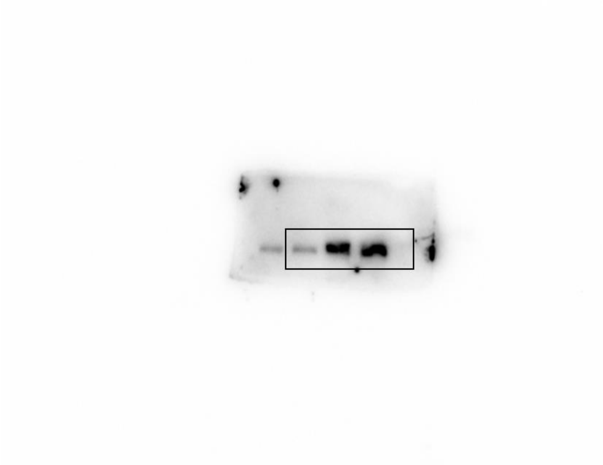

Fig. 5C Input β-actin

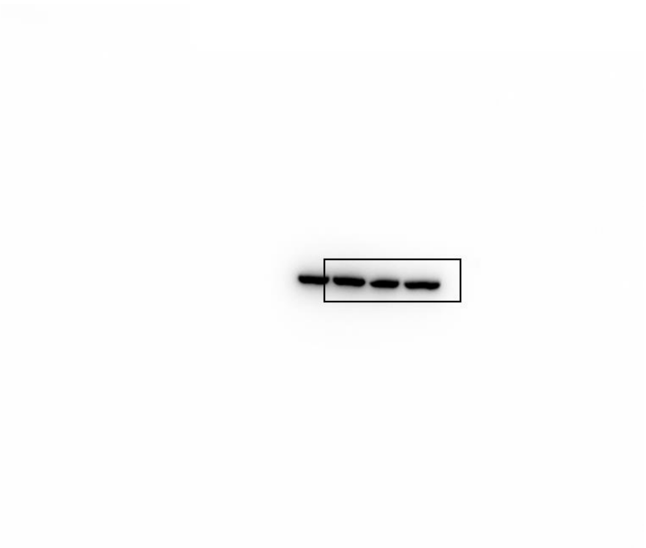

Fig. 5B IP:Flag

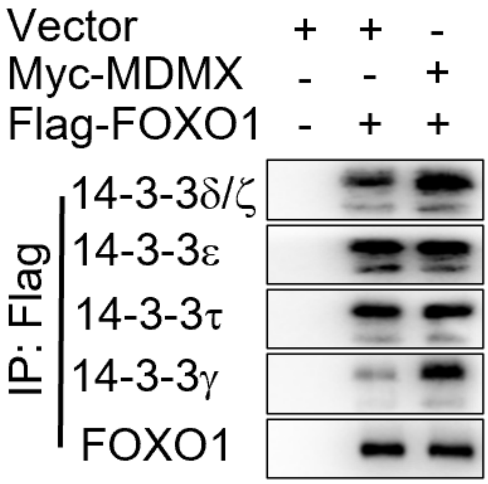

Fig. 5B IP:Falg 14-3-3δ/ζ

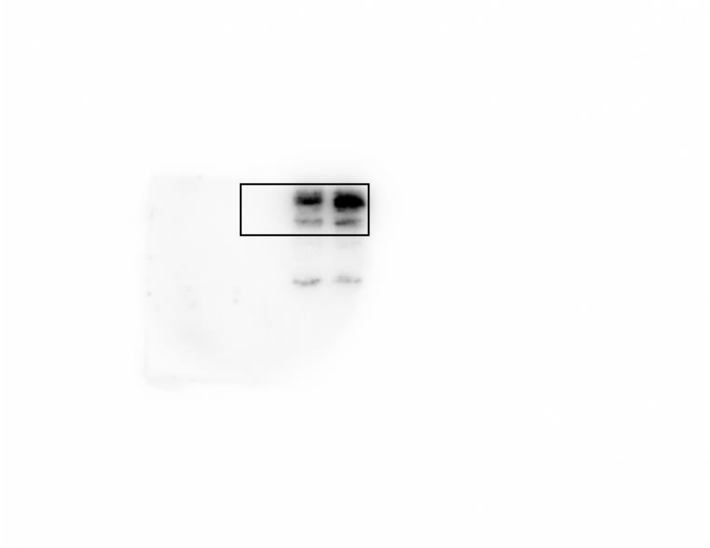

Fig. 5B IP: Falg 14-3-3ε

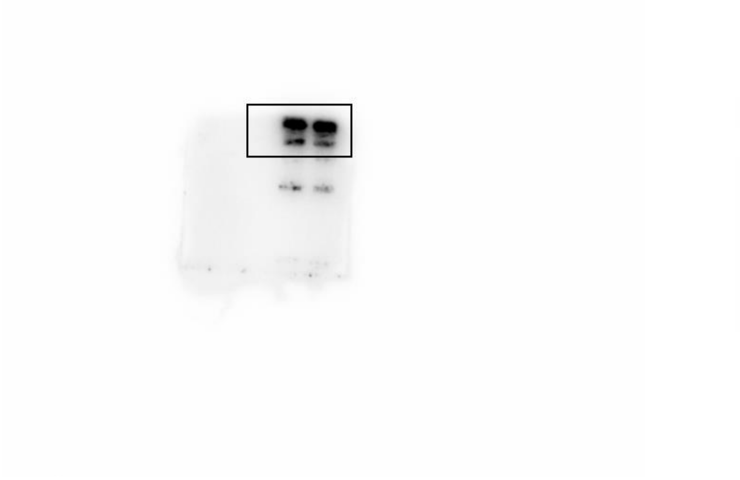

Fig. 5B IP: Falg 14-3-3τ

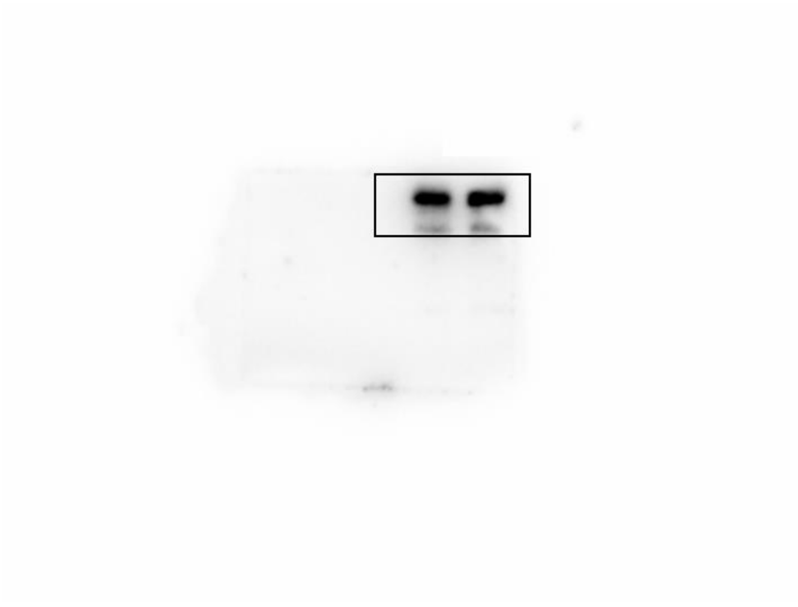

Fig. 5B IP: Falg 14-3-3γ

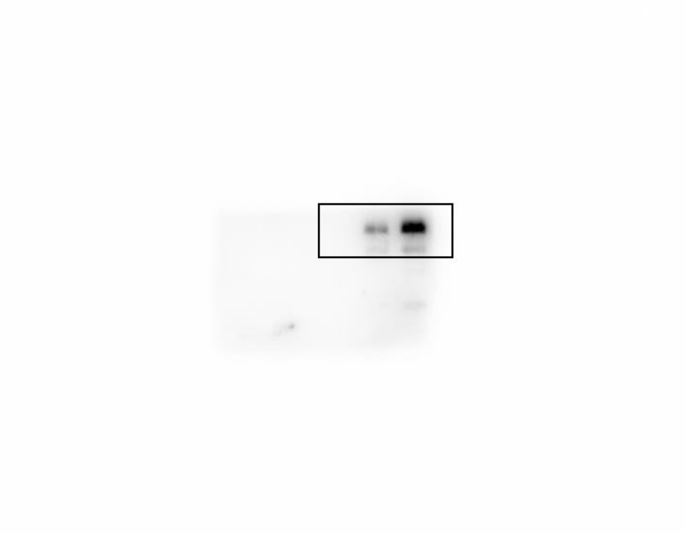

Fig. 5B IP: Falg FOXO1

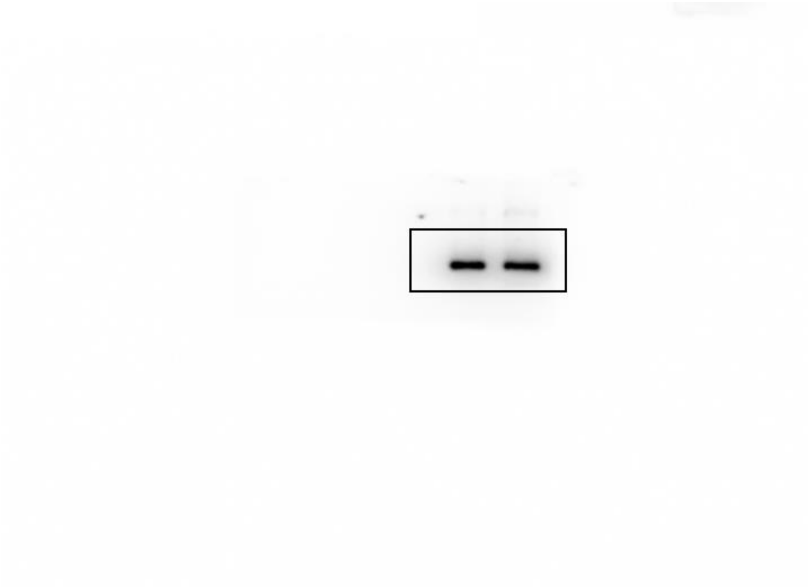

Fig. 5D

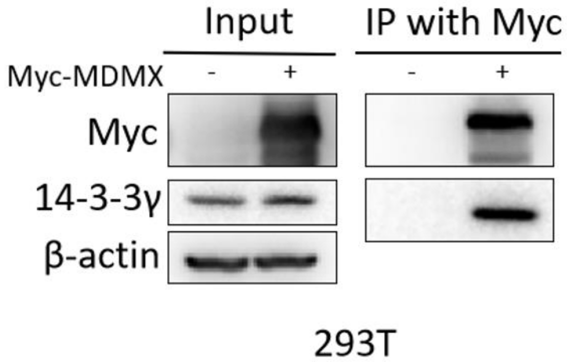

Fig. 5D Myc

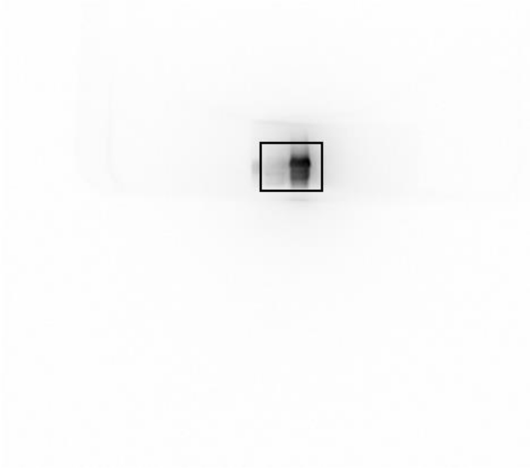

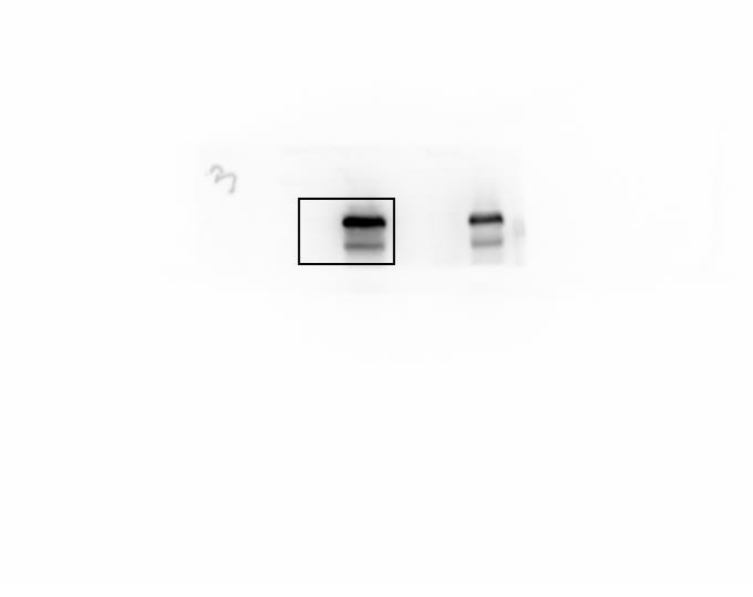

Fig. 5D 14-3-3γ

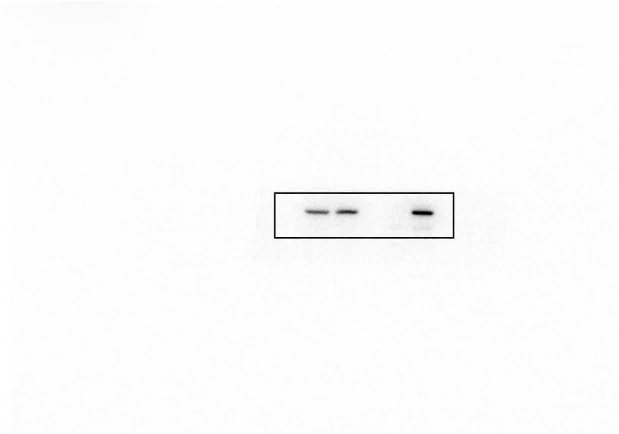

Fig. 5D actin

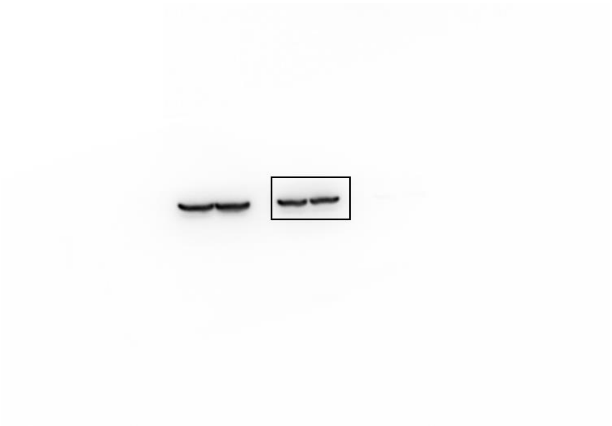

Fig. 5F

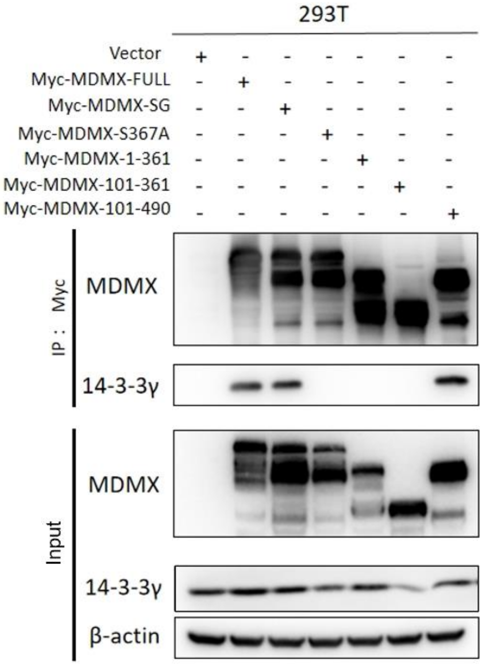

Fig. 5F IP: Myc MDMX

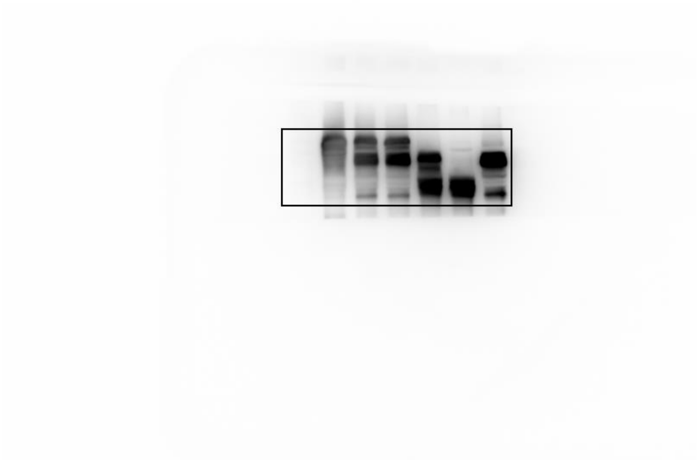

Fig. 5F IP: Myc 14-3-3γ

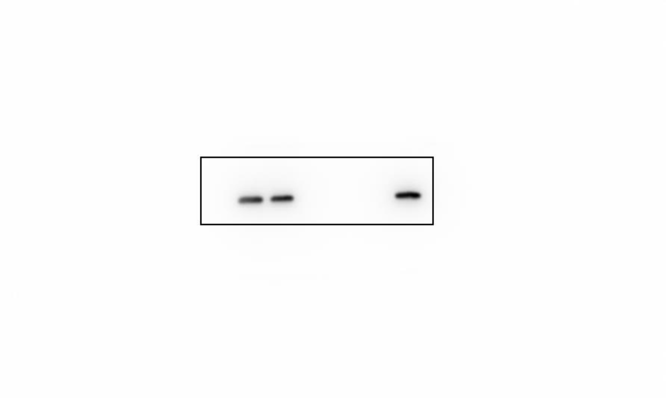

Fig. 5F Input MDMX

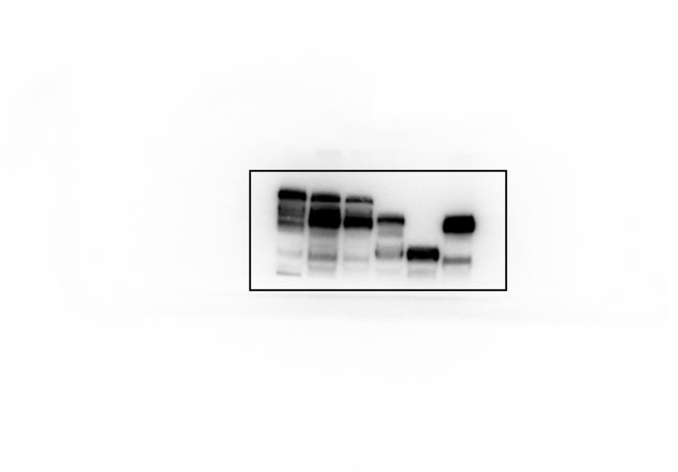

Fig. 5F Input 14-3-3γ

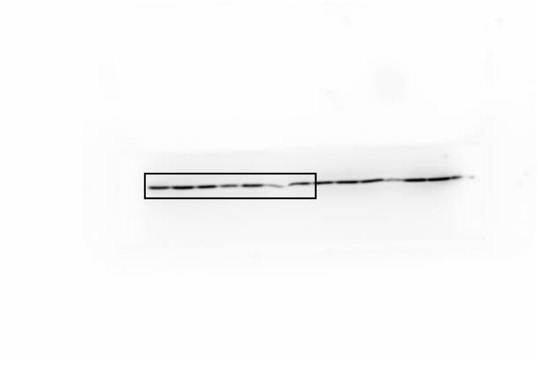

Fig. 5F Input actin

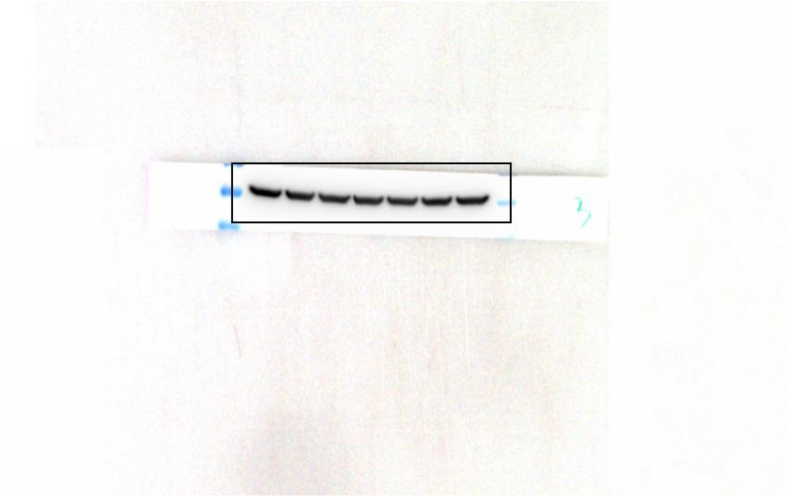

Fig. 6A

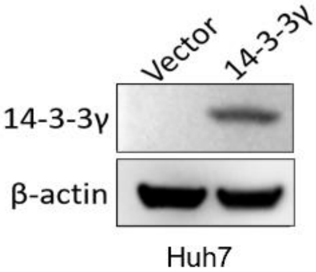

Fig. 6A-14-3-3γ

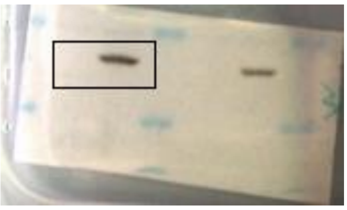

Fig. 6A-Actin

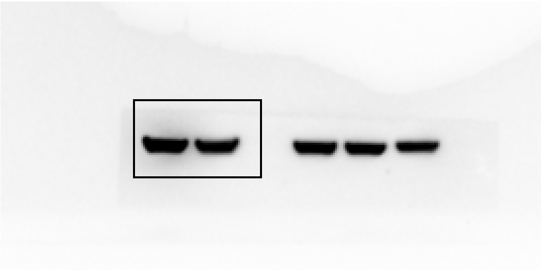

Fig. 6B (left)

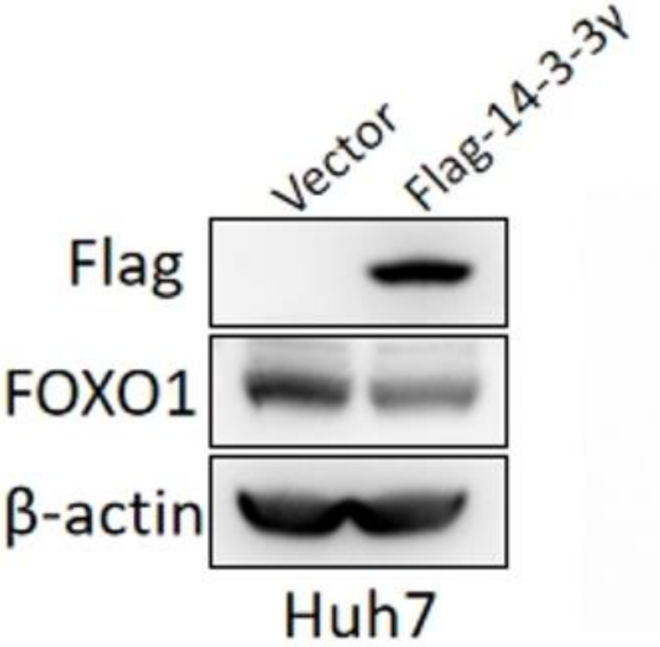

Fig. 6B (left)-Flag

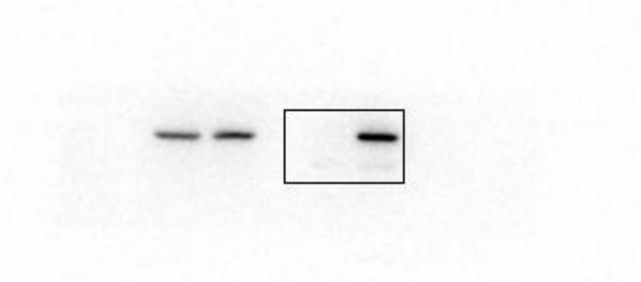

Fig. 6B (left)-FOXO1

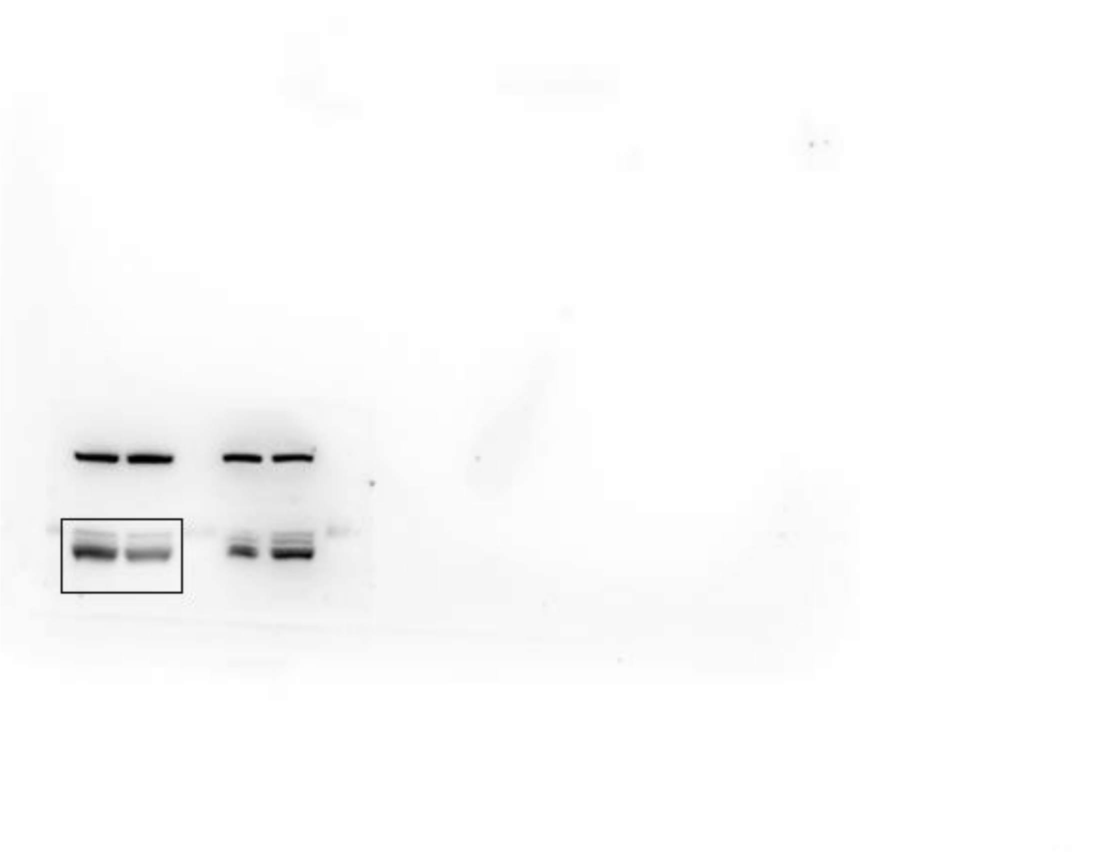

Fig. 6B (left)-Actin

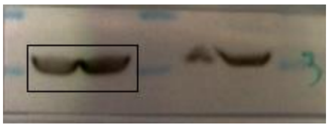

Fig. 6B (right)

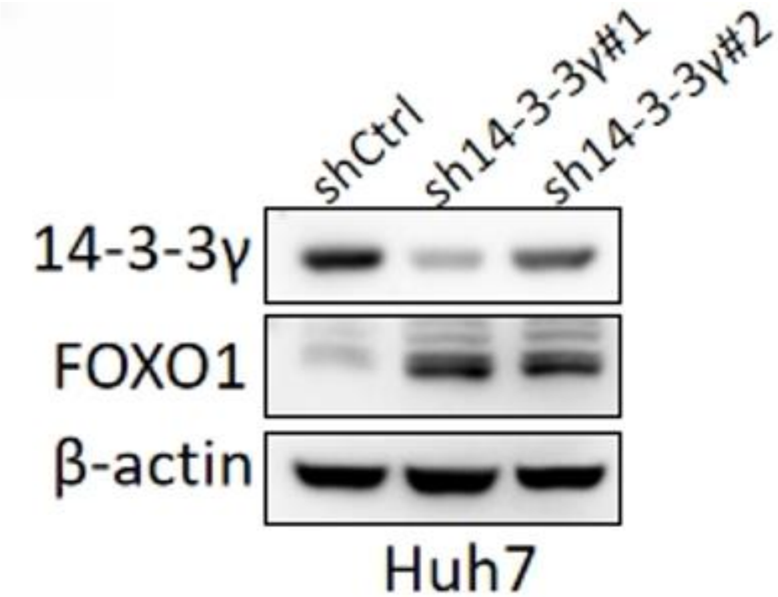

Fig. 6B (right)-14-3-3γ

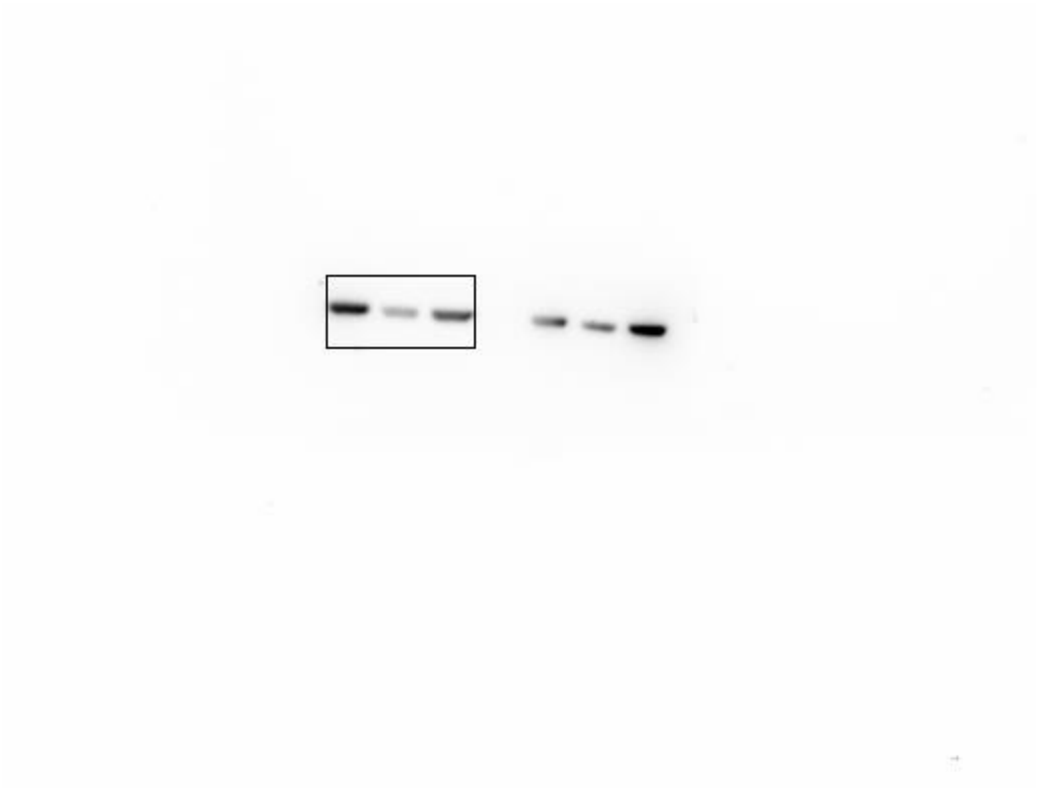

Fig. 6B (right)-FOXO1

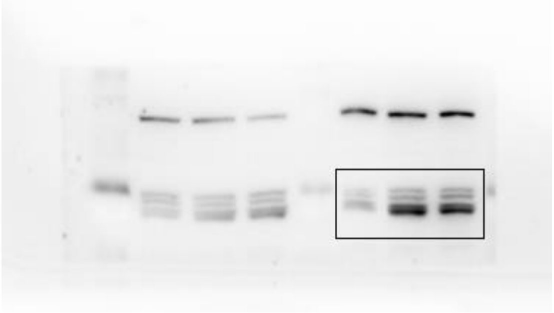

Fig. 6B (right)-Actin

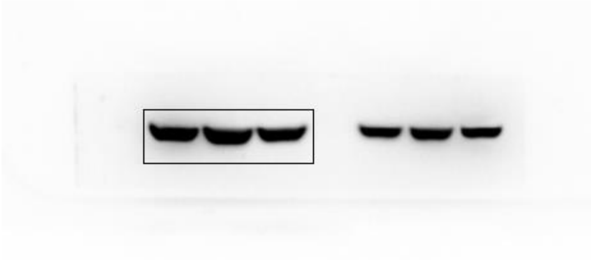

Fig. 6C

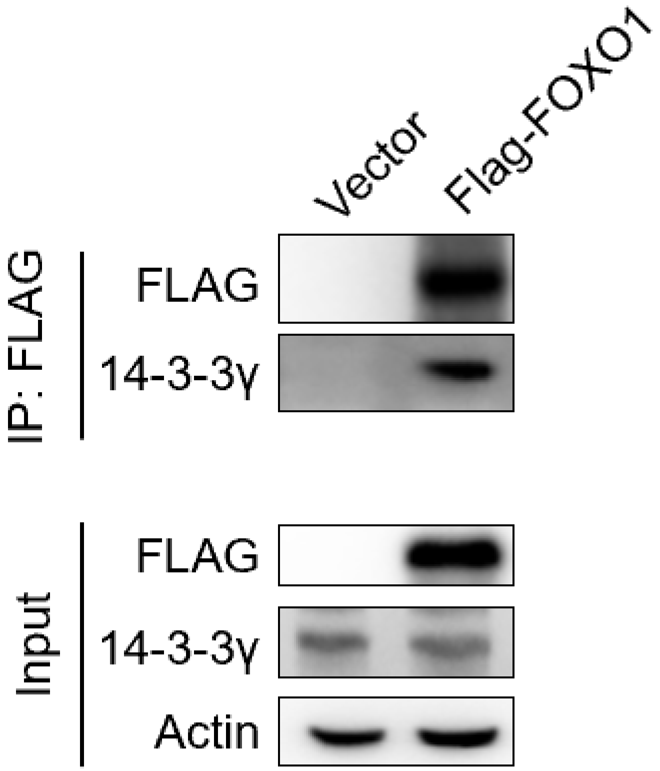

Fig. 6C IP: FLAG-FLAG

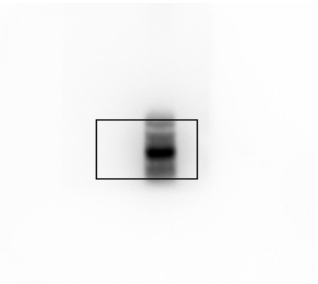

Fig. 6C IP: FLAG-14-3-3γ

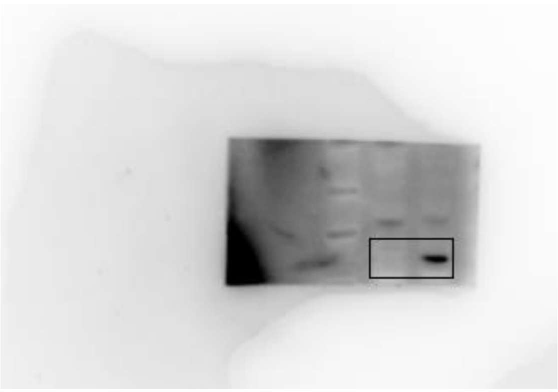

Fig. 6C Input-Flag

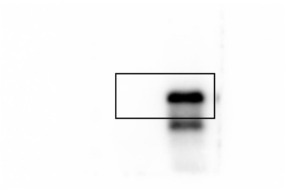

Fig. 6C Input-14-3-3γ

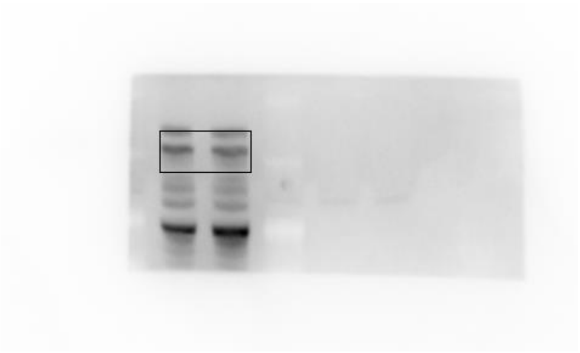

Fig. 6C Input-Actin

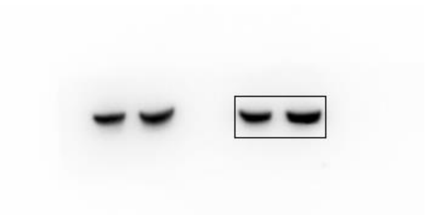

Fig. 6D

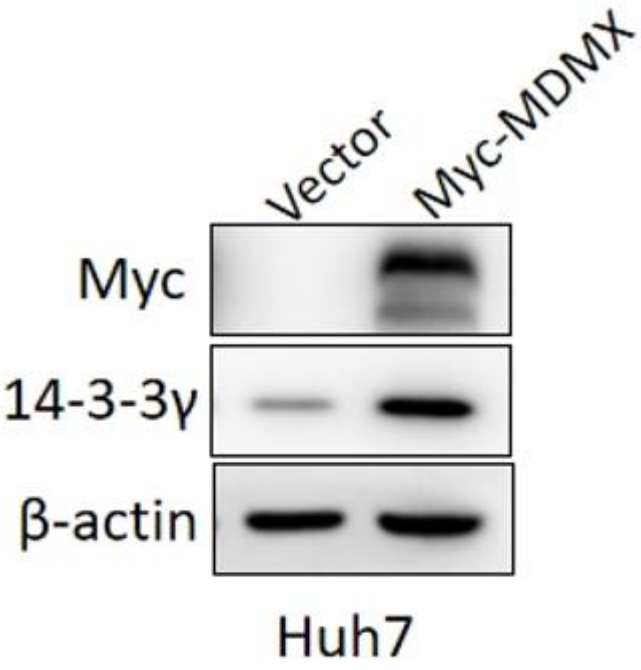

Fig. 6D-Myc

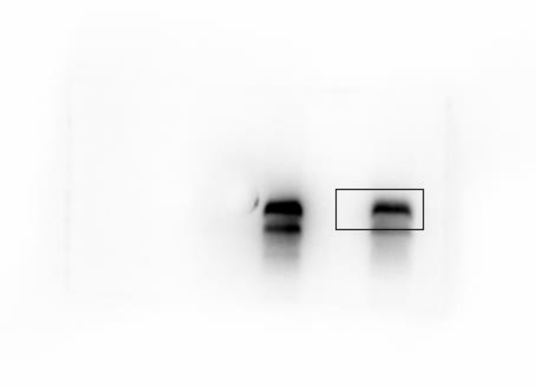

Fig. 6D-14-3-3γ

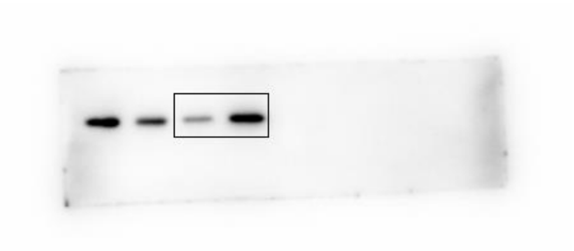

Fig. 6D-Actin

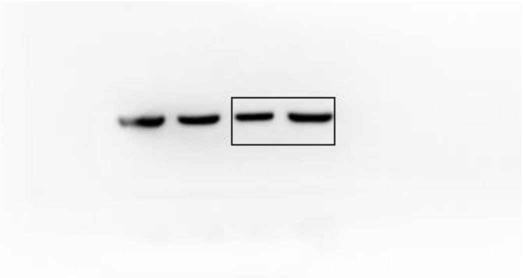

Fig. 6E

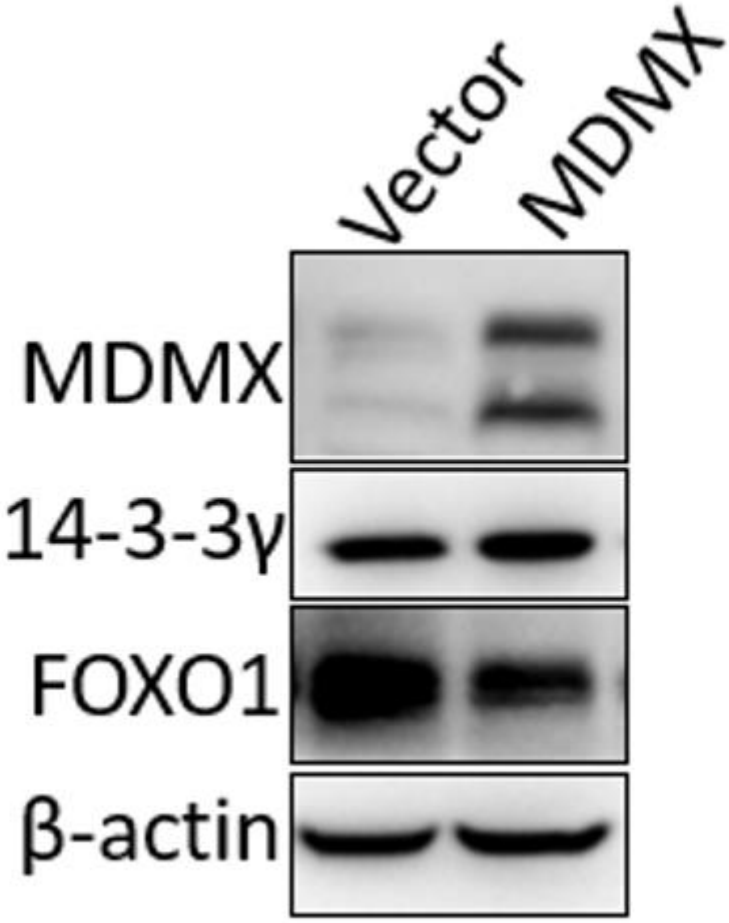

Fig. 6E-MDMX

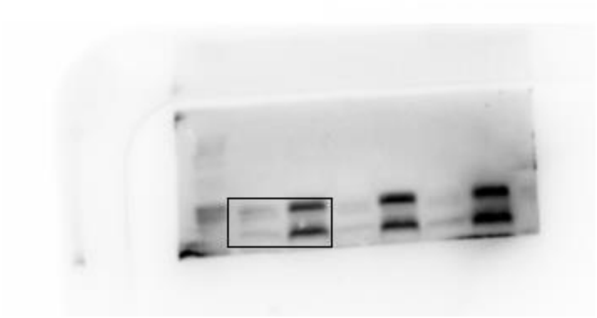

Fig. 6E-14-3-3γ

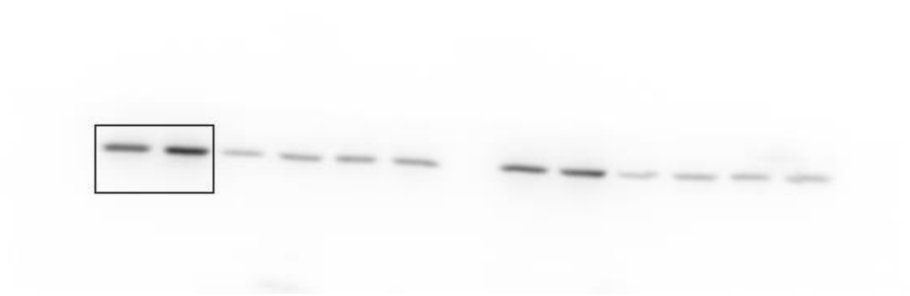

Fig. 6E-FOXO1

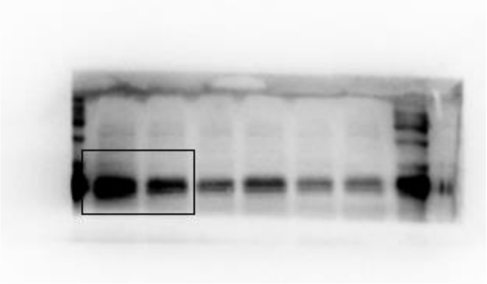

Fig. 6E-Actin

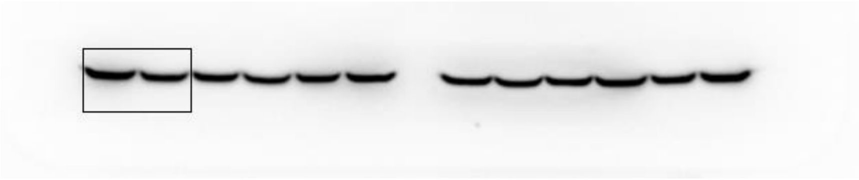

Fig. 6F

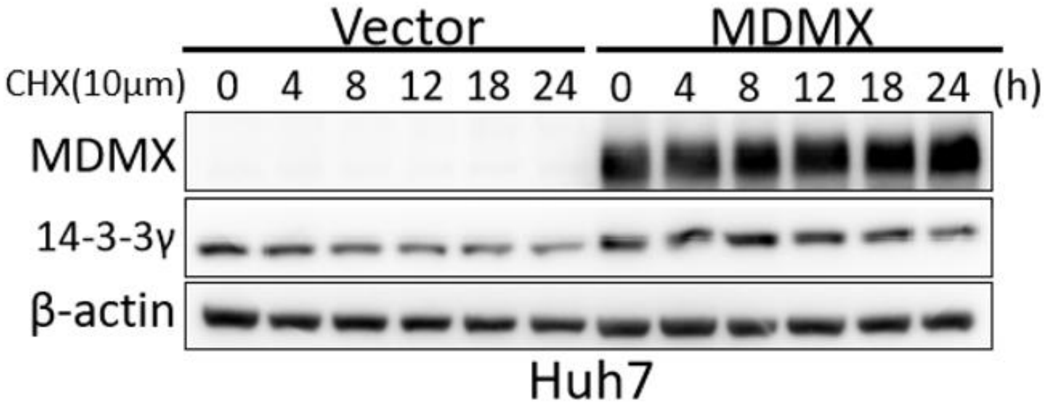

Fig. 6F-MDMX

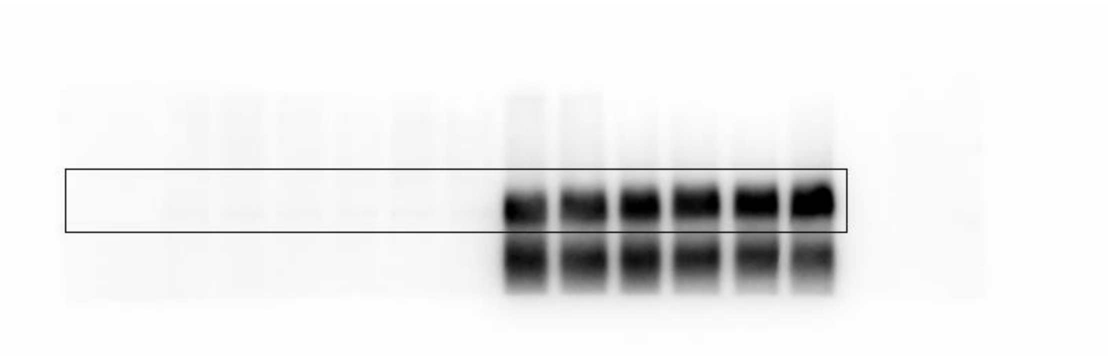

Fig. 6F-14-3-3γ

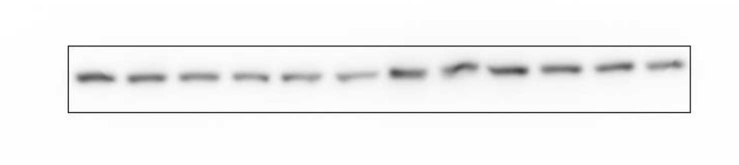

Fig. 6F-Actin

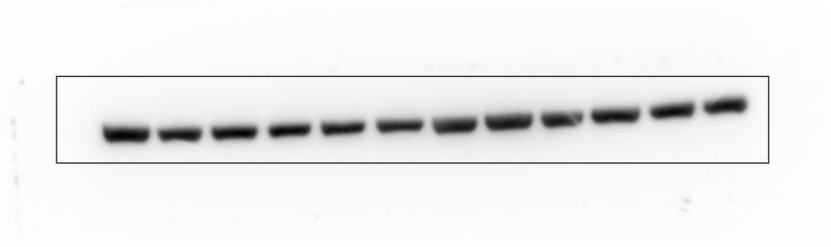

Fig. 6G

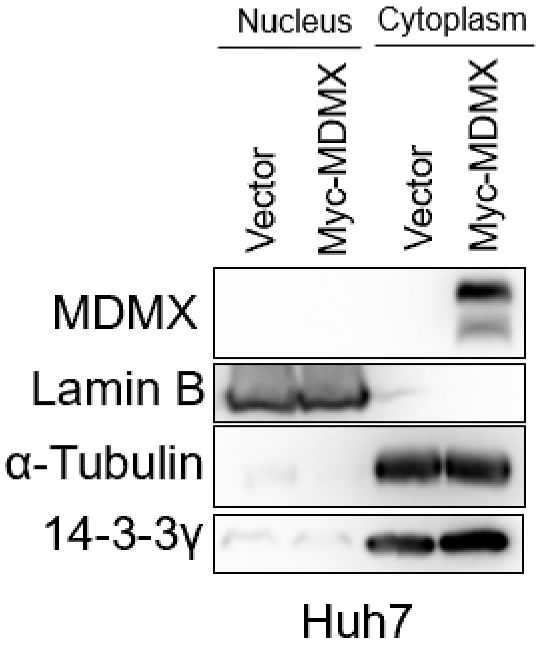

Fig. 6G-MDMX

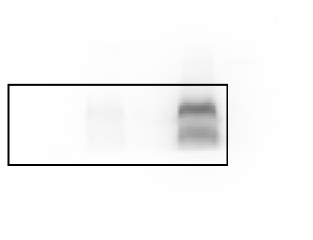

Fig. 6G-Lamin-B

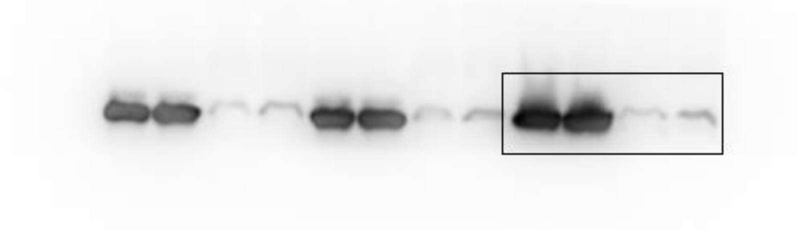

Fig. 6G-Tubulin

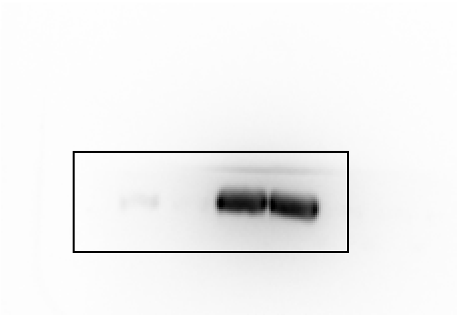

Fig. 6G-14-3-3γ

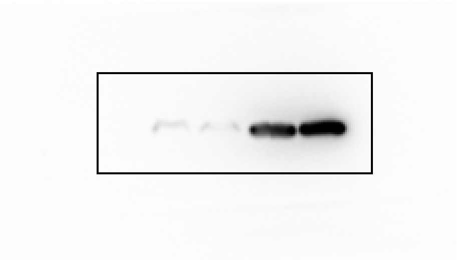

Fig. 6H

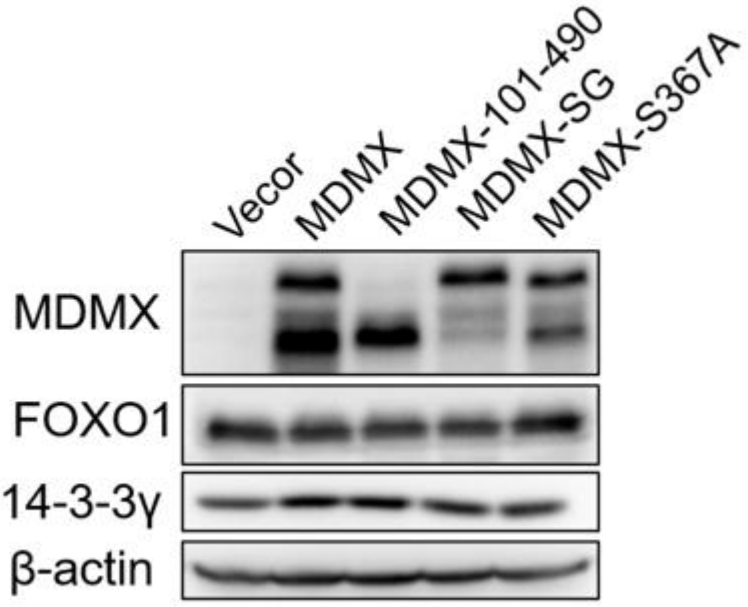

Fig. 6H-MDMX

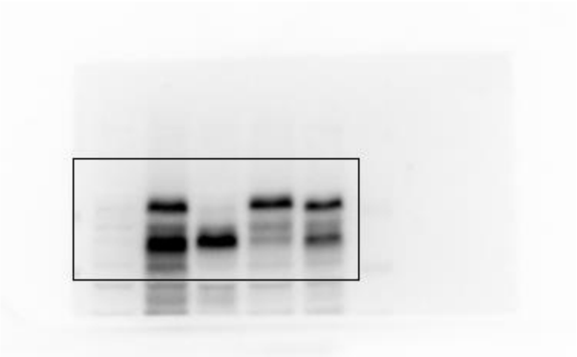

Fig. 6H-FOXO1

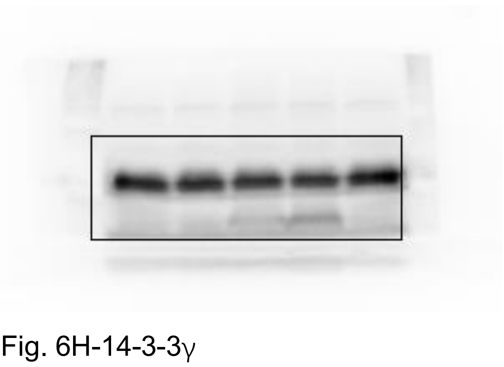

Fig. 6H-14-3-3γ

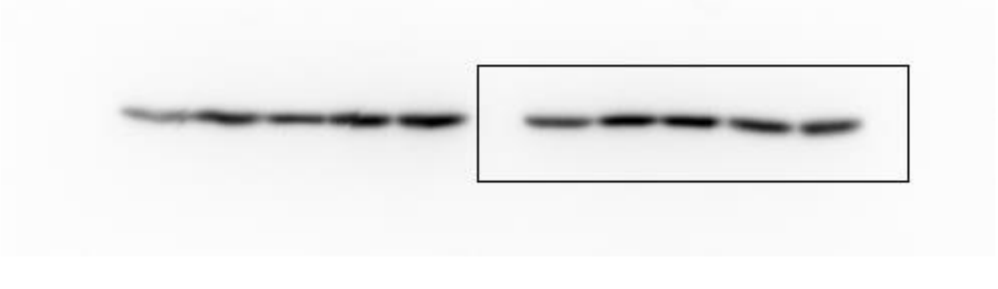

Fig. 6H-Actin

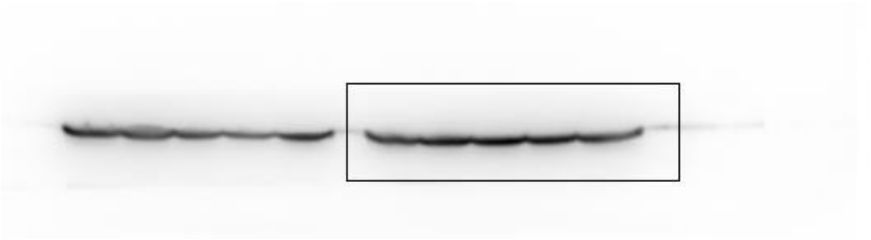

Fig. 7B

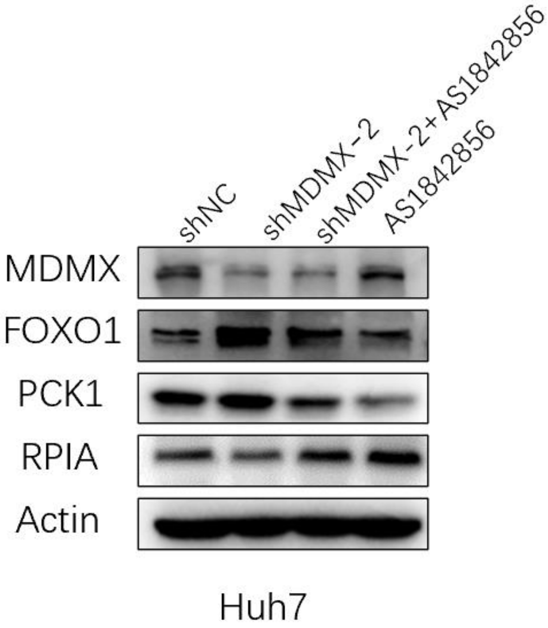

Fig. 7B MDMX

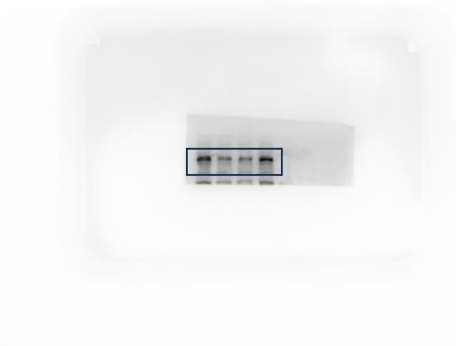

Fig. 7B FOXO1

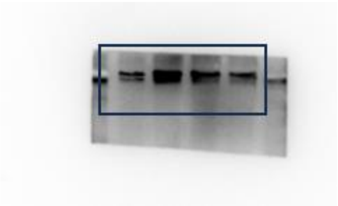

Fig. 7B PCK1

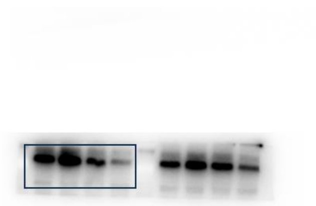

Fig. 7B RPIA

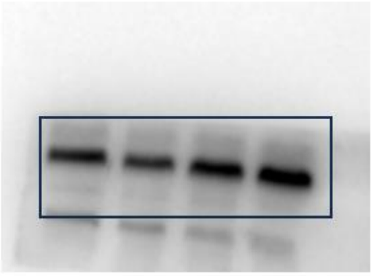

Fig. 7B Actin

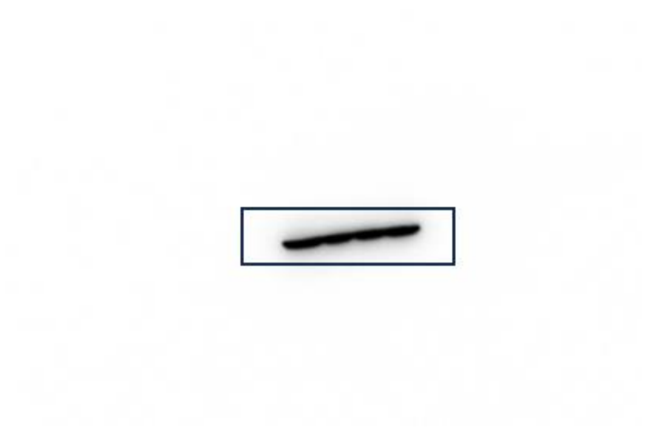

Fig. 7D

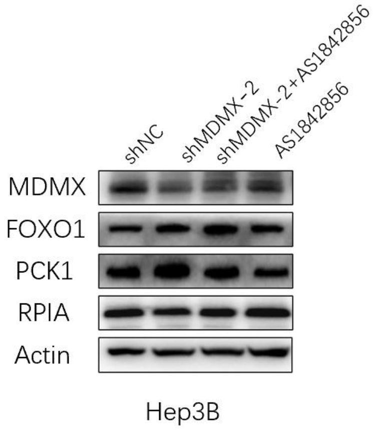

Fig. 7D MDMX

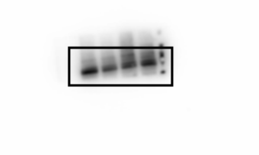

Fig. 7D FOXO1

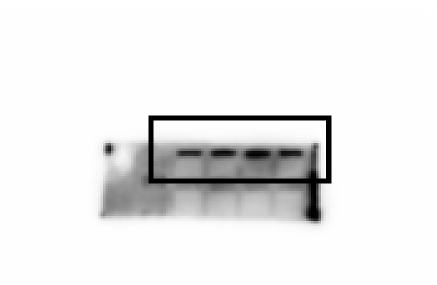

Fig. 7D PCK1

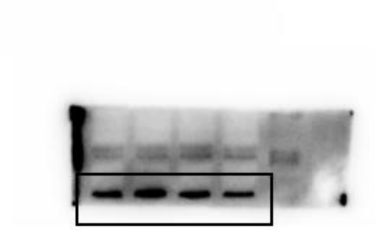

Fig. 7D RPIA

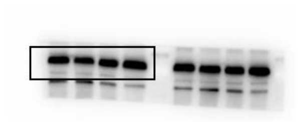

Fig. 7D Actin

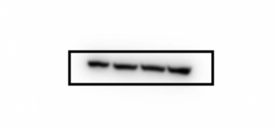

Fig. 8A

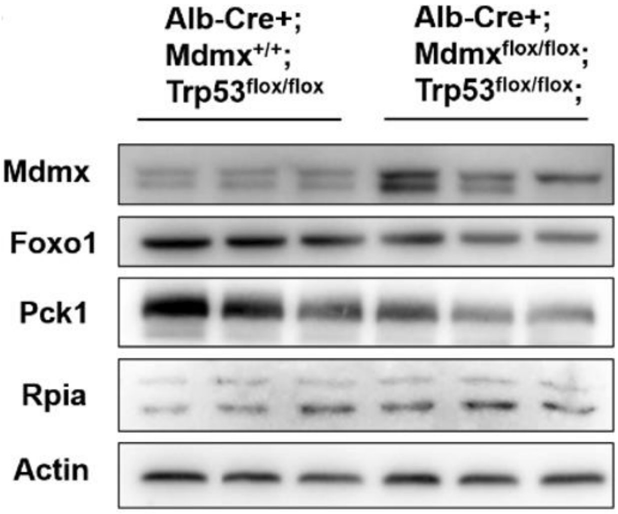

Fig. 8A Mdmx

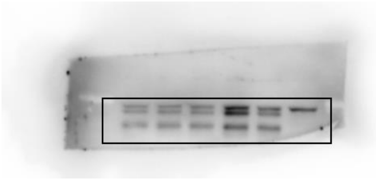

Fig. 8A Foxo1

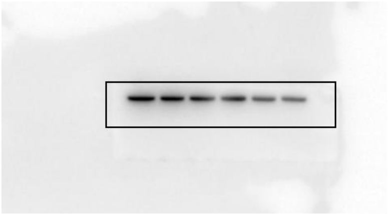

Fig. 8A Pck1

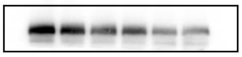

Fig. 8A Rpia

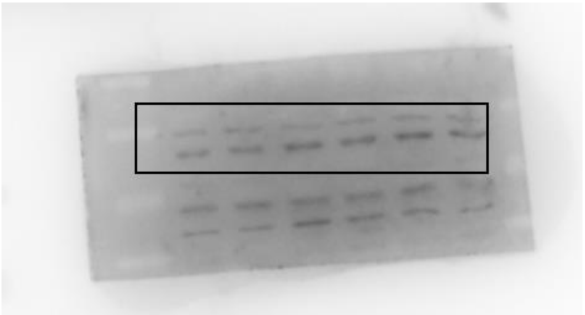

Fig. 8A Actin

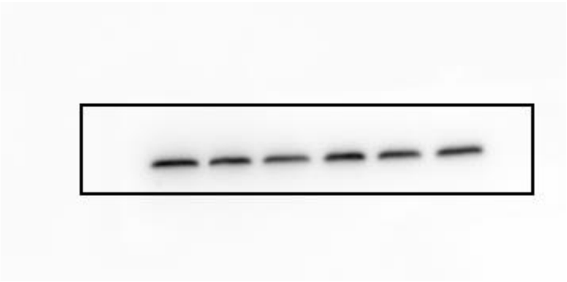

Fig. S1A

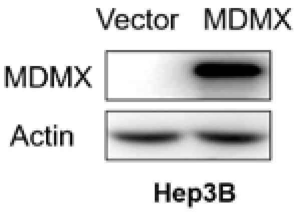

Fig. S1A MDMX

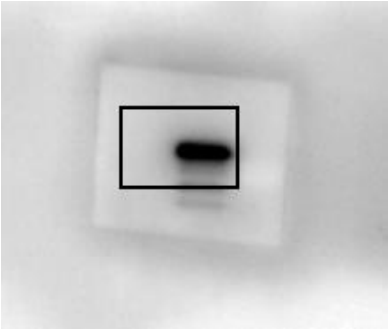

Fig. S1A Actin

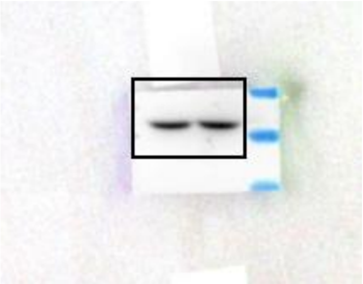

Fig. S1B

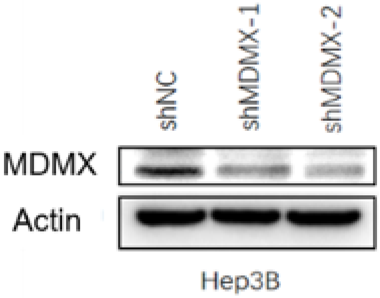

Fig. S1B MDMX

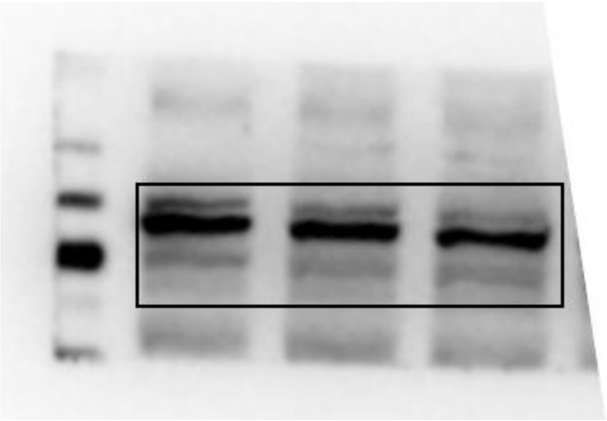

Fig. S1B Actin

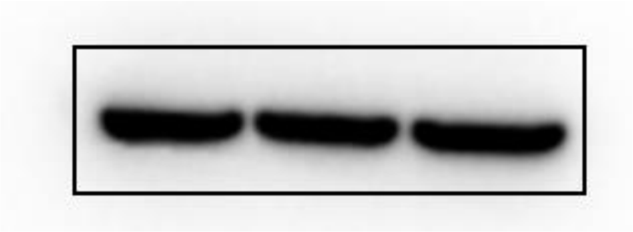

Fig. S3B MDMX OE

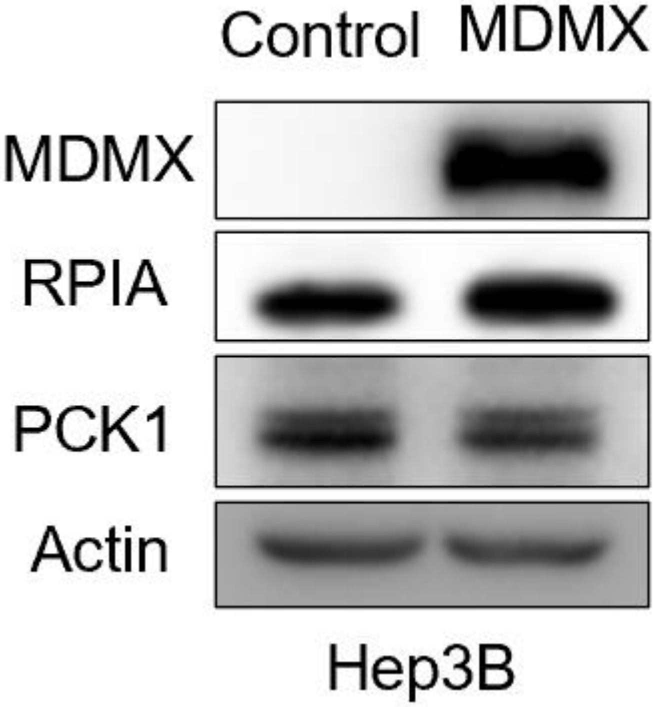

Fig. S3B MDMX OE-MDMX

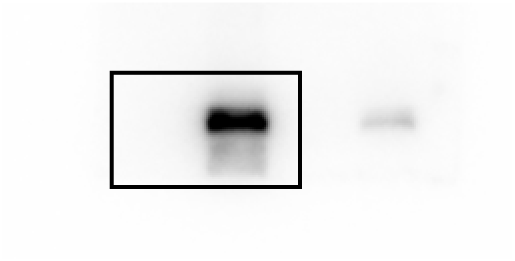

Fig. S3B MDMX OE-RPIA

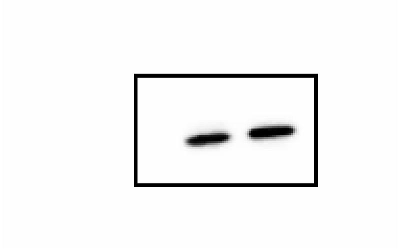

Fig. S3B MDMX OE-PCK1

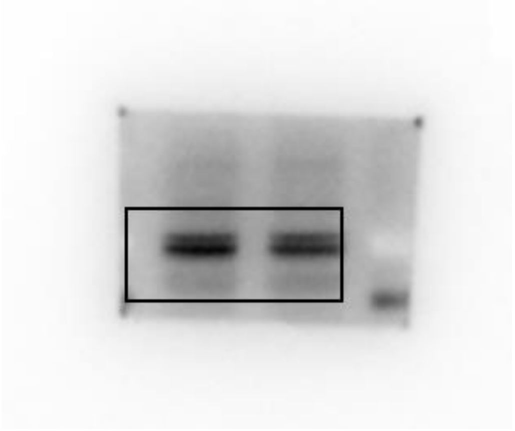

Fig. S3B MDMX OE-Actin

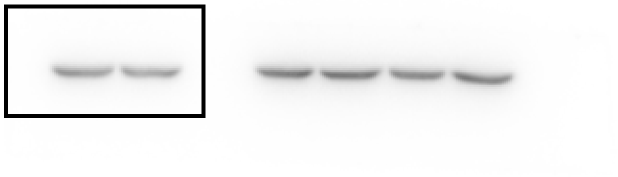

Fig. S3B shMDMX

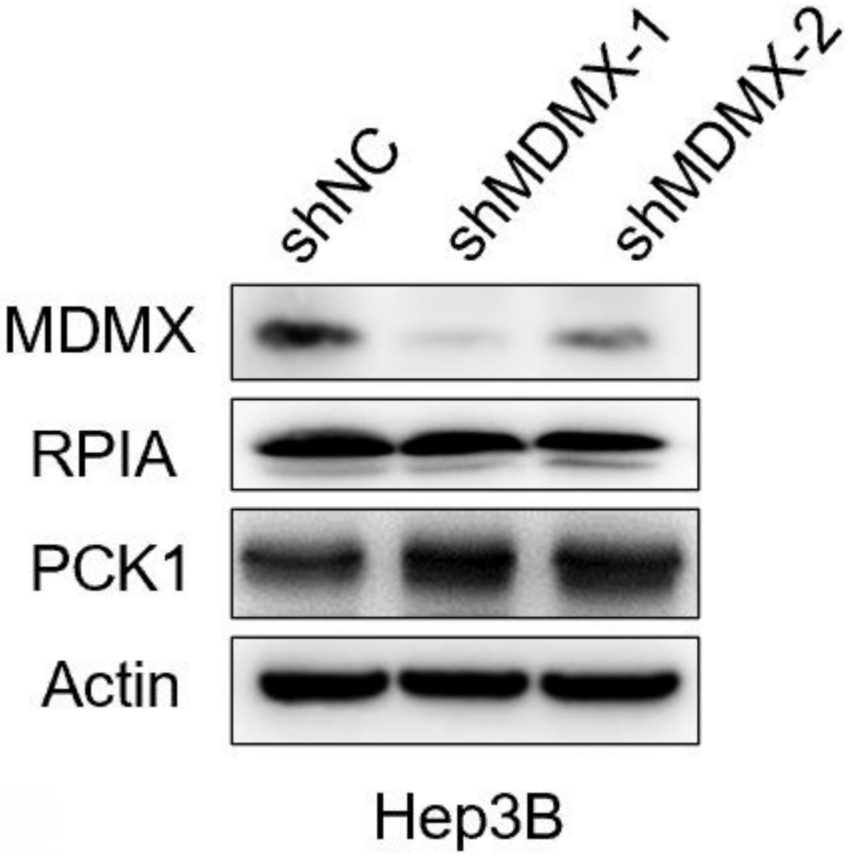

Fig. S3B shMDMX-MDMX

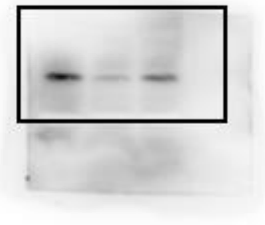

Fig. S3B shMDMX-RPIA

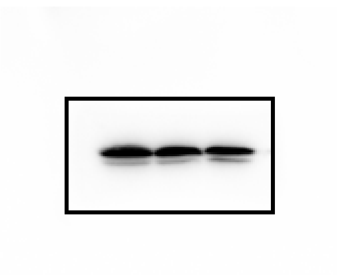

Fig. S3B shMDMX-PCK1

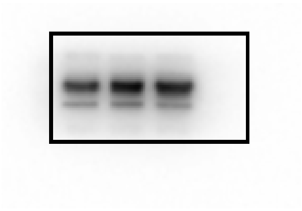

Fig. S3B shMDMX-Actin

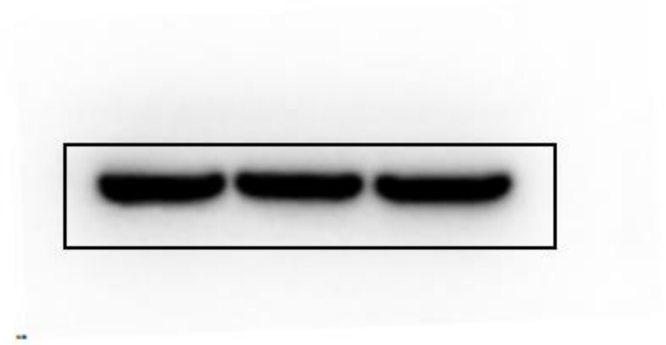

Fig. S3C

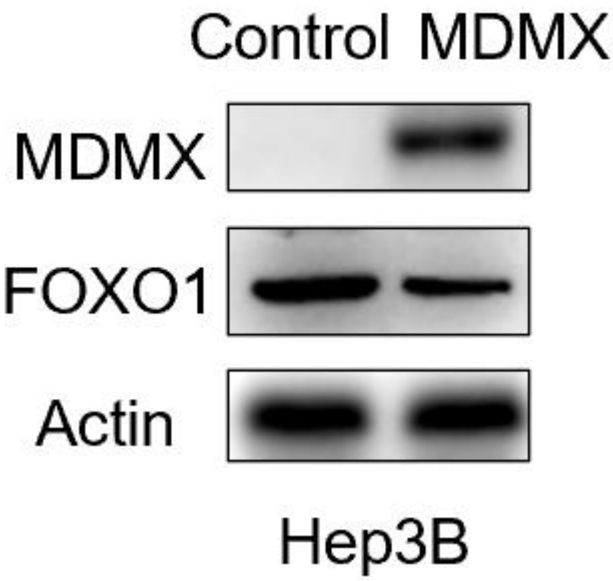

Fig. S3C MDMX

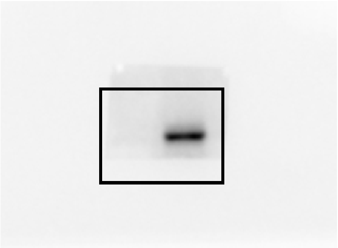

Fig. S3C FOXO1

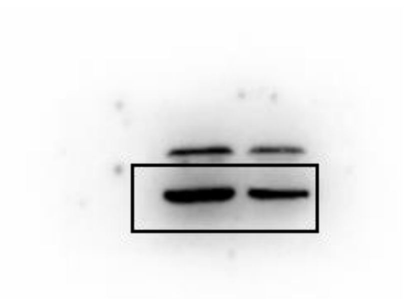

Fig. S3C Actin

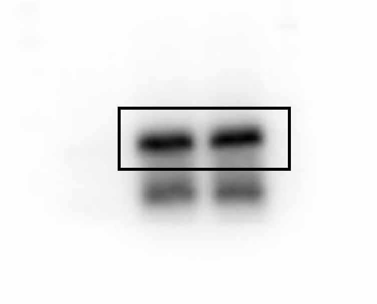

Fig. S3D

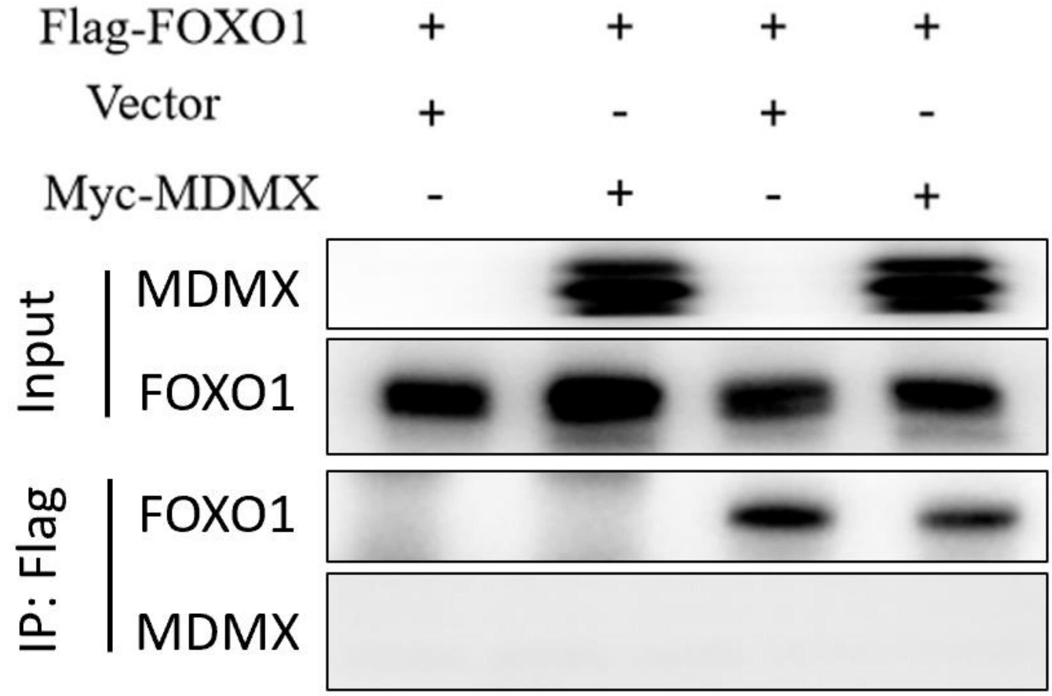

Fig. S3D Input-MDMX

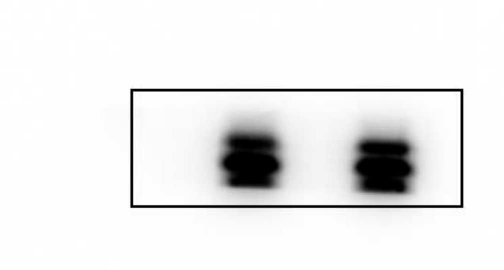

Fig. S3D Input-FOXO1

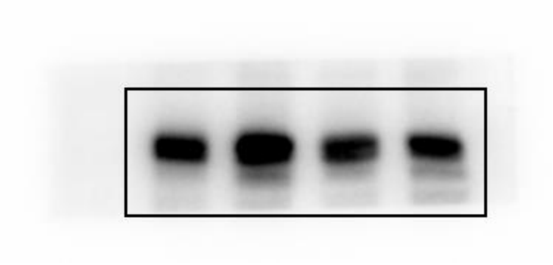

Fig. S3D IP: Flag-MDMX

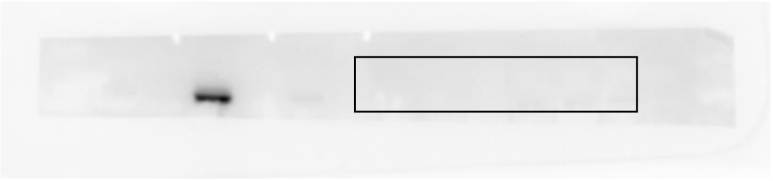

Fig. S3D IP: Flag-FOXO1

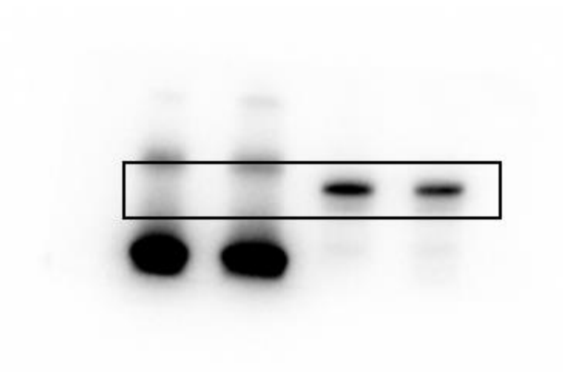

Fig. S3E

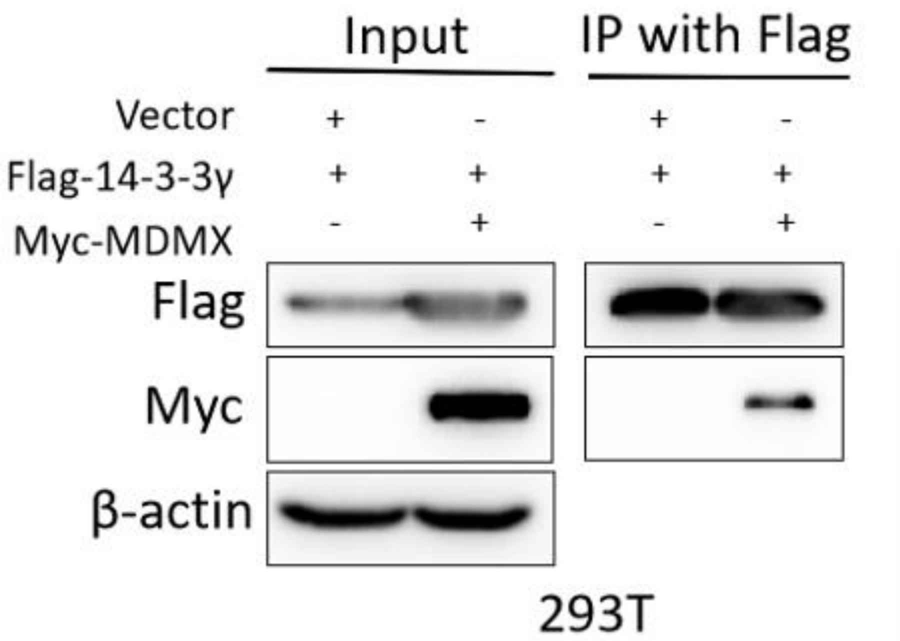

Fig. S3E-Flag

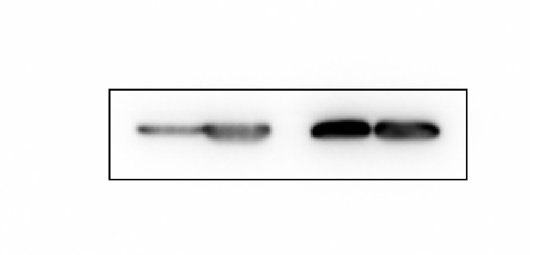

Fig. S3E-Myc

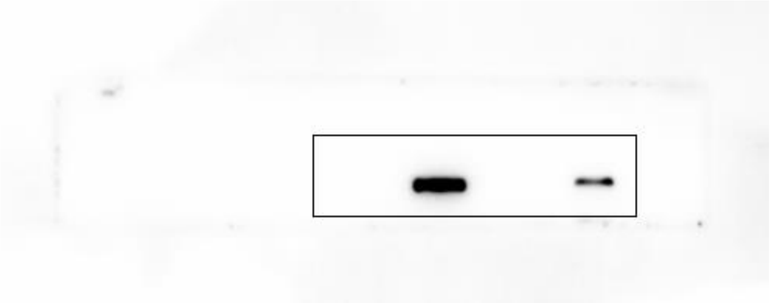

Fig. S3E-Actin

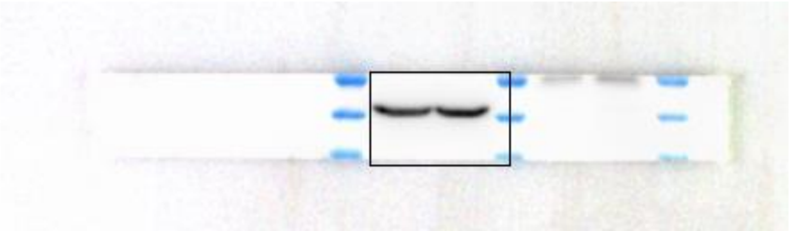

Fig. S4C

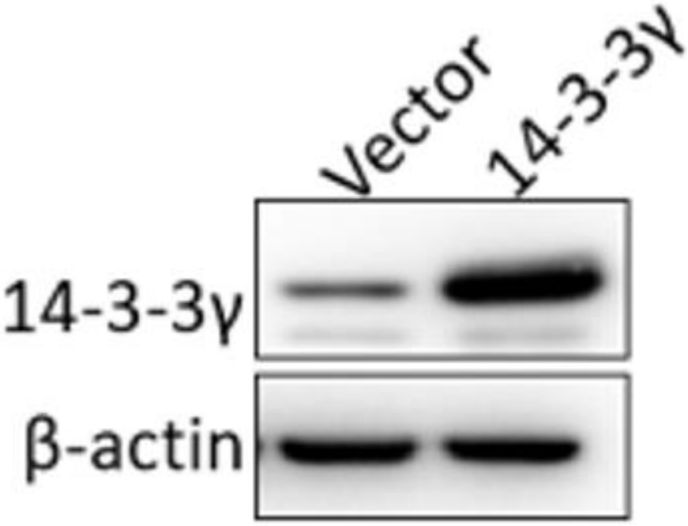

Fig. S4C-14-3-3γ

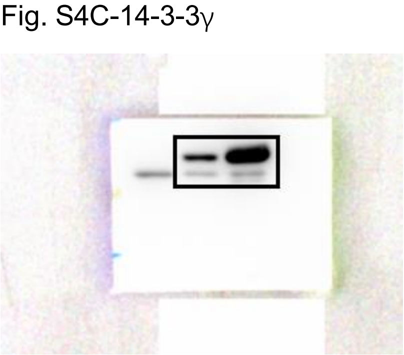

Fig. S4C-Actin

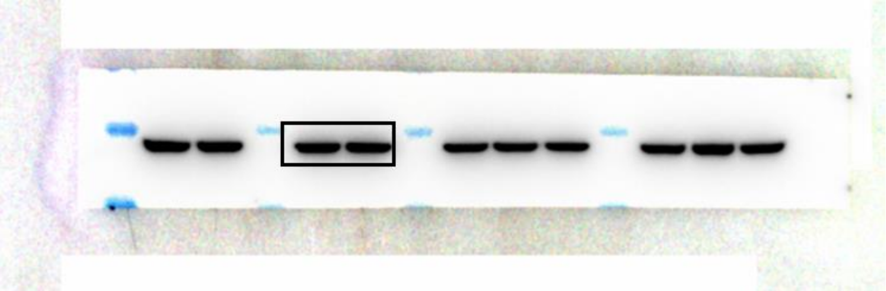

Fig. S4D

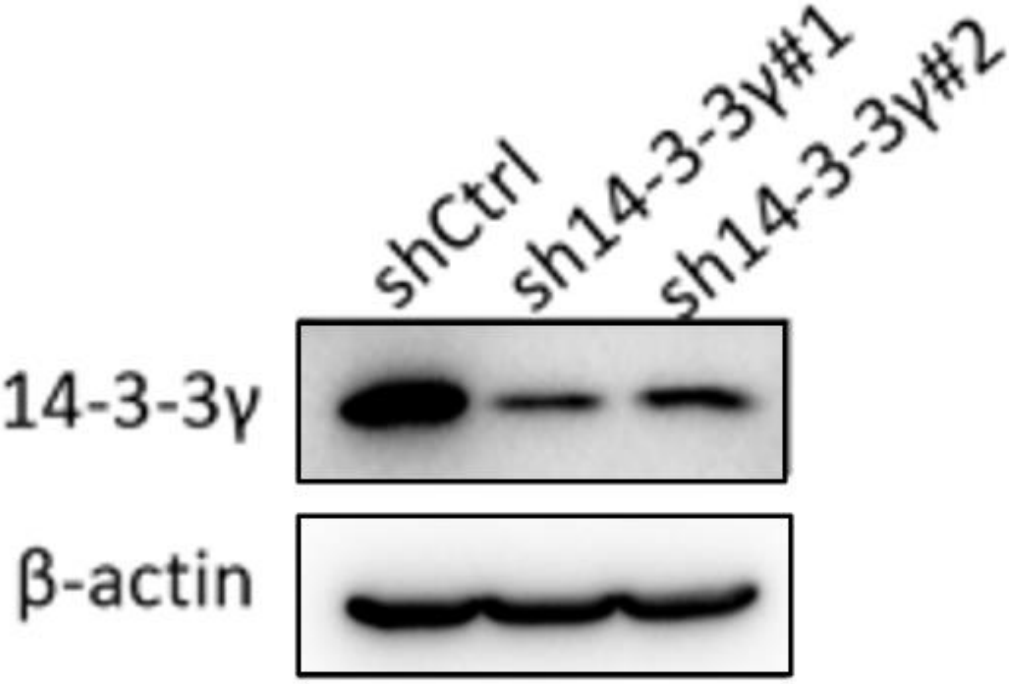

Fig. S4D-14-3-3γ

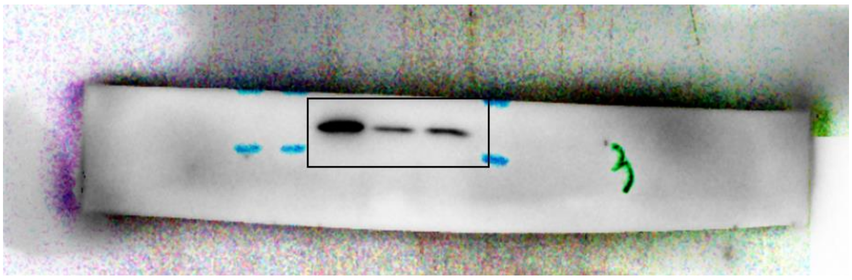

Fig. S4D-Actin

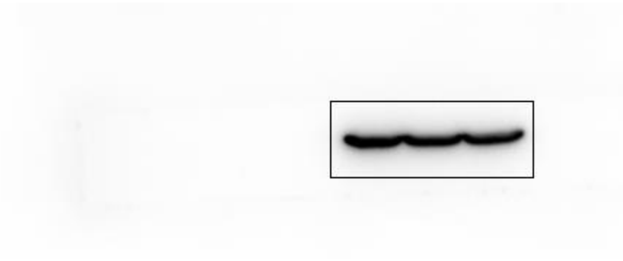

Fig. S4E

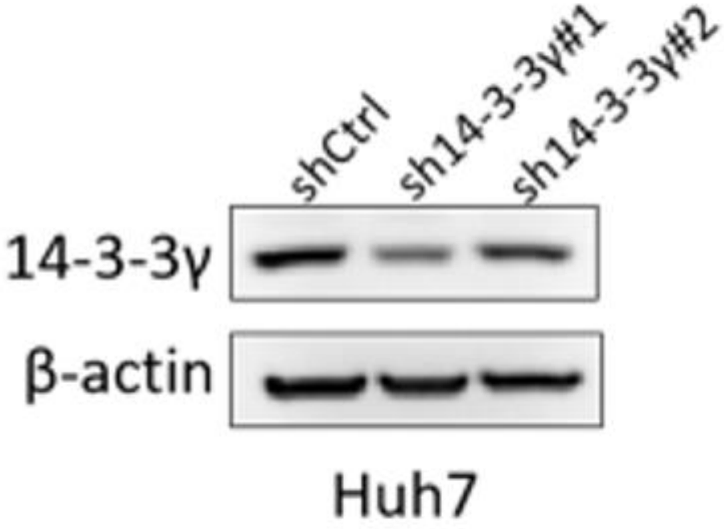

Fig. S4E-14-3-3γ

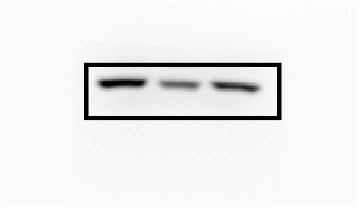

Fig. S4E-Actin

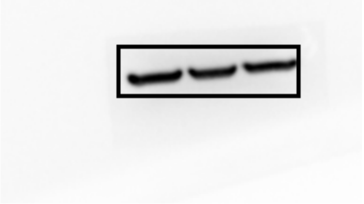

Fig. S4F (left)

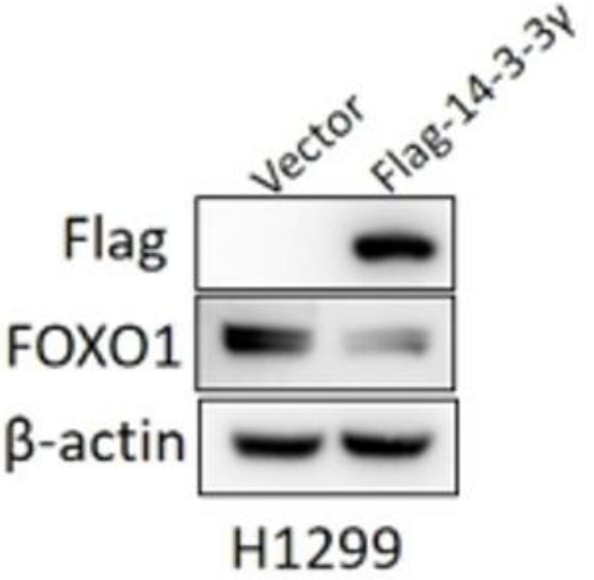

Fig. S4F (left)-Flag

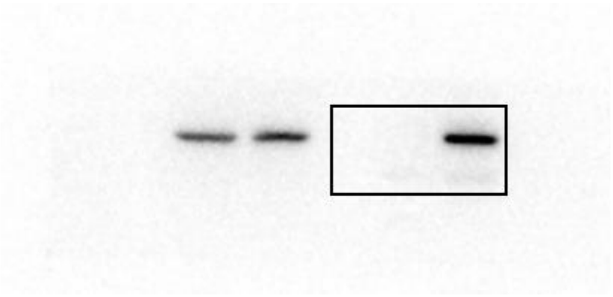

Fig. S4F (left)-FOXO1

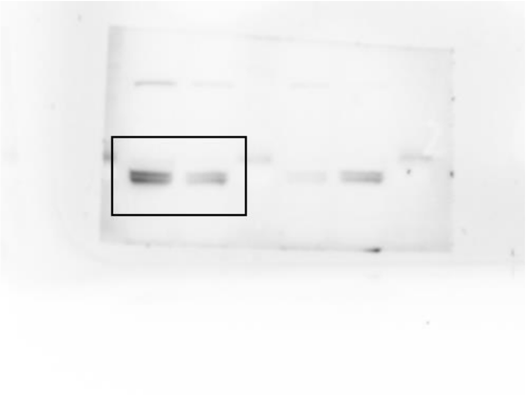

Fig. S4F (left)-Actin

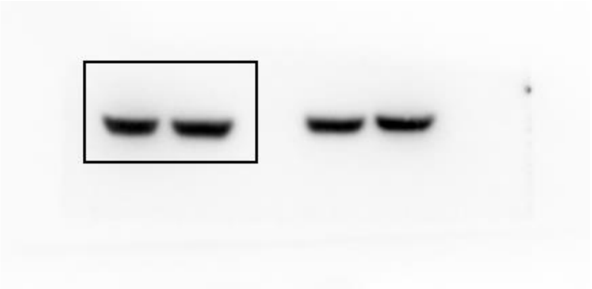

Fig. S4F (right)

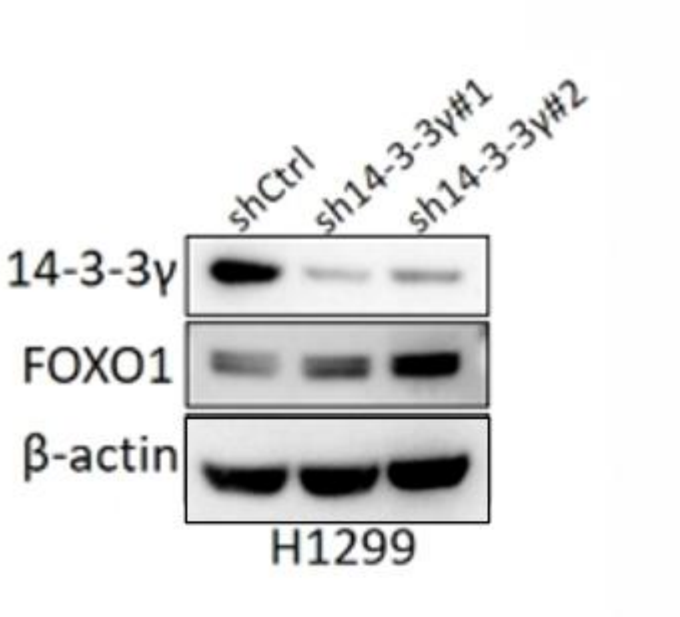

Fig. S4F (right)-14-3-3γ

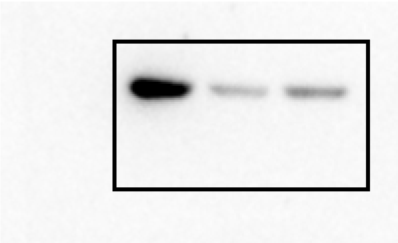

Fig. S4F (right)-FOXO1

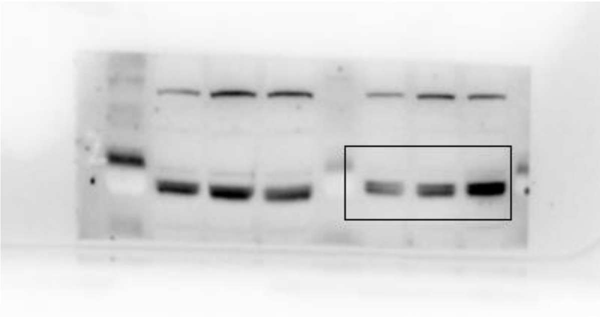

Fig. S4F (right)-Actin

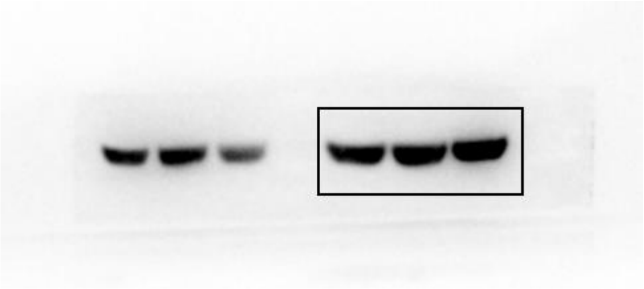

Fig. S5A

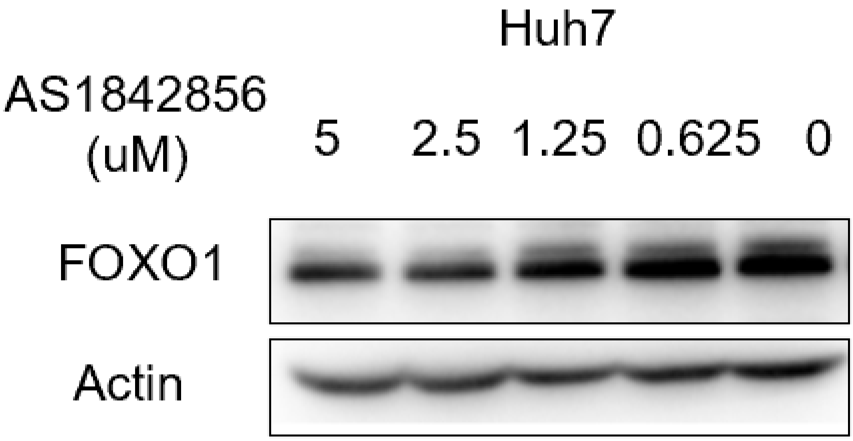

Fig. S5A-FOXO1

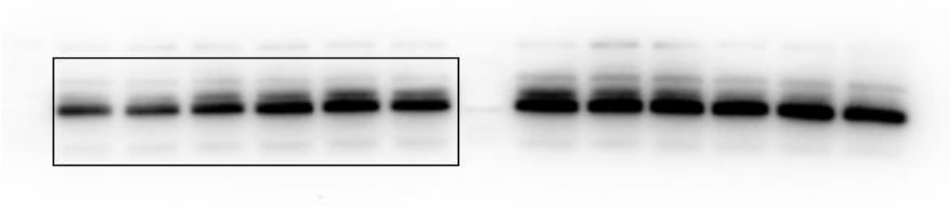

Fig. S5A-Actin

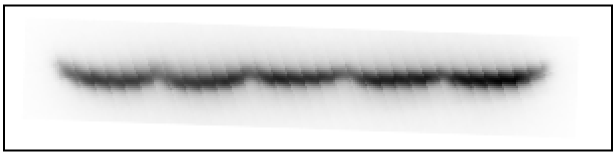

Fig. S5C

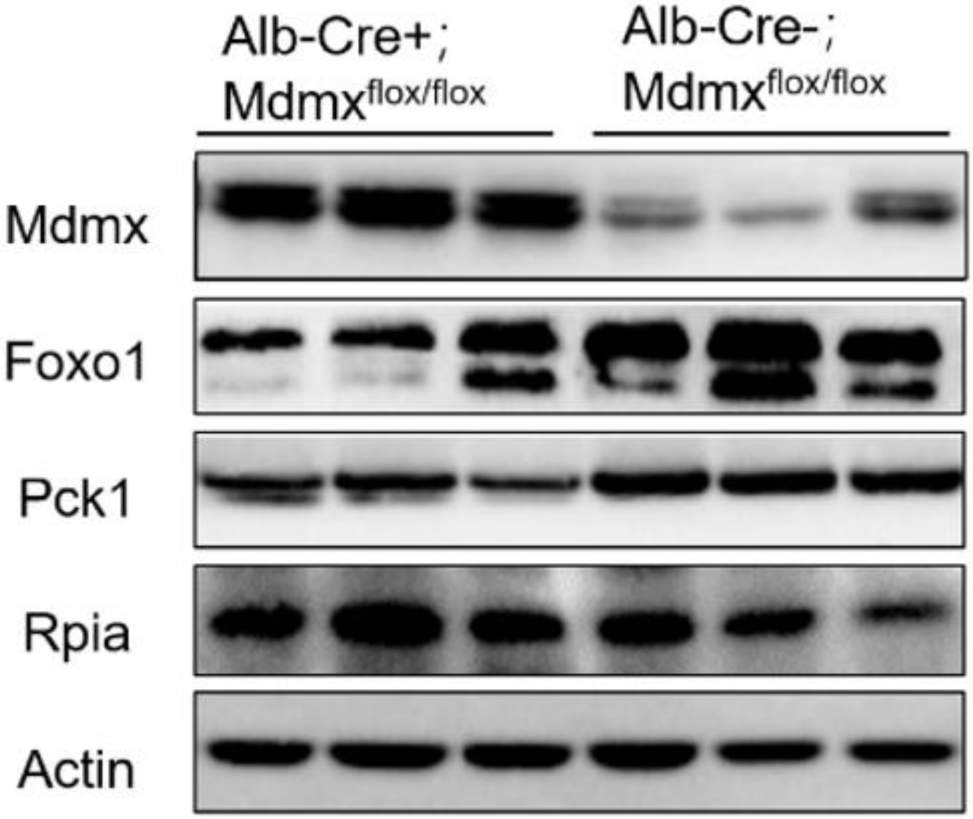

Fig. S5C-MDMX

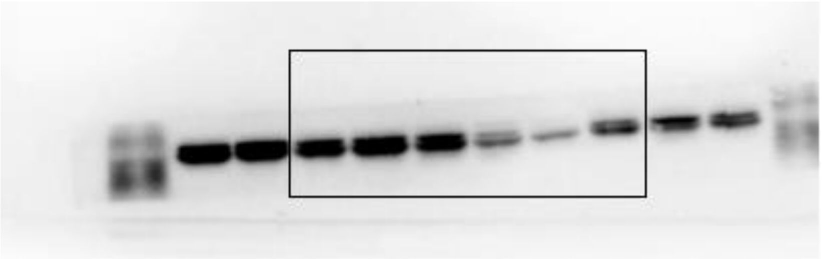

Fig. S5C-Foxo1

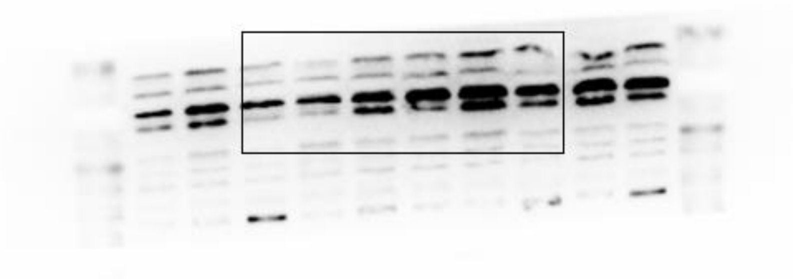

Fig. S5C-Pck1

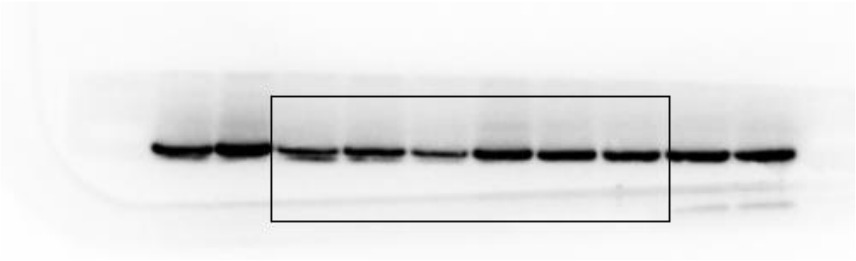

Fig. S5C-RPia

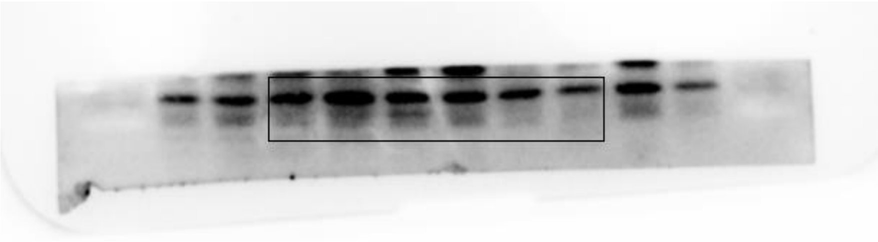

Fig. S5C-Actin

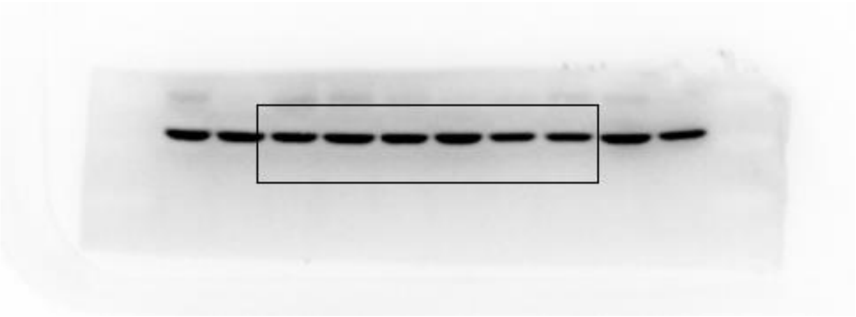

Supplement: Supplementary file 2 — Uncropped blot [file 41420_2025_2804_MOESM2_ESM.pdf]
